# Interval-valued bipolar complex fuzzy soft sets and their applications in decision making

**DOI:** 10.1038/s41598-024-58792-3

**Published:** 2024-05-21

**Authors:** Abdul Jaleel, Tahir Mahmood, Walid Emam, Shi Yin

**Affiliations:** 1 Department of Mathematics and, Stats, International Islamic University Islamabad, Islamabad, Pakistan; 2 Department of Statistics and Operations Research, Faculty of Science, King Saud University, P.O. Box 2455, 11451 Riyadh, Saudi Arabia; 3 College of Economics and Management, Hebei Agricultural University, Baoding, China

**Keywords:** Fuzzy set, Soft sets, Complex set, Bipolar complex fuzzy soft sets, Interval-valued fuzzy soft set, Aggregation operators, Decision-making approach, Applied mathematics, Pure mathematics

## Abstract

In this manuscript we demonstrate interval-valued bipolar complex fuzzy set (IVBCFS) and then interval-valued bipolar complex fuzzy soft set (IVBCFSS), as a generalization of fuzzy set, interval-valued fuzzy set, bipolar fuzzy set, complex fuzzy set and soft set. We also initiate operational laws and basic results and properties for IVBCFS and IVBCFSS. Further explanation is given for the basic algebraic operations like complement, extended union, extended intersection, restricted union, and restricted intersection, AND product and OR product for IVBCFSS. Moreover, we demonstrate some fundamental aggregation operators like IVBCFS average aggregation, IVBCFS geometric and as well as their properties. To emphasize the usefulness and application of the system, we also develop the decision-making method and joint instances of the IVBCFSS (set-ups 1 and 2). In order to describe the effectiveness and influence of the approaching novel work, this study uses a comparative analysis of the new creating concept with prevailing ideas.

## Introduction

There are a lot of problems, which are located in the complexity and vagueness. These issues are properly addressed with the decision-making (DM) approach. When people or organizations have to choose one course of action or alternative from many, decision-making problems arise. Complexity, ambiguity, several conflicting goals, and finite resources are common features of these problems. Here, we'd like to talk about some common problems. The first step is determining how to allocate limited resources, such as time, money, or staff, among numerous projects or activities. The evaluation and management of potential risks and uncertainties connected to several options or conclusions come in second. Thirdly, making decisions that will last a long time in order to achieve specific goals or objectives, like expanding operations or entering new markets. Fourth, predicting future trends or events based on the knowledge and data at hand. Fifth, determining the optimum course of action that, given the constraints and objectives, maximizes advantages or reduces disadvantages. Sixth, moral and ethical considerations are made when making decisions that may have an impact on stakeholders or offer ethical dilemmas. Seventhly, employing a range of tools and techniques for decision-making, such as decision trees, scenario analysis, or cost–benefit analysis, to make reasonable conclusions. Different contexts, such as those involving business, government, healthcare, finances, and personal life, might give rise to these problems. Acquiring relevant information, weighing options, weighing pros and disadvantages, and taking into account the impacts and outcomes of various selections are all necessary for making informed judgments. Numerous researchers, using this tool for solving a lot of issues. Edwards^1^ initiated the theory of DM. Slovic et al.^2^ signified DM. Eisenhardt and Zbaracki^3^ initiated the strategic DM. March^4^ is also propound the primer on DM.

Numerous problems are typically discovered in the ambiguity and intricacy. To overcome these obstacles, the specialists made their best effort. The fuzzy set (FS) was started by Zadeh^5^ under this conception of obstacles. The TG that makes up FS is located between [0, 1]. When academics attempt to address difficulties, new dilemmas develop as a result. There is Atanassov^6^ started intuitionistic FS (IFS), which is made up of TG and non-TG and lies in [0, 1]. The researchers started looking for a solution to these problems. There is a probability that multiple dimensions will be required to solve a problem. A complex fuzzy set (CFS) in its polar version was first introduced by Ramot et al.^6^. After this, Tamir et al.^7^ initiated CFS which is consist of real and unreal part. The addition of real and unreal are lies in [0, 1]. FS is tackled most of the dilemmas but some of them are require positive and negative aspects. Zhang^8^ demonstrated bipolar FS (BFS) which is consist of positive TG (PTG) and negative TG (NTG). The PTG lies in [0, 1] and NTG lies in [− 1, 0]. Later on, Mahmood and Rehman^9^ initiated bipolar complex FS (BCFS). BCFS is consist of $PTG+\iota PTG\in [\mathrm{0,1}]$, $NTG+\iota NTG\in [-\mathrm{1,0}]$ and ι = √(− 1) with unit square of complex plane. There are numerous aggregation operators (AOs) on the BCFS like Hamacher AOs demonstrated by Mahmood et al.^10^ and Dombi AOs signified by Mahmood and Rehman^11^.

Moreover, another thing which was more requirement for removing the hurdles in the vagueness and complexity. It’s known as soft set (SS). SS is propounded by Molodtsov^12^, which plays an important role in tackles the above dilemmas. SS is the second most prevalent notion after FS. More dilemmas are sort out by combining more than one notions like fuzzy soft set (FSS). The combination of FS and SS are demonstrated by Cagman et al.^13^, which is known as FSS. From this FSS solve more dilemmas which are required of both alternatives and attributes. Cagman and Karataş^14^ initiated intuitionistic FSS (IFSS). After that, Arora and Garg initiated a robust aggregation of IFSS. All trials of the researchers are going on and for best solutions. The picture FSS (PFSS) initiated by Dhumras and Bajaj^15^.

The efficiency of dimension Thirunavukarasu et al.^16^ propounded the theory of complex FSS (CFSS). Additionally, by adding negative aspect to FSS becomes bipolar FSS. For this, Abdullah et al.^17^ signified bipolar fuzzy soft sets (BFSS) and its consequences in DM dilemmas. For more attention, Mahmood et al.^18^ signified bipolar CFSS (BCFSS) with applications. BCFSS is consist of $PTG + \iota PTG \in \left[ {0,1} \right]$, $NTG + \iota NTG \in \left[ { - 1,0} \right]$ and $\iota = \sqrt { - 1}$ with unit square of the complex plane. Moreover, Jaleel^19^ initiated the Dombi AOs in the basis of BCFSS.

By the way, researchers want to improve the prevailing notions. Zadeh^20^ initiated interval valued FSs (IVFSs). In the interval two values are signified one lower TG (LTG) and upper TG (UTG) belong to [0, 1]. Moreover, researchers are remove the dimensional dilemmas and create a novel notion. For this, Nasir et al.^21^ demonstrated the notion of interval-valued CFSs (IVCFSs). Moreover, Yang et al.^22^ initiated the combination of IVFS and SS. Additionally, Peng et al.^23^ signified interval-valued fuzzy soft (IVFSS) DM approaches through on MABAC, SM and EDAS. Later on Wei et al.^24^ initiated multiple attribute DM (MADM) with interval-valued BFS (IVBFS). In this notion, researcher add the negative aspect to IVFS and created IVBFS. Most of the dilemmas can handle easily by the above notions but some of them are require attribute aspect. For this, Feng et al.^25^ signified interval-valued FSSs (IVFSSs) and SS level application in DM and tackled all hurdles which have created by efficiency of attributes. Furthermore, the researcher try to define AOs like, performance of the MADM approach with interval-valued spherical fuzzy Dombi AOs (IVSFDAO) initiated by Hussain et al.^26^. Liaqat et al.^27^ initiated Aczel-Alsina AOs based on Interval-valued complex single-valued neutrosophic set and their use in DM dilemmas.

In the “Preliminaries” section, we discussed the basic definitions of the prevailing notions. In the “Interval-valued bipolar complex fuzzy sets and interval-valued bipolar complex fuzzy soft sets” section, we demonstrated the definitions of IVBCFS and IVBCFSS with properties and examples. Here, we invented extended union, extended intersection, restricted union and restricted intersection of IVBCFSS with examples. Here, we also demonstrated OR and AND of IVBCFSS with their examples. In “Interval-valued bipolar complex fuzzy soft AOs” section, we demonstrated IVBCFSAA and IVBCFSGA operators with their properties. In “Multi-attribute decision making technique” section, we utilize DM technique for data and sort out the result. In “Comparative analysis” section, we compare our invented work with prevailing works and show the supremacy and dominance of our work. In “Conclusion” section, we wrap up our findings and conclusions. In the end, we provide an overview of the proposed ideas and provide examples.

## Preliminaries

In this segment, we demonstrate some basic definitions of the prevailing notions like, FS, SS, BFS, CFS, BCFS, BCFSS, IVFS, and IVFSS.

### Definition 1

(March^4^): A FS $\mathfrak{L}$ over $\k{\rm U}$ of the shape $\mathfrak{L} = \left\{ {\left( {\imath , {\rm E}_{ \mathfrak{L} } \left( \imath \right)} \right)|\imath \in \k{\rm U} } \right\}$ where ${\rm E}_{ \mathfrak{L} } \left( \imath \right):\k{\rm U} \to \left[ {0, 1} \right]$ is TG.

### Definition 2

(Mahmood and Rehman^11^): Let $\k{\rm U}$ be the set of universe, the set of parameter $\Upsilon$ and $\Gamma \subseteq \Upsilon$, the pair $\left( { \mathfrak{L} ,{ }\Gamma } \right)$ is known as SS, where $\mathfrak{L} :\Gamma \to `{P}\left( {\k{\rm U} } \right)$. $`{P}\left( {\k{\rm U} } \right)$ is the power set of $\k{\rm U}$.

### Definition 3

(Tamir et al.^7^): A BFS $\mathfrak{L}$ over $\k{\rm U}$ is of the shape $\mathfrak{L} = \left\{ {\left( {\imath , {\rm E}_{ \mathfrak{L} }^{ + } \left( \imath \right), \;{\rm E}_{ \mathfrak{L} }^{ - } \left( \imath \right)} \right)|\imath \in \k{\rm U} } \right\}$ where ${\rm E}_{{\mathfrak{L}}}^{ + } :\k{\rm U} \to \left[ {0, 1} \right]$, ${\rm E}_{{\mathfrak{L}}}^{ - } :\k{\rm U} \to \left[ { - 1, 0} \right]$ are PTG and NTG.

### Definition 4

(Zadeh^5^): A CFS $\mathfrak{L}$ over $\k{\rm U}$ is of the shape $\mathfrak{L} = \left\{ {\left( {\imath , {\rm E}_{ \mathfrak{L} } \left( \imath \right)} \right)|\imath \in \k{\rm U} } \right\} = \left\{ {\left( {\imath , {\rm N}_{ \mathfrak{L} } \left( \imath \right) + \iota {\rm O}_{ \mathfrak{L} } \left( \imath \right)} \right)|\imath \in \k{\rm U} } \right\}$, where ${\rm E}_{ \mathfrak{L} } \left( \imath \right)$ is TG , ${\rm N}_{ \mathfrak{L} } \left( \imath \right), {\rm O}_{ \mathfrak{L} } \left( \imath \right) \in \left[ {0, 1} \right]$ and $\iota = \sqrt { - 1}$.

### Definition 5

(Zhang^8^): A BCFS $\mathfrak{L}$ is of the shape

$$ \mathfrak{L} = \left\{ {\left( {\imath , {\rm E}_{ \mathfrak{L} }^{ + } \left( \imath \right), {\rm E}_{ \mathfrak{L} }^{ - } \left( \imath \right)} \right)|\imath \in \k{\rm U} } \right\} = \left\{ {\left( {\imath , {\rm N}_{ \mathfrak{L} }^{ + } \left( \imath \right) + \iota {\rm O}_{ \mathfrak{L} }^{ + } \left( \imath \right), {\rm N}_{ \mathfrak{L} }^{ - } \left( \imath \right) + \iota {\rm O}_{ \mathfrak{L} }^{ - } \left( \imath \right)} \right)|\imath \in \k{\rm U} } \right\} $$

where ${\rm E}_{ \mathfrak{L} }^{ + } \left( \imath \right) = {\rm N}_{ \mathfrak{L} }^{ + } \left( \imath \right) + \iota {\rm O}_{ \mathfrak{L} }^{ + } \left( \imath \right)$ denote PTG and ${\rm E}_{ \mathfrak{L} }^{ - } \left( \imath \right) = {\rm N}_{ \mathfrak{L} }^{ - } \left( \imath \right) + \iota {\rm O}_{ \mathfrak{L} }^{ - } \left( \imath \right)$ denotes NTG. The values of ${\rm E}_{ \mathfrak{L} }^{ + } \left( \imath \right)$ and ${\rm E}_{ \mathfrak{L} }^{ - } \left( \imath \right)$ can get all values are belong in the unit square of complex plane, $\iota = \sqrt { - 1}$, ${\rm N}_{ \mathfrak{L} }^{ + } \left( \imath \right), {\rm O}_{ \mathfrak{L} }^{ + } \left( \imath \right) \in \left[ {0, 1} \right]$ and ${\rm N}_{ \mathfrak{L} }^{ - } \left( \imath \right), {\rm O}_{ \mathfrak{L} }^{ - } \left( \imath \right) \in \left[ { - 1, 0} \right]$.

### Definition 6

(Abdullah et al.^17^): Let $\k{\rm U}$ be the set universe, the set of parameter $\Upsilon$ and $\Gamma \subseteq \Upsilon$, the pair $\left( {{\mathfrak{L}},{ }\Gamma } \right)$ is known as a BCFSS over $\k{\rm U}$**,** where $\mathfrak{L} :\Gamma \to BCFS\left( {\k{\rm U} } \right)$. $BCFS\left( {\k{\rm U} } \right)$ is the family of all BCFSs of $\k{\rm U}$. It is presented as

$$ \begin{aligned} \mathfrak{L} = & \left( { \mathfrak{L} ,{ }\Gamma } \right) = \mathfrak{L} \left( {j_{\nu } } \right) = \left\{ {\left( {\imath_{\upsilon } , {\rm E}_{ \mathfrak{L} }^{ + } \left( {\imath_{\upsilon } } \right), {\rm E}_{ \mathfrak{L} }^{ - } \left( {\imath_{\upsilon } } \right)} \right)| \forall \imath_{\upsilon } \in \k{\rm U} ,\forall j_{\nu } \in \Gamma } \right\} \\ = & \left\{ {\left( {\imath_{\upsilon } , {\rm N}_{{\mathfrak{L}}}^{ + } \left( {\imath_{\upsilon } } \right) + \iota {\rm O}_{ \mathfrak{L} }^{ + } \left( {\imath_{\upsilon } } \right), {\rm N}_{ \mathfrak{L} }^{ - } \left( {\imath_{\upsilon } } \right) + \iota {\rm O}_{ \mathfrak{L} }^{ - } \left( {\imath_{\upsilon } } \right)} \right)| \forall \imath_{\upsilon } \in \k{\rm U} ,\forall j_{\nu } \in \Gamma } \right\} \\ \end{aligned} $$

### Definition 7

(Zadeh^20^): An IVFS $\mathfrak{L}$ over $\k{\rm U}$ of the shape $\mathfrak{L} = \left\{ {\left( {\imath , \left[ {{\rm E}_{ \mathfrak{L} }^{L} \left( \imath \right), {\rm E}_{ \mathfrak{L} }^{U} \left( \imath \right)} \right]} \right)|\imath \in \k{\rm U} } \right\}$ where ${\rm E}_{ \mathfrak{L} }^{L} \left( \imath \right):\k{\rm U} \to \left[ {0, 1} \right]$, and ${\rm E}_{ \mathfrak{L} }^{U} \left( \imath \right):\k{\rm U} \to \left[ {0, 1} \right]$ are the mappings. These mappings are called lower and upper degree membership TG.

### Definition 8

(Yang et al.^22^): Let $\k{\rm U}$ be the set of universe, the set of parameter $\Upsilon$ and $\Gamma \subseteq \Upsilon$, the pair $\left( { \mathfrak{L} ,{ }\Gamma } \right)$ is known as an IVFSS over $\k{\rm U}$, where $\mathfrak{L} :\Gamma \to IVFS\left( {\k{\rm U} } \right)$. $IVFS\left( {\k{\rm U} } \right)$ is the family of all IVFSs of $\k{\rm U}$. It is presented as

$$ \left( { \mathfrak{L} ,{ }\Gamma } \right) = \mathfrak{L} \left( {j_{\nu } } \right) = \left\{ {\left( {\imath_{\upsilon } , {\rm E}_{ \mathfrak{L} } \left( {\imath_{\upsilon } } \right)} \right)| \forall \imath_{\upsilon } \in \k{\rm U} ,\forall j_{\nu } \in \Gamma } \right\} = \left\{ {\left( {\imath_{\upsilon } , \begin{array}{*{20}c} {\left[ {{\rm N}_{ \mathfrak{L} }^{L} \left( {\imath_{\upsilon } } \right), {\rm O}_{ \mathfrak{L} }^{U} \left( {\imath_{\upsilon } } \right)} \right] } \\ \end{array} } \right)| \forall \imath_{\upsilon } \in \k{\rm U} ,\;\forall j_{\nu } \in \Gamma } \right\} $$

## Interval-valued bipolar complex fuzzy sets and interval-valued bipolar complex fuzzy soft sets

In this segment, we initiate the definition of interval-valued bipolar complex fuzzy set (IVBCFS), interval-valued bipolar complex fuzzy soft set (IVBCFSS) and their properties and examples.

### Interval-valued bipolar complex fuzzy set

Here, we demonstrate IVBCFS and their properties with examples.

#### Definition 9

Let $\k{\rm U}$ be the set of universe and $\c{D} \left( {\left[ {0, 1} \right]} \right)$ and $\c{D} \left( {\left[ { - 1, 0} \right]} \right)$ be the set of all closed subintervals of the interval $\left[ {0, 1} \right]$ and $\left[ { - 1, 0} \right]$, respectively, then an interval-valued BCFS (IVBCFS) ${\mathfrak{L}}$ is defined as

1 $$ \begin{aligned} {\mathfrak{L}} = & \left\{ {\left( {\imath , {\rm E}^{ + } \left( \imath \right), {\rm E}^{ - } \left( \imath \right)} \right)| \forall \imath \in \k{\rm U} } \right\} \\ = & \left\{ {\left( {\imath , \begin{array}{*{20}c} {\left[ {{\rm N}^{L + } \left( \imath \right), {\rm O}^{U + } \left( \imath \right)} \right] + \iota \left[ {{\rm N}^{L + } \left( \imath \right), {\rm O}^{U + } \left( \imath \right)} \right], } \\ {\left[ {{\rm N}^{L - } \left( \imath \right), {\rm O}^{U - } \left( \imath \right)} \right] + \iota \left[ {{\rm N}^{L - } \left( \imath \right), {\rm O}^{U - } \left( \imath \right)} \right]} \\ \end{array} } \right)| \forall \imath \in \k{\rm U} } \right\} \\ \end{aligned} $$

And ${\rm E}^{ + } \left( \imath \right):\k{\rm U} \to \c{D} \left( {\left[ {0, 1} \right]} \right) + \iota \c{D} \left( {\left[ {0, 1} \right]} \right)$ and ${\rm E}^{ - } \left( \imath \right):\k{\rm U} \to \c{D} \left( {\left[ { - 1, 0} \right]} \right) + \iota \c{D} \left( {\left[ { - 1, 0} \right]} \right)$ are the functions of the PTG and NTG, in that order. Also, ${\rm E}^{ + } \left( \imath \right) = \left[ {{\rm N}^{L + } \left( \imath \right), {\rm O}^{U + } \left( \imath \right)} \right] + \iota \left[ {{\rm N}^{L + } \left( \imath \right), {\rm O}^{U + } \left( \imath \right)} \right]$ denote the (lower and upper) PTG, and ${\rm E}^{ - } \left( \imath \right) = \left[ {{\rm N}^{L - } \left( \imath \right), {\rm O}^{U - } \left( \imath \right)} \right] + \iota \left[ {{\rm N}^{L - } \left( \imath \right), {\rm O}^{U - } \left( \imath \right)} \right]$ denote the (lower and upper) NTG, in that order, where ${\rm E}^{ + } \left( \imath \right) = \left[ {{\rm N}^{L + } \left( \imath \right), {\rm O}^{U + } \left( \imath \right)} \right] + \iota \left[ {{\rm N}^{L + } \left( \imath \right), {\rm O}^{U + } \left( \imath \right)} \right]:\k{\rm U} \to \c{D} \left( {\left[ {0, 1} \right]} \right) + \iota \c{D} \left( {\left[ {0, 1} \right]} \right)$ and ${\rm E}^{ - } \left( \imath \right) = \left[ {{\rm N}^{L - } \left( \imath \right), {\rm O}^{U - } \left( \imath \right)} \right] + \iota \left[ {{\rm N}^{L - } \left( \imath \right), {\rm O}^{U - } \left( \imath \right)} \right]:\k{\rm U} \to \c{D} \left( {\left[ { - 1, 0} \right]} \right) + \iota \c{D} \left( {\left[ { - 1, 0} \right]} \right)$. IVBCFS is represented as

$$ \mathfrak{L} = \left\{ {\begin{array}{*{20}c} {\left( {\imath_{1} , \left[ {{\rm N}^{L + } \left( {\imath_{1} } \right), {\rm O}^{U + } \left( {\imath_{1} } \right)} \right] + \iota \left[ {{\rm N}^{L + } \left( {\imath_{1} } \right), {\rm O}^{U + } \left( {\imath_{1} } \right)} \right], \left[ {{\rm N}^{L - } \left( {\imath_{1} } \right), {\rm O}^{U - } \left( {\imath_{1} } \right)} \right] + \iota \left[ {{\rm N}^{L - } \left( {\imath_{1} } \right), {\rm O}^{U - } \left( {\imath_{1} } \right)} \right]} \right),} \\ {\left( {\imath_{2} , \left[ {{\rm N}^{L + } \left( {\imath_{2} } \right), {\rm O}^{U + } \left( {\imath_{2} } \right)} \right] + \iota \left[ {{\rm N}^{L + } \left( {\imath_{2} } \right), {\rm O}^{U + } \left( {\imath_{2} } \right)} \right], \left[ {{\rm N}^{L - } \left( {\imath_{1} } \right), {\rm O}^{U - } \left( {\imath_{2} } \right)} \right] + \iota \left[ {{\rm N}^{L - } \left( {\imath_{2} } \right), {\rm O}^{U - } \left( {\imath_{2} } \right)} \right]} \right),} \\ {\left( {\imath_{3} , \left[ {{\rm N}^{L + } \left( {\imath_{3} } \right), {\rm O}^{U + } \left( {\imath_{3} } \right)} \right] + \iota \left[ {{\rm N}^{L + } \left( {\imath_{3} } \right), {\rm O}^{U + } \left( {\imath_{3} } \right)} \right], \left[ {{\rm N}^{L - } \left( {\imath_{3} } \right), {\rm O}^{U - } \left( {\imath_{3} } \right)} \right] + \iota \left[ {{\rm N}^{L - } \left( {\imath_{3} } \right), {\rm O}^{U - } \left( {\imath_{3} } \right)} \right]} \right),} \\ {\left( {\imath_{4} , \left[ {{\rm N}^{L + } \left( {\imath_{4} } \right), {\rm O}^{U + } \left( {\imath_{4} } \right)} \right] + \iota \left[ {{\rm N}^{L + } \left( {\imath_{4} } \right), {\rm O}^{U + } \left( {\imath_{4} } \right)} \right], \left[ {{\rm N}^{L - } \left( {\imath_{4} } \right), {\rm O}^{U - } \left( {\imath_{4} } \right)} \right] + \iota \left[ {{\rm N}^{L - } \left( {\imath_{4} } \right), {\rm O}^{U - } \left( {\imath_{4} } \right)} \right]} \right)} \\ \end{array} } \right\}, $$

#### Remark 1

For ${\mathbb{A}},{\mathbb{B}}\subseteq {\mathbb{R}}$, we denote and define as .

### Elementary operation on IVBCFSs

Here, we want to establish the operational laws like complement, union and intersection of the IVBCFSs with their examples.

#### Definition 10

The complement of IVBCFS $\mathfrak{L}$ is demonstrated and defined as

2 $$ \mathfrak{L}^{c} = \left\{ {\left( {\begin{array}{*{20}c} {\imath , \left[ {1 - {\rm O}^{U + } \left( \imath \right), 1 - {\rm N}^{L + } \left( \imath \right)} \right] + \iota \left[ {1 - {\rm O}^{U + } \left( \imath \right), 1 - {\rm N}^{L + } \left( \imath \right)} \right], } \\ {\left[ { - 1 - {\rm O}^{U - } \left( \imath \right), - 1 - {\rm N}^{L - } \left( \imath \right)} \right] + \iota \left[ { - 1 - {\rm O}^{U - } \left( \imath \right), - 1 - {\rm N}^{L - } \left( \imath \right)} \right]} \\ \end{array} } \right)\left| {\forall \imath \in \k{\rm U} } \right.} \right\} $$

#### Example 1

Consider an IVBCFS $\mathfrak{L}$ over $k{\rm{U}}$ defined in Example 1. The complement $\mathfrak{L}$ is designated as trails.

$${\mathfrak{L}}^{c}=\left\{\begin{array}{c}\left({\iota }_{1}, \left[0.4, 0.8\right]+\iota \left[0.6, 0.7\right], \left[-0.9,-0.7\right]+\iota \left[-0.8,-0.5\right]\right), \\ \left({\iota }_{2}, \left[0.5, 0.9\right]+\iota \left[0.7, 0.8\right], \left[-0.8, -0.4\right]+\iota \left[-0.7, -0.6\right]\right), \\ \left({\iota }_{3}, \left[0.6, 0.7\right]+\iota \left[0.7, 0.8\right], \left[-0.6, -0.5\right]+\iota \left[-0.4, -0.3\right]\right), \\ \left({\iota }_{4}, \left[0.5, 0.7\right]+\iota \left[0.8, 0.9\right], \left[-0.3, -0.2\right]+\iota \left[-0.8, -0.4\right]\right)\end{array}\right\}$$

#### Definition 11

The union and intersection of two IVBCFSs ${\mathfrak{L}}_{1}$ and ${\mathfrak{L}}_{2}$ over $\k{\rm U}$ is signified as

3 $${\mathfrak{L}}_{1}\cap {\mathfrak{L}}_{2}=\left\{\begin{array}{c}\left[min\left({\rm N}^{L+}\left({\iota }_{1}\right), {\rm N}^{L+}\left({\iota }_{2}\right)\right), min\left({\rm O}^{U+}\left({\iota }_{1}\right), {\rm O}^{U+}\left({\iota }_{2}\right)\right)\right]+\\ \iota \left[min\left({\rm N}^{L+}\left({\iota }_{1}\right), {\rm N}^{L+}\left({\iota }_{2}\right)\right), min\left({\rm O}^{U+}\left({\iota }_{1}\right), {\rm O}^{U+}\left({\iota }_{2}\right)\right)\right], \\ \left[max\left({\rm N}^{L-}\left({\iota }_{1}\right), {\rm N}^{L-}\left({\iota }_{2}\right)\right), max\left({\rm O}^{U-}\left({\iota }_{1}\right), {\rm O}^{U-}\left({\iota }_{2}\right)\right)\right]+\\ \iota \left[max\left({\rm N}^{L-}\left({\iota }_{1}\right), {\rm N}^{L-}\left({\iota }_{2}\right)\right), max\left({\rm O}^{U-}\left({\iota }_{1}\right), {\rm O}^{U-}\left({\iota }_{2}\right)\right)\right]\end{array}\right\}$$

4 $${\mathfrak{L}}_{1}\cup {\mathfrak{L}}_{2}=\left\{\begin{array}{c}\left[max\left({\rm N}^{L+}\left({\iota }_{1}\right), {\rm N}^{L+}\left({\iota }_{2}\right)\right), max\left({\rm O}^{U+}\left({\iota }_{1}\right), {\rm O}^{U+}\left({\iota }_{2}\right)\right)\right]+\\ \iota \left[max\left({\rm N}^{L+}\left({\iota }_{1}\right), {\rm N}^{L+}\left({\iota }_{2}\right)\right), max\left({\rm O}^{U+}\left({\iota }_{1}\right), {\rm O}^{U+}\left({\iota }_{2}\right)\right)\right], \\ \left[min\left({\rm N}^{L-}\left({\iota }_{1}\right), {\rm N}^{L-}\left({\iota }_{2}\right)\right), min\left({\rm O}^{U-}\left({\iota }_{1}\right), {\rm O}^{U-}\left({\iota }_{2}\right)\right)\right]+\\ \iota \left[min\left({\rm N}^{L-}\left({\iota }_{1}\right), {\rm N}^{L-}\left({\iota }_{2}\right)\right), min\left({\rm O}^{U-}\left({\iota }_{1}\right), {\rm O}^{U-}\left({\iota }_{2}\right)\right)\right]\end{array}\right\}$$

#### Example 2

Consider two IVBCFSs ${\mathfrak{L}}_{1}$ and ${\mathfrak{L}}_{2}$ over $\k{\rm U}$ in Example 2. The union and intersection of ${\mathfrak{L}}_{1}$ and ${\mathfrak{L}}_{2}$ are as follows:

$${\mathfrak{L}}_{1}=\left\{\begin{array}{c}\left({\iota }_{1}, \left[0.3, 0.7\right]+\iota \left[0.4, 0.8\right], \left[-0.8,-0.7\right]+\iota \left[-0.9,-0.8\right]\right), \\ \left({\iota }_{2}, \left[0.8, 0.9\right]+\iota \left[0.6, 0.7\right], \left[-0.5, -0.3\right]+\iota \left[-0.4, -0.1\right]\right), \\ \left({\iota }_{3}, \left[0.5, 0.7\right]+\iota \left[0.8, 0.9\right], \left[-0.8, -0.7\right]+\iota \left[-0.6, -0.5\right]\right), \\ \left({\iota }_{4}, \left[0.3, 0.4\right]+\iota \left[0.5, 0.6\right], \left[-0.4, -0.3\right]+\iota \left[-0.3, -0.2\right]\right)\end{array}\right\}$$

$${\mathfrak{L}}_{2}=\left\{\begin{array}{c}\left({\iota }_{1}, \left[0.1, 0.5\right]+\iota \left[0.2, 0.3\right], \left[-0.4,-0.2\right]+\iota \left[-0.6,-0.3\right]\right), \\ \left({\iota }_{2}, \left[0.2, 0.4\right]+\iota \left[0.3, 0.4\right], \left[-0.3, -0.1\right]+\iota \left[-0.5, -0.2\right]\right), \\ \left({\iota }_{3}, \left[0.4, 0.6\right]+\iota \left[0.7, 0.8\right], \left[-0.9, -0.8\right]+\iota \left[-0.7, -0.6\right]\right), \\ \left({\iota }_{4}, \left[0.1, 0.4\right]+\iota \left[0.2, 0.3\right], \left[-0.6, -0.5\right]+\iota \left[-0.9, -0.8\right]\right)\end{array}\right\}$$

Then their union and intersection defined as

$${\mathfrak{L}}_{1}\cap {\mathfrak{L}}_{2}=\left\{\begin{array}{c}\left({\iota }_{1}, \left[0.1, 0.5\right]+\iota \left[0.2, 0.3\right], \left[-0.4,-0.2\right]+\iota \left[-0.6,-0.3\right]\right), \\ \left({\iota }_{2}, \left[0.2, 0.4\right]+\iota \left[0.3, 0.4\right], \left[-0.3, -0.1\right]+\iota \left[-0.4, -0.1\right]\right),\\ \left({\iota }_{3}, \left[0.4, 0.6\right]+\iota \left[0.7, 0.8\right], \left[-0.8, -0.7\right]+\iota \left[-0.6, -0.5\right]\right),\\ \left({\iota }_{4}, \left[0.1, 0.4\right]+\iota \left[0.2, 0.3\right], \left[-0.4, -0.3\right]+\iota \left[-0.3, -0.2\right]\right)\end{array}\right\}$$

$${\mathfrak{L}}_{1}\cup {\mathfrak{L}}_{2}=\left\{\begin{array}{c}\left({\iota }_{1}, \left[0.3, 0.7\right]+\iota \left[0.4, 0.8\right], \left[-0.8,-0.7\right]+\iota \left[-0.9,-0.8\right]\right), \\ \left({\iota }_{2}, \left[0.8, 0.9\right]+\iota \left[0.6, 0.7\right], \left[-0.5, -0.3\right]+\iota \left[-0.5, -0.2\right]\right),\\ \left({\iota }_{3}, \left[0.5, 0.7\right]+\iota \left[0.8, 0.9\right], \left[-0.9, -0.8\right]+\iota \left[-0.7, -0.6\right]\right),\\ \left({\iota }_{4}, \left[0.3, 0.4\right]+\iota \left[0.5, 0.6\right], \left[-0.6, -0.5\right]+\iota \left[-0.9, -0.8\right]\right)\end{array}\right\}$$

### Interval-valued Bipolar complex fuzzy soft set

Here, we demonstrate IVBCFSS and their properties with examples.

#### Definition 12

Suppose that the fixed set $\k{\rm U}$, the set of parameter $\Upsilon$ and $\Gamma \subseteq \Upsilon$, the pair $\left( { \mathfrak{L} ,{ }\Gamma } \right)$ is called an IVBCFSS over $\k{\rm U}$**,** where $\mathfrak{L} :\Gamma \to IVBCFS\left( {\k{\rm U} } \right)$. $IVBCFS\left( {\k{\rm U} } \right)$ is the family of all IVBCFSs of $\k{\rm U}$. It is presented as

5 $$ \begin{aligned} \left( { \mathfrak{L} ,{ }\Gamma } \right) = & \mathfrak{L} \left( {j_{\nu } } \right) = \left\{ {\left( {\imath_{\upsilon } , {\rm E}_{ \mathfrak{L} }^{ + } \left( {\imath_{\upsilon } } \right), {\rm E}_{ \mathfrak{L} }^{ - } \left( {\imath_{\upsilon } } \right)} \right)| \forall \imath_{\upsilon } \in \k{\rm U} ,\forall j_{\nu } \in \Gamma } \right\} \\ = & \left\{ {\left( {\imath_{\upsilon } , \begin{array}{*{20}c} {\left[ {{\rm N}_{ \mathfrak{L} }^{L + } \left( {\imath_{\upsilon } } \right), {\rm O}_{ \mathfrak{L} }^{U + } \left( {\imath_{\upsilon } } \right)} \right] + \iota \left[ {{\rm N}_{ \mathfrak{L} }^{L + } \left( {\imath_{\upsilon } } \right), {\rm O}_{ \mathfrak{L} }^{U + } \left( {\imath_{\upsilon } } \right)} \right], } \\ {\left[ {{\rm N}_{ \mathfrak{L} }^{L - } \left( {\imath_{\upsilon } } \right), {\rm O}_{ \mathfrak{L} }^{U - } \left( {\imath_{\upsilon } } \right)} \right] + \iota \left[ {{\rm N}_{ \mathfrak{L} }^{L - } \left( {\imath_{\upsilon } } \right), {\rm O}_{ \mathfrak{L} }^{U - } \left( {\imath_{\upsilon } } \right)} \right]} \\ \end{array} } \right)| \forall \imath_{\upsilon } \in \k{\rm U} ,\forall j_{\nu } \in \Gamma } \right\} \\ \end{aligned} $$

#### Remark 2

For a set $\k{\rm U} = \left\{ {\imath_{1} , \imath_{2} , \imath_{3} , \ldots , \imath_{{{\tilde{r}} }} } \right\}$ and $\Gamma = \left\{ {j_{1} , j_{2} , j_{3} , \ldots , j_{{{\tilde{s}} }} } \right\} \subseteq \Upsilon$, the tabular demonstration of IVBCFSS $\left(\mathfrak{L},\Gamma \right)$ is indicate in Table 1.

**Table 1:** The tabular demonstration of IVBCFSS.

$\left(\mathfrak{L},\Gamma \right)$	${j}_{1}$	${j}_{2}$	…	${j}_{{\tilde{s}}}$
${\iota }_{1}$	$$\left(\begin{array}{c}\left[{\rm N}_{{\mathfrak{L}}_{11}}^{L+}, {\rm O}_{{\mathfrak{L}}_{11}}^{U+}\right]+\iota \left[{\rm N}_{{\mathfrak{L}}_{11}}^{L+}, {\rm O}_{{\mathfrak{L}}_{11}}^{U+}\right], \\ \left[{\rm N}_{{\mathfrak{L}}_{11}}^{L-}, {\rm O}_{{\mathfrak{L}}_{11}}^{U-}\right]+\iota \left[{\rm N}_{{\mathfrak{L}}_{11}}^{L-}, {\rm O}_{{\mathfrak{L}}_{11}}^{U-}\right]\end{array}\right)$$	$$\left(\begin{array}{c}\left[{\rm N}_{{\mathfrak{L}}_{12}}^{L+}, {\rm O}_{{\mathfrak{L}}_{12}}^{U+}\right]+\iota \left[{\rm N}_{{\mathfrak{L}}_{12}}^{L+}, {\rm O}_{{\mathfrak{L}}_{12}}^{U+}\right], \\ \left[{\rm N}_{{\mathfrak{L}}_{12}}^{L-}, {\rm O}_{{\mathfrak{L}}_{12}}^{U-}\right]+\iota \left[{\rm N}_{{\mathfrak{L}}_{12}}^{L-}, {\rm O}_{{\mathfrak{L}}_{12}}^{U-}\right]\end{array}\right)$$	…	$$\left(\begin{array}{c}\left[{\rm N}_{{\mathfrak{L}}_{1{{\tilde{s}}}}}^{L+}, {\rm O}_{{\mathfrak{L}}_{1{{\tilde{s}}}}}^{U+}\right]+\iota \left[{\rm N}_{{\mathfrak{L}}_{1{{\tilde{s}}}}}^{L+}, {\rm O}_{{\mathfrak{L}}_{1{{\tilde{s}}}}}^{U+}\right], \\ \left[{\rm N}_{{\mathfrak{L}}_{1{{\tilde{s}}}}}^{L-}, {\rm O}_{{\mathfrak{L}}_{1{{\tilde{s}}}}}^{U-}\right]+\iota \left[{\rm N}_{{\mathfrak{L}}_{1{{\tilde{s}}}}}^{L-}, {\rm O}_{{\mathfrak{L}}_{1{{\tilde{s}}}}}^{U-}\right]\end{array}\right)$$
${\iota }_{2}$	$$\left(\begin{array}{c}\left[{\rm N}_{{\mathfrak{L}}_{21}}^{L+}, {\rm O}_{{\mathfrak{L}}_{21}}^{U+}\right]+\iota \left[{\rm N}_{{\mathfrak{L}}_{21}}^{L+}, {\rm O}_{{\mathfrak{L}}_{21}}^{U+}\right], \\ \left[{\rm N}_{{\mathfrak{L}}_{21}}^{L-}, {\rm O}_{{\mathfrak{L}}_{21}}^{U-}\right]+\iota \left[{\rm N}_{{\mathfrak{L}}_{21}}^{L-}, {\rm O}_{{\mathfrak{L}}_{21}}^{U-}\right]\end{array}\right)$$	$$\left(\begin{array}{c}\left[{\rm N}_{{\mathfrak{L}}_{22}}^{L+}, {\rm O}_{{\mathfrak{L}}_{22}}^{U+}\right]+\iota \left[{\rm N}_{{\mathfrak{L}}_{22}}^{L+}, {\rm O}_{{\mathfrak{L}}_{22}}^{U+}\right], \\ \left[{\rm N}_{{\mathfrak{L}}_{22}}^{L-}, {\rm O}_{{\mathfrak{L}}_{22}}^{U-}\right]+\iota \left[{\rm N}_{{\mathfrak{L}}_{22}}^{L-}, {\rm O}_{{\mathfrak{L}}_{22}}^{U-}\right]\end{array}\right)$$	…	$$\left(\begin{array}{c}\left[{\rm N}_{{\mathfrak{L}}_{2{{\tilde{s}}}}}^{L+}, {\rm O}_{{\mathfrak{L}}_{2{{\tilde{s}}}}}^{U+}\right]+\iota \left[{\rm N}_{{\mathfrak{L}}_{2{{\tilde{s}}}}}^{L+}, {\rm O}_{{\mathfrak{L}}_{2{{\tilde{s}}}}}^{U+}\right], \\ \left[{\rm N}_{{\mathfrak{L}}_{2{{\tilde{s}}}}}^{L-}, {\rm O}_{{\mathfrak{L}}_{2{{\tilde{s}}}}}^{U-}\right]+\iota \left[{\rm N}_{{\mathfrak{L}}_{2{{\tilde{s}}}}}^{L-}, {\rm O}_{{\mathfrak{L}}_{2{{\tilde{s}}}}}^{U-}\right]\end{array}\right)$$
${\iota }_{3}$	$$\left(\begin{array}{c}\left[{\rm N}_{{\mathfrak{L}}_{31}}^{L+}, {\rm O}_{{\mathfrak{L}}_{31}}^{U+}\right]+\iota \left[{\rm N}_{{\mathfrak{L}}_{31}}^{L+}, {\rm O}_{{\mathfrak{L}}_{31}}^{U+}\right], \\ \left[{\rm N}_{{\mathfrak{L}}_{31}}^{L-}, {\rm O}_{{\mathfrak{L}}_{31}}^{U-}\right]+\iota \left[{\rm N}_{{\mathfrak{L}}_{31}}^{L-}, {\rm O}_{{\mathfrak{L}}_{31}}^{U-}\right]\end{array}\right)$$	$$\left(\begin{array}{c}\left[{\rm N}_{{\mathfrak{L}}_{32}}^{L+}, {\rm O}_{{\mathfrak{L}}_{32}}^{U+}\right]+\iota \left[{\rm N}_{{\mathfrak{L}}_{32}}^{L+}, {\rm O}_{{\mathfrak{L}}_{32}}^{U+}\right], \\ \left[{\rm N}_{{\mathfrak{L}}_{32}}^{L-}, {\rm O}_{{\mathfrak{L}}_{32}}^{U-}\right]+\iota \left[{\rm N}_{{\mathfrak{L}}_{32}}^{L-}, {\rm O}_{{\mathfrak{L}}_{32}}^{U-}\right]\end{array}\right)$$	…	$$\left(\begin{array}{c}\left[{\rm N}_{{\mathfrak{L}}_{3{{\tilde{s}}}}}^{L+}, {\rm O}_{{\mathfrak{L}}_{3{{\tilde{s}}}}}^{U+}\right]+\iota \left[{\rm N}_{{\mathfrak{L}}_{3{{\tilde{s}}}}}^{L+}, {\rm O}_{{\mathfrak{L}}_{3{{\tilde{s}}}}}^{U+}\right], \\ \left[{\rm N}_{{\mathfrak{L}}_{3{{\tilde{s}}}}}^{L-}, {\rm O}_{{\mathfrak{L}}_{3{{\tilde{s}}}}}^{U-}\right]+\iota \left[{\rm N}_{{\mathfrak{L}}_{3{{\tilde{s}}}}}^{L-}, {\rm O}_{{\mathfrak{L}}_{3{{\tilde{s}}}}}^{U-}\right]\end{array}\right)$$
…	…	…	…	…
${\iota }_{{\tilde{r}}}$	$$\left(\begin{array}{c}\left[{\rm N}_{{\mathfrak{L}}_{{{\tilde{r}}}1}}^{L+}, {\rm O}_{{\mathfrak{L}}_{{{\tilde{r}}}1}}^{U+}\right]+\iota \left[{\rm N}_{{\mathfrak{L}}_{{{\tilde{r}}}1}}^{L+}, {\rm O}_{{\mathfrak{L}}_{{{\tilde{r}}}1}}^{U+}\right], \\ \left[{\rm N}_{{\mathfrak{L}}_{{{\tilde{r}}}1}}^{L-}, {\rm O}_{{\mathfrak{L}}_{{{\tilde{r}}}1}}^{U-}\right]+\iota \left[{\rm N}_{{\mathfrak{L}}_{{{\tilde{r}}}1}}^{L-}, {\rm O}_{{\mathfrak{L}}_{{{\tilde{r}}}1}}^{U-}\right]\end{array}\right)$$	$$\left(\begin{array}{c}\left[{\rm N}_{{\mathfrak{L}}_{{{\tilde{r}}}2}}^{L+}, {\rm O}_{{\mathfrak{L}}_{{{\tilde{r}}}2}}^{U+}\right]+\iota \left[{\rm N}_{{\mathfrak{L}}_{{{\tilde{r}}}2}}^{L+}, {\rm O}_{{\mathfrak{L}}_{{{\tilde{r}}}2}}^{U+}\right], \\ \left[{\rm N}_{{\mathfrak{L}}_{{{\tilde{r}}}2}}^{L-}, {\rm O}_{{\mathfrak{L}}_{{{\tilde{r}}}2}}^{U-}\right]+\iota \left[{\rm N}_{{\mathfrak{L}}_{{{\tilde{r}}}2}}^{L-}, {\rm O}_{{\mathfrak{L}}_{{{\tilde{r}}}2}}^{U-}\right]\end{array}\right)$$	…	$$\left(\begin{array}{c}\left[{\rm N}_{{\mathfrak{L}}_{{{\tilde{r}}}{{\tilde{s}}}}}^{L+}, {\rm O}_{{\mathfrak{L}}_{{{\tilde{r}}}{{\tilde{s}}}}}^{U+}\right]+\iota \left[{\rm N}_{{\mathfrak{L}}_{{{\tilde{r}}}{{\tilde{s}}}}}^{L+}, {\rm O}_{{\mathfrak{L}}_{{{\tilde{r}}}{{\tilde{s}}}}}^{U+}\right], \\ \left[{\rm N}_{{\mathfrak{L}}_{{{\tilde{r}}}{{\tilde{s}}}}}^{L-}, {\rm O}_{{\mathfrak{L}}_{{{\tilde{r}}}{{\tilde{s}}}}}^{U-}\right]+\iota \left[{\rm N}_{{\mathfrak{L}}_{{{\tilde{r}}}{{\tilde{s}}}}}^{L-}, {\rm O}_{{\mathfrak{L}}_{{{\tilde{r}}}{{\tilde{s}}}}}^{U-}\right]\end{array}\right)$$

#### Example 3

Suppose that $\k{\rm U} = \left\{ {\imath_{1} , \imath_{2} , \imath_{3} , \imath_{4} } \right\}$ is the set of four under consideration football players and $\Gamma =\left\{{j}_{1}=technique, {j}_{2}=Fitness, {j}_{3}=Mindset\right\}\subseteq \Upsilon$ is the set of parameters, then the IVBCFSS is demonstrated as follows

$$\left(\mathfrak{L},\Gamma \right)=\left\{\begin{array}{c}\mathfrak{L}\left({j}_{1}\right)=\left\{\begin{array}{c}\left({\iota }_{1}, \left[0.2, 0.6\right]+\iota \left[0.3, 0.4\right], \left[-0.3,-0.1\right]+\iota \left[-0.5,-0.2\right]\right), \\ \left({\iota }_{2}, \left[0.1, 0.5\right]+\iota \left[0.2, 0.3\right], \left[-0.6, -0.2\right]+\iota \left[-0.4, -0.3\right]\right), \\ \left({\iota }_{3}, \left[0.3, 0.4\right]+\iota \left[0.2, 0.3\right], \left[-0.5, -0.4\right]+\iota \left[-0.7, -0.6\right]\right), \\ \left({\iota }_{4}, \left[0.3, 0.5\right]+\iota \left[0.1, 0.2\right], \left[-0.8, -0.7\right]+\iota \left[-0.6, -0.2\right]\right)\end{array}\right\}\\ \mathfrak{L}\left({j}_{2}\right)=\left\{\begin{array}{c}\left({\iota }_{1}, \left[0.1, 0.5\right]+\iota \left[0.2, 0.3\right], \left[-0.4,-0.2\right]+\iota \left[-0.6,-0.3\right]\right), \\ \left({\iota }_{2}, \left[0.2, 0.4\right]+\iota \left[0.3, 0.4\right], \left[-0.3, -0.1\right]+\iota \left[-0.5, -0.2\right]\right), \\ \left({\iota }_{3}, \left[0.4, 0.6\right]+\iota \left[0.7, 0.8\right], \left[-0.9, -0.8\right]+\iota \left[-0.7, -0.6\right]\right), \\ \left({\iota }_{4}, \left[0.1, 0.4\right]+\iota \left[0.2, 0.3\right], \left[-0.6, -0.5\right]+\iota \left[-0.9, -0.8\right]\right)\end{array}\right\}\\ \mathfrak{L}\left({j}_{3}\right)=\left\{\begin{array}{c}\left({\iota }_{1}, \left[0.3, 0.7\right]+\iota \left[0.4, 0.8\right], \left[-0.8,-0.7\right]+\iota \left[-0.9,-0.8\right]\right), \\ \left({\iota }_{2}, \left[0.8, 0.9\right]+\iota \left[0.6, 0.7\right], \left[-0.5, -0.3\right]+\iota \left[-0.4, -0.1\right]\right), \\ \left({\iota }_{3}, \left[0.5, 0.7\right]+\iota \left[0.8, 0.9\right], \left[-0.8, -0.7\right]+\iota \left[-0.6, -0.5\right]\right), \\ \left({\iota }_{4}, \left[0.3, 0.4\right]+\iota \left[0.5, 0.6\right], \left[-0.4, -0.3\right]+\iota \left[-0.3, -0.2\right]\right)\end{array}\right\}\end{array}\right\}$$

The tabular explanation of IVBCFSS demonstrated in example 1 is indicated in Table 2.

**Table 2:** The tabular demonstration of IVBCFSS described in example 1.

$\left(\mathfrak{L},\Gamma \right)$	${j}_{1}$	${j}_{2}$	${j}_{3}$
${\iota }_{1}$	$$\left(\begin{array}{c}\left[0.2, 0.6\right]+\iota \left[0.3, 0.4\right], \\ \left[-0.3,-0.1\right]+\iota \left[-0.5,-0.2\right]\end{array}\right)$$	$$\left(\begin{array}{c}\left[0.1, 0.5\right]+\iota \left[0.2, 0.3\right], \\ \left[-0.4,-0.2\right]+\iota \left[-0.6,-0.3\right]\end{array}\right)$$	$$\left(\begin{array}{c}\left[0.3, 0.7\right]+\iota \left[0.4, 0.8\right], \\ \left[-0.8,-0.7\right]+\iota \left[-0.9,-0.8\right]\end{array}\right)$$
${\iota }_{2}$	$$\left(\begin{array}{c}\left[0.1, 0.5\right]+\iota \left[0.2, 0.3\right], \\ \left[-0.6, -0.2\right]+\iota \left[-0.4, -0.3\right]\end{array}\right)$$	$$\left(\begin{array}{c}\left[0.2, 0.4\right]+\iota \left[0.3, 0.4\right], \\ \left[-0.3, -0.1\right]+\iota \left[-0.5, -0.2\right]\end{array}\right)$$	$$\left(\begin{array}{c}\left[0.8, 0.9\right]+\iota \left[0.6, 0.7\right], \\ \left[-0.5, -0.3\right]+\iota \left[-0.4, -0.1\right]\end{array}\right)$$
${\iota }_{3}$	$$\left(\begin{array}{c}\left[0.3, 0.4\right]+\iota \left[0.2, 0.3\right], \\ \left[-0.5, -0.4\right]+\iota \left[-0.7, -0.6\right]\end{array}\right)$$	$$\left(\begin{array}{c}\left[0.4, 0.6\right]+\iota \left[0.7, 0.8\right], \\ \left[-0.9, -0.8\right]+\iota \left[-0.7, -0.6\right]\end{array}\right)$$	$$\left(\begin{array}{c}\left[0.5, 0.7\right]+\iota \left[0.8, 0.9\right], \\ \left[-0.8, -0.7\right]+\iota \left[-0.6, -0.5\right]\end{array}\right)$$
${\iota }_{4}$	$$\left(\begin{array}{c}\left[0.3, 0.5\right]+\iota \left[0.1, 0.2\right], \\ \left[-0.8, -0.7\right]+\iota \left[-0.6, -0.2\right]\end{array}\right)$$	$$\left(\begin{array}{c}\left[0.1, 0.4\right]+\iota \left[0.2, 0.3\right], \\ \left[-0.6, -0.5\right]+\iota \left[-0.9, -0.8\right]\end{array}\right)$$	$$\left(\begin{array}{c}\left[0.3, 0.4\right]+\iota \left[0.5, 0.6\right], \\ \left[-0.4, -0.3\right]+\iota \left[-0.3, -0.2\right]\end{array}\right)$$

#### Definition 13

An IVBCFSS $\left(\mathfrak{L},\Gamma \right)$ is called null IVBCFSS if $\forall {j}_{\nu }\in \Gamma , \mathfrak{L}\left({j}_{\nu }\right)=\emptyset$ and chosen by empty set $\emptyset$.

#### Definition 14

An IVBCFSS $\left(\mathfrak{L},\Gamma \right)$ is called absolute IVBCFSS if $\forall j_{\nu } \in \Gamma , \mathfrak{L} \left( {j_{\nu } } \right) = IVBCFS\left( {\k{\rm U} } \right)$.

#### Definition 15

Assume that $\left(\mathfrak{L},\Gamma \right)$ and $\left(\pounds , \text{\yen} \right)$ are two IVBCFSSs over $\k{\rm U}$, then $\left(\mathfrak{L},\Gamma \right)$ is is known as IVBCFS subset of $\left(\pounds , \text{\yen} \right)$ if

1. $$\Gamma \subseteq \text{\yen} $$,
2. $\forall j\in \Gamma$, $\mathfrak{L}\left(j\right)$ is IVBCF subset of $\pounds \left(j\right)$, i.e. for real part
  $$ {\rm N}_{{\mathfrak{L}}}^{L + } \left( {\imath_{\upsilon } } \right) \le {\rm N}_{{\text{\pounds}}}^{L + } \left( {\imath_{\upsilon } } \right),{ }{\rm O}_{{\mathfrak{L}}}^{U + } \left( {\imath_{\upsilon } } \right) \le {\rm O}_{{\text{\pounds}}}^{U + } \left( {\imath_{\upsilon } } \right),{ }{\rm N}_{{\mathfrak{L}}}^{L - } \left( {\imath_{\upsilon } } \right) \ge {\rm N}_{{\text{\pounds}}}^{L - } \left( {\imath_{\upsilon } } \right),{ }{\rm O}_{{\mathfrak{L}}}^{U - } \left( {\imath_{\upsilon } } \right) \ge {\rm O}_{{\text{\pounds}}}^{U - } \left( {\imath_{\upsilon } } \right), $$
  And for imaginary part
  $$ {\rm N}_{{\mathfrak{L}}}^{L + } \left( {\imath_{\upsilon } } \right) \le {\rm N}_{\pounds}^{L + } \left( {\imath_{\upsilon } } \right), {\rm O}_{{\mathfrak{L}}}^{U + } \left( {\imath_{\upsilon } } \right) \le {\rm O}_{\pounds}^{U + } \left( {\imath_{\upsilon } } \right), , {\rm N}_{{\mathfrak{L}}}^{L - } \left( {\imath_{\upsilon } } \right) \ge {\rm N}_{\pounds}^{L - } \left( {\imath_{\upsilon } } \right), {\rm O}_{{\mathfrak{L}}}^{U - } \left( {\imath_{\upsilon } } \right) \ge {\rm O}_{\pounds}^{U - } \left( {\imath_{\upsilon } } \right)\forall \imath_{\upsilon } \in \k{\rm U} . $$

#### Definition 16

Assume that $\left( {{\mathfrak{L}}, \Gamma } \right)$ and $\left( {\pounds, {\text{\yen}} } \right)$ are two IVBCFSSs over $\k{\rm U}$, then $\left( {{\mathfrak{L}}, \Gamma } \right)$ and $\left( {\pounds,{\text{\yen}} } \right)$ are assumed to be IVBCFS equal sets if $\left( {{\mathfrak{L}}, \Gamma } \right) \subseteq \left( {\pounds, {\text{\yen}} } \right)$ and $\left( {\pounds, {\text{\yen}} } \right) \subseteq \left( {{\mathfrak{L}}, \Gamma } \right)$.

### Elementary operation on IVBCFSSs

Here, we will initiate some elementary operations for IVBCFSSs like complement, union, extended union, intersection, extended intersection and associated properties.

#### Definition 17

The complement of an IVBCFSS $\left(\mathfrak{L},\Gamma \right)$ is indicated and described as

6 $${\left(\mathfrak{L},\Gamma \right)}^{c}=\left\{\left({\iota }_{\upsilon }, \begin{array}{c}\left[1-{\rm O}_{\mathfrak{L}}^{U+}\left({\iota }_{\upsilon }\right), {1-{\rm N}}_{\mathfrak{L}}^{L+}\left({\iota }_{\upsilon }\right)\right]+\iota \left[1-{\rm O}_{\mathfrak{L}}^{U+}\left({\iota }_{\upsilon }\right), {1-{\rm N}}_{\mathfrak{L}}^{L+}\left({\iota }_{\upsilon }\right)\right], \\ \left[-1-{\rm O}_{\mathfrak{L}}^{U-}\left({\iota }_{\upsilon }\right), {-1-{\rm N}}_{\mathfrak{L}}^{L-}\left({\iota }_{\upsilon }\right)\right]+\iota \left[-1-{\rm O}_{\mathfrak{L}}^{U-}\left({\iota }_{\upsilon }\right), {-1-{\rm N}}_{\mathfrak{L}}^{L-}\left({\iota }_{\upsilon }\right)\right]\end{array}\right)\right\}$$

#### Example 4

Consider an IVBCFSS $\left(\mathfrak{L},\Gamma \right)$ over $\k{\rm U}$ obtainable in Example 1. The complement of $\left(\mathfrak{L},\Gamma \right)$ is demonstrated as follows

$${\left(\mathfrak{L},\Gamma \right)}^{c}=\left\{\begin{array}{c}\mathfrak{L}\left({j}_{1}\right)=\left\{\begin{array}{c}\left({\iota }_{1}, \left[0.4, 0.8\right]+\iota \left[0.6, 0.7\right], \left[-0.9,-0.7\right]+\iota \left[-0.8,-0.5\right]\right), \\ \left({\iota }_{2}, \left[0.5, 0.9\right]+\iota \left[0.7, 0.8\right], \left[-0.8, -0.4\right]+\iota \left[-0.7, -0.6\right]\right), \\ \left({\iota }_{3}, \left[0.6, 0.7\right]+\iota \left[0.7, 0.8\right], \left[-0.6, -0.5\right]+\iota \left[-0.4, -0.3\right]\right), \\ \left({\iota }_{4}, \left[0.5, 0.7\right]+\iota \left[0.8, 0.9\right], \left[-0.3, -0.2\right]+\iota \left[-0.8, -0.4\right]\right)\end{array}\right\}\\ \mathfrak{L}\left({j}_{2}\right)=\left\{\begin{array}{c}\left({\iota }_{1}, \left[0.5, 0.9\right]+\iota \left[0.7, 0.8\right], \left[-0.8,-0.6\right]+\iota \left[-0.7,-0.4\right]\right), \\ \left({\iota }_{2}, \left[0.6, 0.8\right]+\iota \left[0.6, 0.7\right], \left[-0.9, -0.7\right]+\iota \left[-0.8, -0.5\right]\right), \\ \left({\iota }_{3}, \left[0.4, 0.6\right]+\iota \left[0.2, 0.3\right], \left[-0.2, -0.1\right]+\iota \left[-0.4, -0.3\right]\right), \\ \left({\iota }_{4}, \left[0.6, 0.9\right]+\iota \left[0.7, 0.8\right], \left[-0.5, -0.4\right]+\iota \left[-0.2, -0.1\right]\right)\end{array}\right\}\\ \mathfrak{L}\left({j}_{3}\right)=\left\{\begin{array}{c}\left({\iota }_{1}, \left[0.3, 0.7\right]+\iota \left[0.2, 0.6\right], \left[-0.3,-0.2\right]+\iota \left[-0.2,-0.1\right]\right), \\ \left({\iota }_{2}, \left[0.1, 0.2\right]+\iota \left[0.3, 0.4\right], \left[-0.7, -0.5\right]+\iota \left[-0.9, -0.6\right]\right), \\ \left({\iota }_{3}, \left[0.3, 0.5\right]+\iota \left[0.1, 0.2\right], \left[-0.3, -0.2\right]+\iota \left[-0.5, -0.4\right]\right), \\ \left({\iota }_{4}, \left[0.6, 0.7\right]+\iota \left[0.4, 0.5\right], \left[-0.7, -0.6\right]+\iota \left[-0.8, -0.7\right]\right)\end{array}\right\}\end{array}\right\}$$

#### Definition 18

The restricted intersection of two IVBCFSSs $\left( {{\mathfrak{L}}, \Gamma } \right)$ and $\left( {\pounds, {\text{\yen}} } \right)$ over $\k{\rm U}$ is an IVBCFSS $\left( {{\text{\AA}}_{R} , {\text{T}}} \right)$, where ${\text{T}} = \Gamma \cap {\text{\yen}} \ne \emptyset$ and ${\text{\AA}}_{R} :{\text{T}} \to IVBCFS\left( {\k{\rm U} } \right)$ considered as ${\text{\AA}}_{R} \left( j \right) = {\mathfrak{L}}\left( j \right) \cap \pounds\left( j \right)$ and indicated by $\left( {{\text{\AA}}_{R} , {\text{T}}} \right) = \left( {{\mathfrak{L}}, \Gamma } \right) \cap_{R} \left( {\pounds, {\text{\yen}} } \right)$.

#### Example 5

Suppose that $\k{\rm U} = \left\{ {\imath_{1} , \imath_{2} , \imath_{3} , \imath_{4} } \right\}$ is the set of dissimilar sights and $\Upsilon = \left\{ {j_{1} = open\; atmosphere, j_{2} = green \;surroundings, j_{3} = beautiful \;view \;of\;sun, j_{4} = trend\; of\; the \;snowfall} \right\}$ is the set of parameters, $\Gamma = \left\{ {j_{1} , j_{3} , j_{4} } \right\} \subset \Upsilon$ and ${\text{\yen}} = \left\{ {j_{1} , j_{2} , j_{4} } \right\} \subset \Upsilon$ then the two IVBCFSSs are specified as

$$\left(\mathfrak{L},\Gamma \right)=\left\{\begin{array}{c}\mathfrak{L}\left({j}_{1}\right)=\left\{\begin{array}{c}\left({\iota }_{1}, \left[0.2, 0.6\right]+\iota \left[0.3, 0.4\right], \left[-0.3,-0.1\right]+\iota \left[-0.5,-0.2\right]\right), \\ \left({\iota }_{2}, \left[0.1, 0.5\right]+\iota \left[0.2, 0.3\right], \left[-0.6, -0.2\right]+\iota \left[-0.4, -0.3\right]\right), \\ \left({\iota }_{3}, \left[0.3, 0.4\right]+\iota \left[0.2, 0.3\right], \left[-0.5, -0.4\right]+\iota \left[-0.7, -0.6\right]\right), \\ \left({\iota }_{4}, \left[0.3, 0.5\right]+\iota \left[0.1, 0.2\right], \left[-0.8, -0.7\right]+\iota \left[-0.6, -0.2\right]\right)\end{array}\right\}\\ \mathfrak{L}\left({j}_{3}\right)=\left\{\begin{array}{c}\left({\iota }_{1}, \left[0.1, 0.5\right]+\iota \left[0.2, 0.3\right], \left[-0.4,-0.2\right]+\iota \left[-0.6,-0.3\right]\right), \\ \left({\iota }_{2}, \left[0.2, 0.4\right]+\iota \left[0.3, 0.4\right], \left[-0.3, -0.1\right]+\iota \left[-0.5, -0.2\right]\right), \\ \left({\iota }_{3}, \left[0.4, 0.6\right]+\iota \left[0.7, 0.8\right], \left[-0.9, -0.8\right]+\iota \left[-0.7, -0.6\right]\right), \\ \left({\iota }_{4}, \left[0.1, 0.4\right]+\iota \left[0.2, 0.3\right], \left[-0.6, -0.5\right]+\iota \left[-0.9, -0.8\right]\right)\end{array}\right\}\\ \mathfrak{L}\left({j}_{4}\right)=\left\{\begin{array}{c}\left({\iota }_{1}, \left[0.3, 0.7\right]+\iota \left[0.4, 0.8\right], \left[-0.8,-0.7\right]+\iota \left[-0.9,-0.8\right]\right), \\ \left({\iota }_{2}, \left[0.8, 0.9\right]+\iota \left[0.6, 0.7\right], \left[-0.5, -0.3\right]+\iota \left[-0.4, -0.1\right]\right), \\ \left({\iota }_{3}, \left[0.5, 0.7\right]+\iota \left[0.8, 0.9\right], \left[-0.8, -0.7\right]+\iota \left[-0.6, -0.5\right]\right), \\ \left({\iota }_{4}, \left[0.3, 0.4\right]+\iota \left[0.5, 0.6\right], \left[-0.4, -0.3\right]+\iota \left[-0.3, -0.2\right]\right)\end{array}\right\}\end{array}\right\}$$

$$\left(\pounds , \text{\yen} \right)=\left\{\begin{array}{c}\pounds \left({j}_{1}\right)=\left\{\begin{array}{c}\left({\iota }_{1}, \left[0.4, 0.8\right]+\iota \left[0.6, 0.7\right], \left[-0.9,-0.7\right]+\iota \left[-0.8,-0.5\right]\right), \\ \left({\iota }_{2}, \left[0.5, 0.9\right]+\iota \left[0.7, 0.8\right], \left[-0.8, -0.4\right]+\iota \left[-0.7, -0.6\right]\right), \\ \left({\iota }_{3}, \left[0.6, 0.7\right]+\iota \left[0.7, 0.8\right], \left[-0.6, -0.5\right]+\iota \left[-0.4, -0.3\right]\right), \\ \left({\iota }_{4}, \left[0.5, 0.7\right]+\iota \left[0.8, 0.9\right], \left[-0.3, -0.2\right]+\iota \left[-0.8, -0.4\right]\right)\end{array}\right\}\\ \pounds \left({j}_{2}\right)=\left\{\begin{array}{c}\left({\iota }_{1}, \left[0.5, 0.9\right]+\iota \left[0.7, 0.8\right], \left[-0.8,-0.6\right]+\iota \left[-0.7,-0.4\right]\right), \\ \left({\iota }_{2}, \left[0.6, 0.8\right]+\iota \left[0.6, 0.7\right], \left[-0.9, -0.7\right]+\iota \left[-0.8, -0.5\right]\right), \\ \left({\iota }_{3}, \left[0.4, 0.6\right]+\iota \left[0.2, 0.3\right], \left[-0.2, -0.1\right]+\iota \left[-0.4, -0.3\right]\right), \\ \left({\iota }_{4}, \left[0.6, 0.9\right]+\iota \left[0.7, 0.8\right], \left[-0.5, -0.4\right]+\iota \left[-0.2, -0.1\right]\right)\end{array}\right\}\\ \pounds \left({j}_{4}\right)=\left\{\begin{array}{c}\left({\iota }_{1}, \left[0.3, 0.7\right]+\iota \left[0.2, 0.6\right], \left[-0.3,-0.2\right]+\iota \left[-0.2,-0.1\right]\right), \\ \left({\iota }_{2}, \left[0.1, 0.2\right]+\iota \left[0.3, 0.4\right], \left[-0.7, -0.5\right]+\iota \left[-0.9, -0.6\right]\right), \\ \left({\iota }_{3}, \left[0.3, 0.5\right]+\iota \left[0.1, 0.2\right], \left[-0.3, -0.2\right]+\iota \left[-0.5, -0.4\right]\right), \\ \left({\iota }_{4}, \left[0.6, 0.7\right]+\iota \left[0.4, 0.5\right], \left[-0.7, -0.6\right]+\iota \left[-0.8, -0.7\right]\right)\end{array}\right\}\end{array}\right\}$$

Then their restricted intersection is nominated as $\left( {{\text{\AA}}_{R} , {\text{T}}} \right) = \left( { \mathfrak{L} , \Gamma } \right) \cap_{R} \left( {\pounds , {\text{\yen}} } \right)$, where ${\text{T}} = \Gamma \cap {\text{\yen}} = \left\{ {j_{1} , j_{4} } \right\}$ and presented as

$$\left({\mathring{\text{A}}}_{R}, {\text{T}}\right)=\left\{\begin{array}{c}{\mathring{\text{A}}}_{R}\left({j}_{1}\right)=\left\{\begin{array}{c}\left({\iota }_{1}, \left[0.2, 0.6\right]+\iota \left[0.3, 0.4\right], \left[-0.9,-0.7\right]+\iota \left[-0.8,-0.5\right]\right), \\ \left({\iota }_{2}, \left[0.1, 0.5\right]+\iota \left[0.2, 0.3\right], \left[-0.8, -0.4\right]+\iota \left[-0.7, -0.6\right]\right), \\ \left({\iota }_{3}, \left[0.3, 0.4\right]+\iota \left[0.2, 0.3\right], \left[-0.6, -0.5\right]+\iota \left[-0.7, -0.6\right]\right), \\ \left({\iota }_{4}, \left[0.3, 0.5\right]+\iota \left[0.1, 0.2\right], \left[-0.8, -0.7\right]+\iota \left[-0.8, -0.4\right]\right)\end{array}\right\}\\ {\mathring{\text{A}}}_{R}\left({j}_{4}\right)=\left\{\begin{array}{c}\left({\iota }_{1}, \left[0.3, 0.7\right]+\iota \left[0.2, 0.6\right], \left[-0.8,-0.7\right]+\iota \left[-0.9,-0.8\right]\right), \\ \left({\iota }_{2}, \left[0.1, 0.2\right]+\iota \left[0.3, 0.4\right], \left[-0.7, -0.5\right]+\iota \left[-0.9, -0.6\right]\right), \\ \left({\iota }_{3}, \left[0.3, 0.5\right]+\iota \left[0.1, 0.2\right], \left[-0.8, -0.7\right]+\iota \left[-0.6, -0.5\right]\right), \\ \left({\iota }_{4}, \left[0.3, 0.4\right]+\iota \left[0.4, 0.5\right], \left[-0.7, -0.6\right]+\iota \left[-0.8, -0.7\right]\right)\end{array}\right\}\end{array}\right\}$$

#### Definition 19

The restricted union of two IVBCFSSs $\left(\mathfrak{L},\Gamma \right)$ and $\left( {\pounds ,{\text{\yen}} } \right)$ over $\k{\rm U}$ is an IVBCFSS $\left( {\overline{\text{I}}_{R} , {\text{T}}} \right)$, where ${\text{T}} = \Gamma \cap {\text{\yen}} \ne \emptyset$ and $\overline{\text{I}}_{R} :{\text{T}} \to IVBCFS\left( {\k{\rm U} } \right)$ considered as $\overline{\text{I}}_{R} \left( j \right) = \mathfrak{L} \left( j \right) \cup \pounds \left( j \right)$ and indicated by $\left( {\overline{\text{I}}_{R} , {\text{T}}} \right) = \left( { \mathfrak{L} ,{ }\Gamma } \right) \cup_{R} \left( {\pounds ,{\text{\yen}} } \right)$.

#### Example 6

Consider two IVBCFSSs $\left(\mathfrak{L},\Gamma \right)$ and $\left( {\pounds , {\text{\yen}} } \right)$ over $\k{\rm U}$ is obtainable in the Example 3. Their restricted union is nominated as $\left( {\overline{\text{I}}_{R} , {\text{T}}} \right) = \left( { \mathfrak{L} ,{ }\Gamma } \right) \cup_{R} \left( {\pounds , {\text{\yen}} } \right)$, where ${\text{T}}=\Gamma \cap \text{\yen} =\left\{{j}_{1}, {j}_{4}\right\}$ and presented as

$$\left({ {\bar{\text{I}}} }_{R}, {\text{T}}\right)=\left\{\begin{array}{c}{ {\bar{\text{I}}} }_{R}\left({j}_{1}\right)=\left\{\begin{array}{c}\left({\iota }_{1}, \left[0.4, 0.8\right]+\iota \left[0.6, 0.7\right], \left[-0.3,-0.1\right]+\iota \left[-0.5,-0.2\right]\right), \\ \left({\iota }_{2}, \left[0.5, 0.9\right]+\iota \left[0.7, 0.8\right], \left[-0.6, -0.2\right]+\iota \left[-0.4, -0.3\right]\right), \\ \left({\iota }_{3}, \left[0.6, 0.7\right]+\iota \left[0.7, 0.8\right], \left[-0.5, -0.4\right]+\iota \left[-0.4, -0.3\right]\right), \\ \left({\iota }_{4}, \left[0.5, 0.7\right]+\iota \left[0.8, 0.9\right], \left[-0.3, -0.2\right]+\iota \left[-0.6, -0.2\right]\right)\end{array}\right\}\\ { {\bar{\text{I}}} }_{R}\left({j}_{4}\right)=\left\{\begin{array}{c}\left({\iota }_{1}, \left[0.3, 0.7\right]+\iota \left[0.4, 0.8\right], \left[-0.3,-0.2\right]+\iota \left[-0.2,-0.1\right]\right), \\ \left({\iota }_{2}, \left[0.8, 0.9\right]+\iota \left[0.6, 0.7\right], \left[-0.5, -0.3\right]+\iota \left[-0.4, -0.1\right]\right), \\ \left({\iota }_{3}, \left[0.5, 0.7\right]+\iota \left[0.8, 0.9\right], \left[-0.3, -0.2\right]+\iota \left[-0.5, -0.4\right]\right), \\ \left({\iota }_{4}, \left[0.6, 0.7\right]+\iota \left[0.5, 0.6\right], \left[-0.4, -0.3\right]+\iota \left[-0.3, -0.2\right]\right)\end{array}\right\}\end{array}\right\}$$

#### Definition 20

The extended intersection of two IVBCFSSs $\left(\mathfrak{L},\Gamma \right)$ and $\left(\pounds , \text{\yen} \right)$ over $\k{\rm U}$ is an IVBCFSS $\left({{\text{\AA}}}_{E}, {\text{T}}\right)$, where ${\text{T}}=\Gamma \cup \text{\yen}$,

$$  {\text{{\AA}}}_{E} \left( j \right) = \left\{ {\begin{array}{*{20}l}    {{\mathfrak{L}}\left( j \right)} \hfill & {if\;j \in {\mathfrak{L}} - \pounds} \hfill  \\    {\pounds\left( j \right)} \hfill & {if\;j \in \pounds - {\mathfrak{L}}} \hfill  \\    {{\mathfrak{L}}\left( j \right) \cap \pounds\left( j \right)} \hfill & {if\;j \in {\mathfrak{L}} \cap \pounds} \hfill  \\   \end{array} } \right. $$

and indicated by $\left({{\text{\AA}}}_{E}, {\text{T}}\right)=\left(\mathfrak{L},\Gamma \right){\cap }_{E}\left(\pounds , \text{\yen} \right)$.

#### Example 7

Consider the two IVBCFSSs $\left( {{\mathfrak{L}}, \Gamma } \right)$ and $\left( {\pounds, {\text{\yen}} } \right)$ over $\k{\rm U}$ is obtainable in the example 3. Their extended intersection is nominated as $\left( {{\text{\AA}}_{E} , {\text{T}}} \right) = \left( {{\mathfrak{L}}, \Gamma } \right) \cap_{R} \left( {\pounds, {\text{\yen}} } \right)$, where ${\text{T}} = \Gamma \cup {\text{\yen}} = \left\{ {j_{1} , j_{2} , j_{3} , j_{4} } \right\}$ and is presented as

$$\left({\mathring{\text{A}}}_{E}, {\text{T}}\right)=\left\{\begin{array}{c}{\mathring{\text{A}}}_{E}\left({j}_{1}\right)=\left\{\begin{array}{c}\left({\iota }_{1}, \left[0.2, 0.6\right]+\iota \left[0.3, 0.4\right], \left[-0.9,-0.7\right]+\iota \left[-0.8,-0.5\right]\right), \\ \left({\iota }_{2}, \left[0.1, 0.5\right]+\iota \left[0.2, 0.3\right], \left[-0.8, -0.4\right]+\iota \left[-0.7, -0.6\right]\right), \\ \left({\iota }_{3}, \left[0.3, 0.4\right]+\iota \left[0.2, 0.3\right], \left[-0.6, -0.5\right]+\iota \left[-0.7, -0.6\right]\right), \\ \left({\iota }_{4}, \left[0.3, 0.5\right]+\iota \left[0.1, 0.2\right], \left[-0.8, -0.7\right]+\iota \left[-0.8, -0.4\right]\right)\end{array}\right\}\\ {\mathring{\text{A}}}_{E}\left({j}_{2}\right)=\left\{\begin{array}{c}\left({\iota }_{1}, \left[0.5, 0.9\right]+\iota \left[0.7, 0.8\right], \left[-0.8,-0.6\right]+\iota \left[-0.7,-0.4\right]\right), \\ \left({\iota }_{2}, \left[0.6, 0.8\right]+\iota \left[0.6, 0.7\right], \left[-0.9, -0.7\right]+\iota \left[-0.8, -0.5\right]\right), \\ \left({\iota }_{3}, \left[0.4, 0.6\right]+\iota \left[0.2, 0.3\right], \left[-0.2, -0.1\right]+\iota \left[-0.4, -0.3\right]\right), \\ \left({\iota }_{4}, \left[0.6, 0.9\right]+\iota \left[0.7, 0.8\right], \left[-0.5, -0.4\right]+\iota \left[-0.2, -0.1\right]\right)\end{array}\right\}\\ {\mathring{\text{A}}}_{E}\left({j}_{3}\right)=\left\{\begin{array}{c}\left({\iota }_{1}, \left[0.1, 0.5\right]+\iota \left[0.2, 0.3\right], \left[-0.4,-0.2\right]+\iota \left[-0.6,-0.3\right]\right), \\ \left({\iota }_{2}, \left[0.2, 0.4\right]+\iota \left[0.3, 0.4\right], \left[-0.3, -0.1\right]+\iota \left[-0.5, -0.2\right]\right), \\ \left({\iota }_{3}, \left[0.4, 0.6\right]+\iota \left[0.7, 0.8\right], \left[-0.9, -0.8\right]+\iota \left[-0.7, -0.6\right]\right), \\ \left({\iota }_{4}, \left[0.1, 0.4\right]+\iota \left[0.2, 0.3\right], \left[-0.6, -0.5\right]+\iota \left[-0.9, -0.8\right]\right)\end{array}\right\}\\ {\mathring{\text{A}}}_{E}\left({j}_{4}\right)=\left\{\begin{array}{c}\left({\iota }_{1}, \left[0.3, 0.7\right]+\iota \left[0.2, 0.6\right], \left[-0.8,-0.7\right]+\iota \left[-0.9,-0.8\right]\right), \\ \left({\iota }_{2}, \left[0.1, 0.2\right]+\iota \left[0.3, 0.4\right], \left[-0.7, -0.5\right]+\iota \left[-0.9, -0.6\right]\right), \\ \left({\iota }_{3}, \left[0.3, 0.5\right]+\iota \left[0.1, 0.2\right], \left[-0.8, -0.7\right]+\iota \left[-0.6, -0.5\right]\right), \\ \left({\iota }_{4}, \left[0.3, 0.4\right]+\iota \left[0.4, 0.5\right], \left[-0.7, -0.6\right]+\iota \left[-0.8, -0.7\right]\right)\end{array}\right\}\end{array}\right\}$$

#### Definition 21

The extended union of two IVBCFSSs $\left(\mathfrak{L},\Gamma \right)$ and $\left(\pounds , \text{\yen} \right)$ over $\k{\rm U}$ is an IVBCFSS $\left({ {\bar{\text{I}}} }_{E}, {\text{T}}\right)$, where ${\text{T}}=\Gamma \cup \text{\yen}$,

$$ {\text{{\AA}}}_{E} \left( j \right) = \left\{ {\begin{array}{*{20}l}    {{\mathfrak{L}}\left( j \right)} \hfill & {if\;j \in {\mathfrak{L}} - \pounds} \hfill  \\    {\pounds\left( j \right)} \hfill & {if\;j \in \pounds - {\mathfrak{L}}} \hfill  \\    {{\mathfrak{L}}\left( j \right) \cup \pounds\left( j \right)} \hfill & {if\;j \in {\mathfrak{L}} \cap \pounds} \hfill  \\   \end{array} } \right. $$

and indicated by $\left({ {\bar{\text{I}}} }_{E}, {\text{T}}\right)=\left(\mathfrak{L},\Gamma \right){\cup }_{E}\left(\pounds , \text{\yen} \right)$.

#### Example 8

Consider the two IVBCFSSs $\left(\mathfrak{L},\Gamma \right)$ and $\left(\pounds , \text{\yen} \right)$ over $\k{\rm U}$ is accessible in the Example 3. Their extended union is nominated as $\left({ {\bar{\text{I}}} }_{E}, {\text{T}}\right)=\left(\mathfrak{L},\Gamma \right){\cup }_{E}\left(\pounds , \text{\yen} \right)$, where ${\text{T}}=\Gamma \cup \text{\yen} =\left\{{j}_{1}, {j}_{2}, {j}_{3}, {j}_{5}\right\}$ and is presented as

$$\left({ {\bar{\text{I}}} }_{E}, {\text{T}}\right)=\left\{\begin{array}{c}{ {\bar{\text{I}}} }_{E}\left({j}_{1}\right)=\left\{\begin{array}{c}\left({\iota }_{1}, \left[0.4, 0.8\right]+\iota \left[0.6, 0.7\right], \left[-0.3,-0.1\right]+\iota \left[-0.5,-0.2\right]\right), \\ \left({\iota }_{2}, \left[0.5, 0.9\right]+\iota \left[0.7, 0.8\right], \left[-0.6, -0.2\right]+\iota \left[-0.4, -0.3\right]\right), \\ \left({\iota }_{3}, \left[0.6, 0.7\right]+\iota \left[0.7, 0.8\right], \left[-0.5, -0.4\right]+\iota \left[-0.4, -0.3\right]\right), \\ \left({\iota }_{4}, \left[0.7, 0.5\right]+\iota \left[0.9, 0.8\right], \left[-0.3, -0.2\right]+\iota \left[-0.6, -0.2\right]\right)\end{array}\right\}\\ { {\bar{\text{I}}} }_{E}\left({j}_{2}\right)=\left\{\begin{array}{c}\left({\iota }_{1}, \left[0.5, 0.9\right]+\iota \left[0.7, 0.8\right], \left[-0.8,-0.6\right]+\iota \left[-0.7,-0.4\right]\right), \\ \left({\iota }_{2}, \left[0.6, 0.8\right]+\iota \left[0.6, 0.7\right], \left[-0.9, -0.7\right]+\iota \left[-0.8, -0.5\right]\right), \\ \left({\iota }_{3}, \left[0.4, 0.6\right]+\iota \left[0.2, 0.3\right], \left[-0.2, -0.1\right]+\iota \left[-0.4, -0.3\right]\right), \\ \left({\iota }_{4}, \left[0.6, 0.9\right]+\iota \left[0.7, 0.8\right], \left[-0.5, -0.4\right]+\iota \left[-0.2, -0.1\right]\right)\end{array}\right\}\\ { {\bar{\text{I}}} }_{E}\left({j}_{3}\right)=\left\{\begin{array}{c}\left({\iota }_{1}, \left[0.1, 0.5\right]+\iota \left[0.2, 0.3\right], \left[-0.4,-0.2\right]+\iota \left[-0.6,-0.3\right]\right), \\ \left({\iota }_{2}, \left[0.2, 0.4\right]+\iota \left[0.3, 0.4\right], \left[-0.3, -0.1\right]+\iota \left[-0.5, -0.2\right]\right), \\ \left({\iota }_{3}, \left[0.4, 0.6\right]+\iota \left[0.7, 0.8\right], \left[-0.9, -0.8\right]+\iota \left[-0.7, -0.6\right]\right), \\ \left({\iota }_{4}, \left[0.1, 0.4\right]+\iota \left[0.2, 0.3\right], \left[-0.6, -0.5\right]+\iota \left[-0.9, -0.8\right]\right)\end{array}\right\}\\ { {\bar{\text{I}}} }_{E}\left({j}_{4}\right)=\left\{\begin{array}{c}\left({\iota }_{1}, \left[0.3, 0.7\right]+\iota \left[0.4, 0.8\right], \left[-0.3,-0.2\right]+\iota \left[-0.2,-0.1\right]\right), \\ \left({\iota }_{2}, \left[0.8, 0.9\right]+\iota \left[0.6, 0.7\right], \left[-0.5, -0.3\right]+\iota \left[-0.4, -0.1\right]\right), \\ \left({\iota }_{3}, \left[0.5, 0.7\right]+\iota \left[0.8, 0.9\right], \left[-0.3, -0.2\right]+\iota \left[-0.6, -0.5\right]\right), \\ \left({\iota }_{4}, \left[0.6, 0.7\right]+\iota \left[0.5, 0.6\right], \left[-0.4, -0.3\right]+\iota \left[-0.3, -0.2\right]\right)\end{array}\right\}\end{array}\right\}$$

#### Proposition 1

The IVBCFSSs $\left(\mathfrak{L},\Gamma \right)$, $\left(\pounds , \text{\yen} \right)$ and $\left(/\kern-9pt{\rm E}, {-}{\kern-9pt}{\rm D}\right)$ over $\k{\rm U}$ satisfies the following properties

1. $$\left(\mathfrak{L},\Gamma \right){\cup }_{E}\left(\mathfrak{L},\Gamma \right)=\left(\mathfrak{L},\Gamma \right)$$, $$\left(\mathfrak{L},\Gamma \right){\cap }_{E}\left(\mathfrak{L},\Gamma \right)=\left(\mathfrak{L},\Gamma \right)$$
2. $$\left(\mathfrak{L},\Gamma \right){\cup }_{E}\emptyset=\left(\mathfrak{L},\Gamma \right)$$, $$\left(\mathfrak{L},\Gamma \right){\cap }_{E}\emptyset=\emptyset$$
3. $$\left(\mathfrak{L},\Gamma \right){\cup }_{E}\left(\left(\mathfrak{L},\Gamma \right){\cap }_{E}\left(\pounds , \text{\yen} \right)\right)=\left(\mathfrak{L},\Gamma \right)$$, $$\left(\mathfrak{L},\Gamma \right){\cap }_{E}\left(\left(\mathfrak{L},\Gamma \right){\cup }_{E}\left(\pounds , \text{\yen} \right)\right)=\left(\mathfrak{L},\Gamma \right)$$
4. $$\left(\mathfrak{L},\Gamma \right){\cup }_{E}\left(\pounds , \text{\yen} \right)=\left(\pounds , \text{\yen} \right){\cup }_{E}\left(\mathfrak{L},\Gamma \right)$$, $$\left(\mathfrak{L},\Gamma \right){\cap }_{E}\left(\pounds , \text{\yen} \right)=\left(\pounds , \text{\yen} \right){\cap }_{E}\left(\mathfrak{L},\Gamma \right)$$
5. $$\left(\mathfrak{L},\Gamma \right){\cup }_{E}\left(\left(\pounds , \text{\yen} \right){\cup }_{E}\left(/\kern-9pt{\rm E}, {-}{\kern-9pt}{\rm D}\right)\right)=\left(\left(\mathfrak{L},\Gamma \right){\cup }_{E}\left(\pounds , \text{\yen} \right)\right){\cup }_{E}\left(/\kern-9pt{\rm E}, {-}{\kern-9pt}{\rm D}\right)$$ ,
$$\left(\mathfrak{L},\Gamma \right){\cap }_{E}\left(\left(\pounds , \text{\yen} \right){\cap }_{E}\left(/\kern-9pt{\rm E}, {-}{\kern-9pt}{\rm D}\right)\right)=\left(\left(\mathfrak{L},\Gamma \right){\cap }_{E}\left(\pounds , \text{\yen} \right)\right){\cap }_{E}\left(/\kern-9pt{\rm E}, {-}{\kern-9pt}{\rm D}\right)$$
6. $$\left(\mathfrak{L},\Gamma \right){\cap }_{E}\left(\left(\pounds , \text{\yen} \right){\cup }_{E}\left(/\kern-9pt{\rm E}, {-}{\kern-9pt}{\rm D}\right)\right)=\left(\left(\mathfrak{L},\Gamma \right){\cap }_{E}\left(\pounds , \text{\yen} \right)\right){\cup }_{E}\left(\left(\mathfrak{L},\Gamma \right){\cap }_{E}\left(/\kern-9pt{\rm E}, {-}{\kern-9pt}{\rm D}\right)\right)$$, $$\left(\mathfrak{L},\Gamma \right){\cup }_{E}\left(\left(\pounds , \text{\yen} \right){\cap }_{E}\left(/\kern-9pt{\rm E}, {-}{\kern-9pt}{\rm D}\right)\right)=\left(\left(\mathfrak{L},\Gamma \right){\cup }_{E}\left(\pounds , \text{\yen} \right)\right){\cap }_{E}\left(\left(\mathfrak{L},\Gamma \right){\cup }_{E}\left(/\kern-9pt{\rm E}, {-}{\kern-9pt}{\rm D}\right)\right)$$
7. $${\left(\left(\mathfrak{L},\Gamma \right){\cup }_{E}\left(\pounds , \text{\yen} \right)\right)}^{c}={\left(\mathfrak{L},\Gamma \right)}^{c}{{\cap }_{E}\left(\pounds , \text{\yen} \right)}^{c}$$, $${\left(\left(\mathfrak{L},\Gamma \right){\cap }_{E}\left(\pounds , \text{\yen} \right)\right)}^{c}={\left(\mathfrak{L},\Gamma \right)}^{c}{{\cup }_{E}\left(\pounds , \text{\yen} \right)}^{c}$$

#### Proposition 2

The IVBCFSSs $\left(\mathfrak{L},\Gamma \right)$, $\left(\pounds , \text{\yen} \right)$ and $\left(/\kern-9pt{\rm E}, {-}{\kern-9pt}{\rm D}\right)$ over $\k{\rm U}$ satisfies the following properties

1. $$\left( {{\mathfrak{L}}, \Gamma } \right) \cup_{R} \left( {{\mathfrak{L}}, \Gamma } \right) = \left( {{\mathfrak{L}}, \Gamma } \right)$$, $$\left( {{\mathfrak{L}}, \Gamma } \right) \cap_{R} \left( {{\mathfrak{L}}, \Gamma } \right) = \left( {{\mathfrak{L}}, \Gamma } \right)$$
2. $$\left( {{\mathfrak{L}}, \Gamma } \right) \cup_{R} \emptyset = \left( {{\mathfrak{L}}, \Gamma } \right)$$, $$\left( {{\mathfrak{L}}, \Gamma } \right) \cap_{R} \emptyset = \emptyset$$
3. $$\left( {{\mathfrak{L}}, \Gamma } \right) \cup_{R} \left( {\left( {{\mathfrak{L}}, \Gamma } \right) \cap_{R} \left( {\pounds, {\text{\yen}} } \right)} \right) = \left( {{\mathfrak{L}}, \Gamma } \right)$$, $$\left( {{\mathfrak{L}}, \Gamma } \right) \cap_{R} \left( {\left( {{\mathfrak{L}}, \Gamma } \right) \cup_{R} \left( {\pounds, {\text{\yen}} } \right)} \right) = \left( {{\mathfrak{L}}, \Gamma } \right)$$
4. $$\left( {{\mathfrak{L}}, \Gamma } \right) \cup_{R} \left( {\pounds, {\text{\yen}} } \right) = \left( {\pounds, {\text{\yen}} } \right) \cup_{R} \left( {{\mathfrak{L}}, \Gamma } \right)$$, $$\left( {{\mathfrak{L}}, \Gamma } \right) \cap_{R} \left( {\pounds, {\text{\yen}} } \right) = \left( {\pounds, {\text{\yen}} } \right) \cap_{R} \left( {{\mathfrak{L}}, \Gamma } \right)$$
5. $$\left( {{\mathfrak{L}}, \Gamma } \right) \cup_{R} \left( {\left( {\pounds, {\text{\yen}} } \right) \cup_{R} \left( {/\kern-9pt{\rm E} , {-}{\kern-9pt}{\rm D}} \right)} \right) = \left( {\left( {{\mathfrak{L}}, \Gamma } \right) \cup_{R} \left( {\pounds, {\text{\yen}} } \right)} \right) \cup_{R} \left( {/\kern-9pt{\rm E} , {-}{\kern-9pt}{\rm D}} \right)$$,
  $$\left( {{\mathfrak{L}}, \Gamma } \right) \cap_{R} \left( {\left( {\pounds, {\text{\yen}} } \right) \cap_{R} \left( {/\kern-9pt{\rm E} , {-}{\kern-9pt}{\rm D}} \right)} \right) = \left( {\left( {{\mathfrak{L}}, \Gamma } \right) \cap_{R} \left( {\pounds, {\text{\yen}} } \right)} \right) \cap_{R} \left( {/\kern-9pt{\rm E} , {-}{\kern-9pt}{\rm D}} \right)$$
6. $$ \left( {{\mathfrak{L}}, \Gamma } \right) \cap_{R} \left( {\left( {\pounds, {\text{\yen}} } \right) \cup_{R} \left( {/\kern-9pt{\rm E} , {-}{\kern-9pt}{\rm D}} \right)} \right) = \left( {\left( {{\mathfrak{L}}, \Gamma } \right) \cap_{R} \left( {\pounds, {\text{\yen}} } \right)} \right) \cup_{R} \left( {\left( {{\mathfrak{L}}, \Gamma } \right) \cap_{R} \left( {/\kern-9pt{\rm E} , {-}{\kern-9pt}{\rm D}} \right)} \right), $$
  $$\left( {{\mathfrak{L}}, \Gamma } \right) \cup_{R} \left( {\left( {\pounds, {\text{\yen}} } \right) \cap_{R} \left( {/\kern-9pt{\rm E} , {-}{\kern-9pt}{\rm D}} \right)} \right) = \left( {\left( {{\mathfrak{L}}, \Gamma } \right) \cup_{R} \left( {\pounds, {\text{\yen}} } \right)} \right) \cap_{R} \left( {\left( {{\mathfrak{L}}, \Gamma } \right) \cup_{R} \left( {/\kern-9pt{\rm E} , {-}{\kern-9pt}{\rm D}} \right)} \right)$$
7. $$\left( {\left( {{\mathfrak{L}}, \Gamma } \right) \cup_{R} \left( {\pounds, {\text{\yen}} } \right)} \right)^{c} = \left( {{\mathfrak{L}}, \Gamma } \right)^{c} \cap_{R} \left( {\pounds, {\text{\yen}} } \right)^{c}, \; \left( {\left( {{\mathfrak{L}}, \Gamma } \right) \cap_{R} \left( {\pounds, {\text{\yen}} } \right)} \right)^{c} = \left( {{\mathfrak{L}}, \Gamma } \right)^{c} \cup_{R} \left( {\pounds, {\text{\yen}} } \right)^{c}$$

### OR and AND operations on IVBCFSSs

Here, we will signify OR and AND operations for IVBCFSSs.

#### Definition 22

Let that two IVBCFSSs $\left(\mathfrak{L},\Gamma \right)$ and $\left(\pounds , \text{\yen} \right)$ over $\k{\rm U}$, then the OR operation is an IVBCFSS and demonstrated by $\left(\mathfrak{L},\Gamma \right)\vee \left(\pounds , \text{\yen} \right)=\left( {\bar{\text{I}}} , \Gamma \times \text{\yen} \right)$, where ${\bar{\text{I}}} \left(\mathfrak{a},\mathfrak{b}\right)=\mathfrak{L}\left(\mathfrak{a}\right)\cup \pounds \left(\mathfrak{b}\right) \forall \left(\mathfrak{a},\mathfrak{b}\right)\in \Gamma \times \text{\yen}$.

#### Example 9

Consider the two IVBCFSSs $\left(\mathfrak{L},\Gamma \right)$ and $\left(\pounds , \text{\yen} \right)$ over $\k{\rm U}$ interpreted in example 3. The OR operation is signified as $\left( {{\mathfrak{L}}, \Gamma } \right) \vee \left( {\pounds, {\text{\yen}} } \right) = \left( {\overline{\text{I}}, \Gamma \times {\text{\yen}} } \right)$ where $\Gamma \times {\text{\yen}} = \left\{ {j_{1} , j_{3} , j_{4} } \right\} \times \left\{ {j_{1} , j_{2} , j_{4} } \right\} = \left\{ {\left( {j_{1} , j_{1} } \right), \left( {j_{1} , j_{2} } \right), \left( {j_{1} , j_{4} } \right), \left( {j_{3} , j_{1} } \right), \left( {j_{3} , j_{2} } \right), \left( {j_{3} , j_{4} } \right), \left( {j_{4} , j_{1} } \right), \left( {j_{4} , j_{2} } \right), \left( {j_{4} , j_{4} } \right)} \right\}$ and is interpreted as

$$\left( {\bar{\text{I}}} , \Gamma \times \text{\yen} \right)=\left\{\begin{array}{c} {\bar{\text{I}}} \left({j}_{1}, {j}_{1}\right)=\left\{\begin{array}{c}\left({\iota }_{1}, \left[0.4, 0.8\right]+\iota \left[0.6, 0.7\right], \left[-0.3,-0.1\right]+\iota \left[-0.5,-0.2\right]\right), \\ \left({\iota }_{2}, \left[0.5, 0.9\right]+\iota \left[0.7, 0.8\right], \left[-0.6, -0.2\right]+\iota \left[-0.4, -0.3\right]\right), \\ \left({\iota }_{3}, \left[0.6, 0.7\right]+\iota \left[0.7, 0.8\right], \left[-0.5, -0.4\right]+\iota \left[-0.4, -0.3\right]\right), \\ \left({\iota }_{4}, \left[0.5, 0.7\right]+\iota \left[0.8, 0.9\right], \left[-0.3, -0.2\right]+\iota \left[-0.6, -0.2\right]\right)\end{array}\right\}\\  {\bar{\text{I}}} \left({j}_{1}, {j}_{2}\right)=\left\{\begin{array}{c}\left({\iota }_{1}, \left[0.5, 0.9\right]+\iota \left[0.7, 0.8\right], \left[-0.3,-0.1\right]+\iota \left[-0.5,-0.2\right]\right), \\ \left({\iota }_{2}, \left[0.6, 0.8\right]+\iota \left[0.6, 0.7\right], \left[-0.6, -0.2\right]+\iota \left[-0.4, -0.3\right]\right), \\ \left({\iota }_{3}, \left[0.4, 0.6\right]+\iota \left[0.2, 0.3\right], \left[-0.2, -0.1\right]+\iota \left[-0.4, -0.3\right]\right), \\ \left({\iota }_{4}, \left[0.6, 0.9\right]+\iota \left[0.7, 0.8\right], \left[-0.5, -0.4\right]+\iota \left[-0.2, -0.1\right]\right)\end{array}\right\}\\  {\bar{\text{I}}} \left({j}_{1}, {j}_{4}\right)=\left\{\begin{array}{c}\left({\iota }_{1}, \left[0.3, 0.7\right]+\iota \left[0.2, 0.6\right], \left[-0.3,-0.1\right]+\iota \left[-0.2,-0.1\right]\right), \\ \left({\iota }_{2}, \left[0.1, 0.5\right]+\iota \left[0.3, 0.4\right], \left[-0.6, -0.2\right]+\iota \left[-0.4, -0.3\right]\right), \\ \left({\iota }_{3}, \left[0.3, 0.5\right]+\iota \left[0.2, 0.3\right], \left[-0.3, -0.2\right]+\iota \left[-0.5, -0.4\right]\right), \\ \left({\iota }_{4}, \left[0.6, 0.7\right]+\iota \left[0.4, 0.5\right], \left[-0.7, -0.6\right]+\iota \left[-0.6, -0.2\right]\right)\end{array}\right\}\\  {\bar{\text{I}}} \left({j}_{3}, {j}_{1}\right)=\left\{\begin{array}{c}\left({\iota }_{1}, \left[0.4, 0.8\right]+\iota \left[0.6, 0.7\right], \left[-0.4,-0.2\right]+\iota \left[-0.6,-0.3\right]\right), \\ \left({\iota }_{2}, \left[0.5, 0.9\right]+\iota \left[0.7, 0.8\right], \left[-0.3, -0.1\right]+\iota \left[-0.5, -0.2\right]\right), \\ \left({\iota }_{3}, \left[0.6, 0.7\right]+\iota \left[0.7, 0.8\right], \left[-0.6, -0.5\right]+\iota \left[-0.4, -0.3\right]\right), \\ \left({\iota }_{4}, \left[0.5, 0.7\right]+\iota \left[0.8, 0.9\right], \left[-0.3, -0.2\right]+\iota \left[-0.8, -0.4\right]\right)\end{array}\right\}\\  {\bar{\text{I}}} \left({j}_{3}, {j}_{2}\right)=\left\{\begin{array}{c}\left({\iota }_{1}, \left[0.5, 0.9\right]+\iota \left[0.7, 0.8\right], \left[-0.4,-0.2\right]+\iota \left[-0.6,-0.3\right]\right), \\ \left({\iota }_{2}, \left[0.6, 0.8\right]+\iota \left[0.6, 0.7\right], \left[-0.3, -0.1\right]+\iota \left[-0.5, -0.2\right]\right), \\ \left({\iota }_{3}, \left[0.4, 0.6\right]+\iota \left[0.7, 0.8\right], \left[-0.2, -0.1\right]+\iota \left[-0.4, -0.3\right]\right), \\ \left({\iota }_{4}, \left[0.6, 0.9\right]+\iota \left[0.7, 0.8\right], \left[-0.5, -0.4\right]+\iota \left[-0.2, -0.1\right]\right)\end{array}\right\}\\  {\bar{\text{I}}} \left({j}_{3}, {j}_{4}\right)=\left\{\begin{array}{c}\left({\iota }_{1}, \left[0.3, 0.7\right]+\iota \left[0.2, 0.6\right], \left[-0.3,-0.2\right]+\iota \left[-0.2,-0.1\right]\right), \\ \left({\iota }_{2}, \left[0.2, 0.4\right]+\iota \left[0.3, 0.4\right], \left[-0.3, -0.1\right]+\iota \left[-0.5, -0.2\right]\right), \\ \left({\iota }_{3}, \left[0.3, 0.5\right]+\iota \left[0.7, 0.8\right], \left[-0.3, -0.2\right]+\iota \left[-0.5, -0.4\right]\right), \\ \left({\iota }_{4}, \left[0.6, 0.7\right]+\iota \left[0.4, 0.5\right], \left[-0.6, -0.5\right]+\iota \left[-0.8, -0.7\right]\right)\end{array}\right\}\\  {\bar{\text{I}}} \left({j}_{4}, {j}_{1}\right)=\left\{\begin{array}{c}\left({\iota }_{1}, \left[0.4, 0.8\right]+\iota \left[0.6, 0.8\right], \left[-0.8,-0.7\right]+\iota \left[-0.8,-0.5\right]\right), \\ \left({\iota }_{2}, \left[0.8, 0.9\right]+\iota \left[0.7, 0.8\right], \left[-0.5, -0.3\right]+\iota \left[-0.4, -0.1\right]\right), \\ \left({\iota }_{3}, \left[0.6, 0.7\right]+\iota \left[0.8, 0.9\right], \left[-0.6, -0.5\right]+\iota \left[-0.4, -0.3\right]\right), \\ \left({\iota }_{4}, \left[0.5, 0.7\right]+\iota \left[0.8, 0.9\right], \left[-0.3, -0.2\right]+\iota \left[-0.3, -0.2\right]\right)\end{array}\right\}\\  {\bar{\text{I}}} \left({j}_{4}, {j}_{2}\right)=\left\{\begin{array}{c}\left({\iota }_{1}, \left[0.5, 0.9\right]+\iota \left[0.7, 0.8\right], \left[-0.8,-0.6\right]+\iota \left[-0.7,-0.4\right]\right), \\ \left({\iota }_{2}, \left[0.8, 0.9\right]+\iota \left[0.6, 0.7\right], \left[-0.5, -0.3\right]+\iota \left[-0.4, -0.1\right]\right), \\ \left({\iota }_{3}, \left[0.5, 0.7\right]+\iota \left[0.8, 0.9\right], \left[-0.2, -0.1\right]+\iota \left[-0.4, -0.3\right]\right), \\ \left({\iota }_{4}, \left[0.6, 0.9\right]+\iota \left[0.7, 0.8\right], \left[-0.4, -0.3\right]+\iota \left[-0.2, -0.1\right]\right)\end{array}\right\}\\  {\bar{\text{I}}} \left({j}_{4}, {j}_{4}\right)=\left\{\begin{array}{c}\left({\iota }_{1}, \left[0.3, 0.7\right]+\iota \left[0.4, 0.8\right], \left[-0.3,-0.2\right]+\iota \left[-0.2,-0.1\right]\right), \\ \left({\iota }_{2}, \left[0.8, 0.9\right]+\iota \left[0.6, 0.7\right], \left[-0.5, -0.3\right]+\iota \left[-0.4, -0.1\right]\right), \\ \left({\iota }_{3}, \left[0.5, 0.7\right]+\iota \left[0.8, 0.9\right], \left[-0.3, -0.2\right]+\iota \left[-0.5, -0.4\right]\right), \\ \left({\iota }_{4}, \left[0.6, 0.7\right]+\iota \left[0.5, 0.6\right], \left[-0.4, -0.3\right]+\iota \left[-0.3, -0.2\right]\right)\end{array}\right\}\end{array}\right\}$$

#### Definition 23

Suppose that two IVBCFSSs $\left( {{\mathfrak{L}},{ }\Gamma } \right)$ and $\left( {\pounds, {\text{\yen}} } \right)$ over $\k{\rm U}$, then the AND operation is an IVBCFSS and demonstrated by $\left( {{\mathfrak{L}},{ }\Gamma } \right) \wedge \left( {\pounds,{\text{\yen}} } \right) = \left( {{\hat{\text{H}}}, \Gamma \times {\text{\yen}} } \right)$, where $\widehat{{\text{H}}}\left( {{\mathfrak{a}},{\mathfrak{b}}} \right) = {\mathfrak{L}}\left( {\mathfrak{a}} \right) \cap \pounds\left( {\mathfrak{b}} \right)\;\forall \;\left( {{\mathfrak{a}},{\mathfrak{b}}} \right) \in \Gamma \times {\text{\yen}}$.

#### Example 10

Consider the two IVBCFSSs $\left( {{\mathfrak{L}},{ }\Gamma } \right)$ and $\left( {\pounds, {\text{\yen}} } \right)$ over $\k{\rm U}$ interpreted in example 3. The AND operation is signified as $\left( {{\mathfrak{L}},\Gamma } \right) \wedge \left( {\pounds,{\text{\yen}} } \right) = \left( {{\hat{\text{H}}},\Gamma \times {\text{\yen}} } \right)$ where $\Gamma \times {\text{\yen}} = \left\{ {j_{1} , j_{3} , j_{4} } \right\} \times \left\{ {j_{1} , j_{2} , j_{4} } \right\} = \left\{ {\left( {j_{1} , j_{1} } \right), \left( {j_{1} , j_{2} } \right), \left( {j_{1} , j_{4} } \right), \left( {j_{3} , j_{1} } \right), \left( {j_{3} , j_{2} } \right), \left( {j_{3} , j_{4} } \right), \left( {j_{4} , j_{1} } \right), \left( {j_{4} , j_{2} } \right), \left( {j_{4} , j_{4} } \right)} \right\}$ and is interpreted as

$$\left(\hat{H} , \Gamma \times \text{\yen} \right)=\left\{\begin{array}{c}\hat{H} \left({j}_{1}, {j}_{1}\right)=\left\{\begin{array}{c}\left({\iota }_{1}, \left[0.2, 0.6\right]+\iota \left[0.3, 0.4\right], \left[-0.9, -0.7\right]+\iota \left[-0.8, -0.5\right]\right), \\ \left({\iota }_{2}, \left[0.1, 0.5\right]+\iota \left[0.2, 0.3\right], \left[-0.8, -0.4\right]+\iota \left[-0.7, -0.6\right]\right), \\ \left({\iota }_{3}, \left[0.3, 0.4\right]+\iota \left[0.2, 0.3\right], \left[-0.6, -0.5\right]+\iota \left[-0.7, -0.6\right]\right), \\ \left({\iota }_{4}, \left[0.3, 0.5\right]+\iota \left[0.1, 0.2\right], \left[-0.8, -0.7\right]+\iota \left[-0.8, -0.4\right]\right)\end{array}\right\}\\ \hat{H} \left({j}_{1}, {j}_{2}\right)=\left\{\begin{array}{c}\left({\iota }_{1}, \left[0.2, 0.6\right]+\iota \left[0.3, 0.4\right], \left[-0.8, -0.6\right]+\iota \left[-0.7, -0.4\right]\right), \\ \left({\iota }_{2}, \left[0.1, 0.5\right]+\iota \left[0.2, 0.3\right], \left[-0.9, -0.7\right]+\iota \left[-0.8, -0.5\right]\right), \\ \left({\iota }_{3}, \left[0.3, 0.4\right]+\iota \left[0.2, 0.3\right], \left[-0.5, -0.4\right]+\iota \left[-0.7, -0.3\right]\right), \\ \left({\iota }_{4}, \left[0.3, 0.4\right]+\iota \left[0.1, 0.2\right], \left[-0.8, -0.7\right]+\iota \left[-0.6, -0.2\right]\right)\end{array}\right\}\\ \hat{H} \left({j}_{1}, {j}_{4}\right)=\left\{\begin{array}{c}\left({\iota }_{1}, \left[0.2, 0.6\right]+\iota \left[0.2, 0.4\right], \left[-0.3, -0.2\right]+\iota \left[-0.5, -0.2\right]\right), \\ \left({\iota }_{2}, \left[0.1, 0.2\right]+\iota \left[0.2, 0.3\right], \left[-0.7, -0.5\right]+\iota \left[-0.9, -0.6\right]\right), \\ \left({\iota }_{3}, \left[0.3, 0.4\right]+\iota \left[0.1, 0.2\right], \left[-0.5, -0.4\right]+\iota \left[-0.7, -0.6\right]\right), \\ \left({\iota }_{4}, \left[0.3, 0.5\right]+\iota \left[0.1, 0.2\right], \left[-0.8, -0.7\right]+\iota \left[-0.8, -0.7\right]\right)\end{array}\right\}\\ \hat{H} \left({j}_{3}, {j}_{1}\right)=\left\{\begin{array}{c}\left({\iota }_{1}, \left[0.1, 0.5\right]+\iota \left[0.2, 0.3\right], \left[-0.9, -0.7\right]+\iota \left[-0.8, -0.5\right]\right), \\ \left({\iota }_{2}, \left[0.2, 0.4\right]+\iota \left[0.3, 0.4\right], \left[-0.8, -0.4\right]+\iota \left[-0.7, -0.6\right]\right), \\ \left({\iota }_{3}, \left[0.3, 0.4\right]+\iota \left[0.7, 0.8\right], \left[-0.9, -0.8\right]+\iota \left[-0.7, -0.6\right]\right), \\ \left({\iota }_{4}, \left[0.1, 0.4\right]+\iota \left[0.2, 0.3\right], \left[-0.6, -0.5\right]+\iota \left[-0.9, -0.8\right]\right)\end{array}\right\}\\ \hat{H} \left({j}_{3}, {j}_{2}\right)=\left\{\begin{array}{c}\left({\iota }_{1}, \left[0.1, 0.5\right]+\iota \left[0.2, 0.3\right], \left[-0.8, -0.6\right]+\iota \left[-0.7, -0.4\right]\right), \\ \left({\iota }_{2}, \left[0.2, 0.4\right]+\iota \left[0.3, 0.4\right], \left[-0.9, -0.8\right]+\iota \left[-0.8, -0.6\right]\right), \\ \left({\iota }_{3}, \left[0.4, 0.6\right]+\iota \left[0.2, 0.3\right], \left[-0.9, -0.8\right]+\iota \left[-0.7, -0.6\right]\right), \\ \left({\iota }_{4}, \left[0.1, 0.4\right]+\iota \left[0.2, 0.3\right], \left[-0.6, -0.5\right]+\iota \left[-0.9, -0.8\right]\right)\end{array}\right\}\\ \hat{H} \left({j}_{3}, {j}_{4}\right)=\left\{\begin{array}{c}\left({\iota }_{1}, \left[0.1, 0.5\right]+\iota \left[0.2, 0.3\right], \left[-0.4, -0.2\right]+\iota \left[-0.6, -0.3\right]\right), \\ \left({\iota }_{2}, \left[0.1, 0.2\right]+\iota \left[0.3, 0.4\right], \left[-0.7, -0.5\right]+\iota \left[-0.9, -0.6\right]\right), \\ \left({\iota }_{3}, \left[0.3, 0.5\right]+\iota \left[0.1, 0.2\right], \left[-0.9, -0.8\right]+\iota \left[-0.7, -0.6\right]\right), \\ \left({\iota }_{4}, \left[0.1, 0.4\right]+\iota \left[0.2, 0.3\right], \left[-0.7, -0.6\right]+\iota \left[-0.9, -0.8\right]\right)\end{array}\right\}\\ \hat{H} \left({j}_{4}, {j}_{1}\right)=\left\{\begin{array}{c}\left({\iota }_{1}, \left[0.3, 0.7\right]+\iota \left[0.4, 0.7\right], \left[-0.9, -0.7\right]+\iota \left[-0.9, -0.8\right]\right), \\ \left({\iota }_{2}, \left[0.5, 0.9\right]+\iota \left[0.6, 0.7\right], \left[-0.8, -0.4\right]+\iota \left[-0.7, -0.6\right]\right), \\ \left({\iota }_{3}, \left[0.5, 0.7\right]+\iota \left[0.7, 0.8\right], \left[-0.8, -0.7\right]+\iota \left[-0.6, -0.5\right]\right), \\ \left({\iota }_{4}, \left[0.3, 0.4\right]+\iota \left[0.5, 0.6\right], \left[-0.4, -0.3\right]+\iota \left[-0.8, -0.4\right]\right)\end{array}\right\}\\ \hat{H} \left({j}_{4}, {j}_{2}\right)=\left\{\begin{array}{c}\left({\iota }_{1}, \left[0.3, 0.7\right]+\iota \left[0.4, 0.8\right], \left[-0.8, -0.7\right]+\iota \left[-0.9, -0.8\right]\right), \\ \left({\iota }_{2}, \left[0.6, 0.8\right]+\iota \left[0.6, 0.7\right], \left[-0.9, -0.7\right]+\iota \left[-0.8, -0.5\right]\right), \\ \left({\iota }_{3}, \left[0.4, 0.6\right]+\iota \left[0.2, 0.3\right], \left[-0.8, -0.7\right]+\iota \left[-0.6, -0.5\right]\right), \\ \left({\iota }_{4}, \left[0.3, 0.4\right]+\iota \left[0.5, 0.6\right], \left[-0.5, -0.4\right]+\iota \left[-0.3, -0.2\right]\right)\end{array}\right\}\\ \hat{H} \left({j}_{4}, {j}_{4}\right)=\left\{\begin{array}{c}\left({\iota }_{1}, \left[0.3, 0.7\right]+\iota \left[0.2, 0.6\right], \left[-0.8, -0.7\right]+\iota \left[-0.9, -0.8\right]\right), \\ \left({\iota }_{2}, \left[0.1, 0.2\right]+\iota \left[0.3, 0.4\right], \left[-0.7, -0.5\right]+\iota \left[-0.9, -0.6\right]\right), \\ \left({\iota }_{3}, \left[0.3, 0.5\right]+\iota \left[0.1, 0.2\right], \left[-0.8, -0.7\right]+\iota \left[-0.6, -0.5\right]\right), \\ \left({\iota }_{4}, \left[0.3, 0.4\right]+\iota \left[0.4, 0.5\right], \left[-0.7, -0.6\right]+\iota \left[-0.8, -0.7\right]\right)\end{array}\right\}\end{array}\right\}$$

#### Proposition 3

The IVBCFSSs $\left(\mathfrak{L},\Gamma \right)$, and $\left( {\pounds,{\text{\yen}} } \right)$ over $\k{\rm U}$ satisfies the idempotent property i.e.

1. $$\left(\mathfrak{L},\Gamma \right)\vee \left(\mathfrak{L},\Gamma \right)=\left(\mathfrak{L},\Gamma \right)$$
2. $$\left(\mathfrak{L},\Gamma \right)\wedge \left(\mathfrak{L},\Gamma \right)=\left(\mathfrak{L},\Gamma \right)$$

#### Proof

1. Let $\left( {{\mathfrak{L}}, \Gamma } \right) \vee \left( {{\mathfrak{L}}, \Gamma } \right) = \left( {\overline{\text{I}}, \Gamma \times \Gamma } \right)$ and ${\mathfrak{a}} \in \Gamma$. Then $\overline{\text{I}}\left( {{\mathfrak{a}}, {\mathfrak{a}}} \right) = {\mathfrak{L}}\left( {\mathfrak{a}} \right) \cup {\mathfrak{L}}\left( {\mathfrak{a}} \right)$, since ${\mathfrak{L}}\left( {\mathfrak{a}} \right) \cup {\mathfrak{L}}\left( {\mathfrak{a}} \right) = {\mathfrak{L}}\left( {\mathfrak{a}} \right) \Rightarrow \overline{\text{I}}\left( {{\mathfrak{a}}, {\mathfrak{a}}} \right) = {\mathfrak{L}}\left( {\mathfrak{a}} \right) \Rightarrow \left( {\overline{\text{I}}, \Gamma \times \Gamma } \right) = \left( {{\mathfrak{L}}, \Gamma } \right)$. Hence $\left( {{\mathfrak{L}}, \Gamma } \right) \vee \left( {{\mathfrak{L}}, \Gamma } \right) = \left( {{\mathfrak{L}}, \Gamma } \right)$.
2. Let $\left( {{\mathfrak{L}},{ }\Gamma } \right) \wedge \left( {{\mathfrak{L}},{ }\Gamma } \right) = \left( {{\hat{\text{H}}},{ }\Gamma \times \Gamma } \right)$ and ${\mathfrak{a}} \in \Gamma$. Then ${\hat{\text{H}}}\left( {{\mathfrak{a}},{ }{\mathfrak{a}}} \right) = {\mathfrak{L}}\left( {\mathfrak{a}} \right) \cap {\mathfrak{L}}\left( {\mathfrak{a}} \right)$, since ${\mathfrak{L}}\left( {\mathfrak{a}} \right) \cap {\mathfrak{L}}\left( {\mathfrak{a}} \right) = {\mathfrak{L}}\left( {\mathfrak{a}} \right) \Rightarrow {\hat{\text{H}}}\left( {{\mathfrak{a}},{ }{\mathfrak{a}}} \right) = {\mathfrak{L}}\left( {\mathfrak{a}} \right) \Rightarrow \left( {{\hat{\text{H}}},{ }\Gamma \times \Gamma } \right) = \left( {{\mathfrak{L}},{ }\Gamma } \right)$. Hence $\left( {{\mathfrak{L}},{ }\Gamma } \right) \wedge \left( {{\mathfrak{L}},{ }\Gamma } \right) = \left( {{\mathfrak{L}},{ }\Gamma } \right)$.

## Interval-valued bipolar complex fuzzy soft AOs

In this segment, we demonstrate IVBCFSAAOs and IVBCFSGAOs with properties.

### Interval-valued bipolar complex fuzzy soft average aggregation operator

#### Definition 24

Suppose

$${\mathfrak{L}}_{\upsilon \nu } = \left( {{\rm E}_{{{\mathfrak{L}}_{\upsilon \nu } }}^{ + } , {\rm E}_{{{\mathfrak{L}}_{\upsilon \nu } }}^{ - } } \right) = \left( {\imath_{\upsilon } , \begin{array}{*{20}c} {\left[ {{\rm N}_{{{\mathfrak{L}}_{\upsilon \nu } }}^{L + } , {\rm O}_{{{\mathfrak{L}}_{\upsilon \nu } }}^{U + } } \right] + \iota \left[ {{\rm N}_{{{\mathfrak{L}}_{\upsilon \nu } }}^{L + } , {\rm O}_{{{\mathfrak{L}}_{\upsilon \nu } }}^{U + } } \right], } \\ {\left[ {{\rm N}_{{{\mathfrak{L}}_{\upsilon \nu } }}^{L - } , {\rm O}_{{{\mathfrak{L}}_{\upsilon \nu } }}^{U - } } \right] + \iota \left[ {{\rm N}_{{{\mathfrak{L}}_{\upsilon \nu } }}^{L - } , {\rm O}_{{{\mathfrak{L}}_{\upsilon \nu } }}^{U - } } \right]} \\ \end{array} } \right)\left( {\upsilon = 1, 2, \ldots , {\tilde{r}} ; \nu = 1, 2, \ldots , {\tilde{s}} } \right)$$

be the assortment of IVBCFSNs and let $n_{\upsilon , } t_{\nu }$ are the weight vectors (WVs) for experts $\imath_{\upsilon } ^{\prime}s$ and $j_{\nu }^{\prime}s$ respectively, holding $n_{\upsilon } \ge 0, t_{\nu } \ge 0$ such that $\mathop \sum \nolimits_{\upsilon = 1}^{{{\tilde{r}}}} n_{\upsilon } = 1$ and $\mathop \sum \nolimits_{\nu = 1}^{{{\tilde{s}} }} t_{\nu } = 1$. The IVBCFS weighted averaging aggregation (IVBCFSWAA) operator is the function $IVBCFSWAA:{\mathfrak{L}}^{n} \to {\mathfrak{L}}$ such that

7 $$ IVBCFSWAA\left( {{\mathfrak{L}}_{11} , {\mathfrak{L}}_{12} , \ldots , {\mathfrak{L}}_{{{\tilde{r}} {\tilde{s}} }} } \right) = \oplus_{\nu = 1}^{{{\tilde{s}} }} t_{\nu } \left( { \oplus_{\upsilon = 1}^{{{\tilde{r}} }} n_{\upsilon } {\mathfrak{L}}_{\upsilon \nu } } \right) $$

#### Theorem 1

Suppose

$${\mathfrak{L}}_{\upsilon \nu }=\left({\rm E}_{{\mathfrak{L}}_{\upsilon \nu }}^{+}, {\rm E}_{{\mathfrak{L}}_{\upsilon \nu }}^{-}\right)=\left({\iota }_{\upsilon }, \begin{array}{c}\left[{\rm N}_{{\mathfrak{L}}_{\upsilon \nu }}^{L+}, {\rm O}_{{\mathfrak{L}}_{\upsilon \nu }}^{U+}\right]+\iota \left[{\rm N}_{{\mathfrak{L}}_{\upsilon \nu }}^{L+}, {\rm O}_{{\mathfrak{L}}_{\upsilon \nu }}^{U+}\right], \\ \left[{\rm N}_{{\mathfrak{L}}_{\upsilon \nu }}^{L-}, {\rm O}_{{\mathfrak{L}}_{\upsilon \nu }}^{U-}\right]+\iota \left[{\rm N}_{{\mathfrak{L}}_{\upsilon \nu }}^{L-}, {\rm O}_{{\mathfrak{L}}_{\upsilon \nu }}^{U-}\right]\end{array}\right)\left(\upsilon =1, 2, \dots , {\tilde{r}}; \nu =1, 2, \dots , {\tilde{s}}\right)$$

be the collection of IVBCFSNs, then aggregated value of them usage the IVBCFSWAA operator is also a IVBCFSN, and

8 $$ IVBCFSWAA\left( {{\mathfrak{L}}_{11} , {\mathfrak{L}}_{12} , \ldots , {\mathfrak{L}}_{{{\tilde{r}} {\tilde{s}} }} } \right) = \left( \begin{gathered} \left[ {1 - \mathop \prod \limits_{\nu = 1}^{{{\tilde{s}} }} \left( {\mathop \prod \limits_{\upsilon = 1}^{{{\tilde{r}} }} \left( {1 - {\rm N}_{{{\mathfrak{L}}_{\upsilon \nu } }}^{L + } } \right)^{{n_{\upsilon } }} } \right)^{{t_{\nu } }} , 1 - \mathop \prod \limits_{\nu = 1}^{{{\tilde{s}} }} \left( {\mathop \prod \limits_{\upsilon = 1}^{{{\tilde{r}} }} \left( {1 - {\rm O}_{{{\mathfrak{L}}_{\upsilon \nu } }}^{U + } } \right)^{{n_{\upsilon } }} } \right)^{{t_{\nu } }} } \right] \hfill \\ + \iota \left[ {1 - \mathop \prod \limits_{\nu = 1}^{{{\tilde{s}} }} \left( {\mathop \prod \limits_{\upsilon = 1}^{{{\tilde{r}} }} \left( {1 - {\rm N}_{{{\mathfrak{L}}_{\upsilon \nu } }}^{L + } } \right)^{{n_{\upsilon } }} } \right)^{{t_{\nu } }} , 1 - \mathop \prod \limits_{\nu = 1}^{{{\tilde{s}} }} \left( {\mathop \prod \limits_{\upsilon = 1}^{{{\tilde{r}} }} \left( {1 - {\rm O}_{{{\mathfrak{L}}_{\upsilon \nu } }}^{U + } } \right)^{{n_{\upsilon } }} } \right)^{{t_{\nu } }} } \right], \hfill \\ \begin{array}{*{20}c} {\left[ { - \mathop \prod \limits_{\nu = 1}^{{{\tilde{s}} }} \left( {\mathop \prod \limits_{\upsilon = 1}^{{{\tilde{r}} }} \left| {{\rm N}_{{{\mathfrak{L}}_{\upsilon \nu } }}^{L - } } \right|^{{n_{\upsilon } }} } \right)^{{t_{\nu } }} , - \mathop \prod \limits_{\nu = 1}^{{{\tilde{s}} }} \left( {\mathop \prod \limits_{\upsilon = 1}^{{{\tilde{r}} }} \left| {{\rm O}_{{{\mathfrak{L}}_{\upsilon \nu } }}^{U - } } \right|^{{n_{\upsilon } }} } \right)^{{t_{\nu } }} } \right]} \\ { + \iota \left[ { - \mathop \prod \limits_{\nu = 1}^{{{\tilde{s}} }} \left( {\mathop \prod \limits_{\upsilon = 1}^{{{\tilde{r}} }} \left| {{\rm N}_{{{\mathfrak{L}}_{\upsilon \nu } }}^{L - } } \right|^{{n_{\upsilon } }} } \right)^{{t_{\nu } }} , - \mathop \prod \limits_{\nu = 1}^{{{\tilde{s}} }} \left( {\mathop \prod \limits_{\upsilon = 1}^{{{\tilde{r}} }} \left| {{\rm O}_{{{\mathfrak{L}}_{\upsilon \nu } }}^{U - } } \right|^{{n_{\upsilon } }} } \right)^{{t_{\nu } }} } \right] } \\ \end{array} \hfill \\ \end{gathered} \right) $$

#### Proof

For ${\tilde{r}} = 1$, we get $n_{1} = 1$,

$$ IVBCFSWAA\left( {{\mathfrak{L}}_{11} , {\mathfrak{L}}_{12} , \ldots , {\mathfrak{L}}_{{{\tilde{r}} {\tilde{s}} }} } \right) = \oplus_{\nu = 1}^{{{\tilde{s}} }} t_{\nu } \left( {{\mathfrak{L}}_{1\nu } } \right) $$

$$=\left(\begin{array}{c}\left[1-\prod_{\nu =1}^{{\tilde{s}}}{\left(1-{\rm N}_{{\mathfrak{L}}_{\upsilon \nu }}^{L+}\right)}^{{t}_{\nu }}, 1-\prod_{\nu =1}^{{\tilde{s}}}{\left(1-{\rm O}_{{\mathfrak{L}}_{\upsilon \nu }}^{U+}\right)}^{{t}_{\nu }}\right]\\ +\iota \left[1-\prod_{\nu =1}^{{\tilde{s}}}{\left(1-{\rm N}_{{\mathfrak{L}}_{\upsilon \nu }}^{L+}\right)}^{{t}_{\nu }}, 1-\prod_{\nu =1}^{{\tilde{s}}}{\left(1-{\rm O}_{{\mathfrak{L}}_{\upsilon \nu }}^{U+}\right)}^{{t}_{\nu }}\right],\\ \begin{array}{c}\left[-\prod_{\nu =1}^{{\tilde{s}}}{\left|{\rm N}_{\mathfrak{L}}^{L-}\right|}^{{t}_{\nu }},-\prod_{\nu =1}^{{\tilde{s}}}{\left|{\rm O}_{\mathfrak{L}}^{U-}\right|}^{{t}_{\nu }}\right]\\ +\iota \left[-\prod_{\nu =1}^{{\tilde{s}}}{\left|{\rm N}_{\mathfrak{L}}^{L-}\right|}^{{t}_{\nu }},-\prod_{\nu =1}^{{\tilde{s}}}{\left|{\rm O}_{\mathfrak{L}}^{U-}\right|}^{{t}_{\nu }}\right] \end{array}\end{array}\right)$$

$$=\left(\begin{array}{c}\left[1-\prod_{\nu =1}^{{\tilde{s}}}{\left(\prod_{\upsilon =1}^{1}{\left(1-{\rm N}_{{\mathfrak{L}}_{\upsilon \nu }}^{L+}\right)}^{{n}_{\upsilon }}\right)}^{{t}_{\nu }}, 1-\prod_{\nu =1}^{{\tilde{s}}}{\left(\prod_{\upsilon =1}^{1}{\left(1-{\rm O}_{{\mathfrak{L}}_{\upsilon \nu }}^{U+}\right)}^{{n}_{\upsilon }}\right)}^{{t}_{\nu }}\right]\\ +\iota \left[1-\prod_{\nu =1}^{{\tilde{s}}}{\left(\prod_{\upsilon =1}^{1}{\left(1-{\rm N}_{{\mathfrak{L}}_{\upsilon \nu }}^{L+}\right)}^{{n}_{\upsilon }}\right)}^{{t}_{\nu }}, 1-\prod_{\nu =1}^{{\tilde{s}}}{\left(\prod_{\upsilon =1}^{1}{\left(1-{\rm O}_{{\mathfrak{L}}_{\upsilon \nu }}^{U+}\right)}^{{n}_{\upsilon }}\right)}^{{t}_{\nu }}\right],\\ \begin{array}{c}\left[-\prod_{\nu =1}^{{\tilde{s}}}{\left(\prod_{\upsilon =1}^{1}{\left|{\rm N}_{{\mathfrak{L}}_{\upsilon \nu }}^{L-}\right|}^{{n}_{\upsilon }}\right)}^{{t}_{\nu }},-\prod_{\nu =1}^{{\tilde{s}}}{\left(\prod_{\upsilon =1}^{1}{\left|{\rm O}_{{\mathfrak{L}}_{\upsilon \nu }}^{U-}\right|}^{{n}_{\upsilon }}\right)}^{{t}_{\nu }}\right]\\ +\iota \left[-\prod_{\nu =1}^{{\tilde{s}}}{\left(\prod_{\upsilon =1}^{1}{\left|{\rm N}_{{\mathfrak{L}}_{\upsilon \nu }}^{L-}\right|}^{{n}_{\upsilon }}\right)}^{{t}_{\nu }},-\prod_{\nu =1}^{{\tilde{s}}}{\left(\prod_{\upsilon =1}^{1}{\left|{\rm O}_{{\mathfrak{L}}_{\upsilon \nu }}^{U-}\right|}^{{n}_{\upsilon }}\right)}^{{t}_{\nu }}\right] \end{array}\end{array}\right)$$

Again, for ${\tilde{s}} = 1$, we get $t_{1} = 1$ and hence,

$$IVBCFSWAA\left({\mathfrak{L}}_{11}, {\mathfrak{L}}_{12}, \dots , {\mathfrak{L}}_{{\tilde{r}}{\tilde{s}}}\right)={  \oplus  }_{\nu =1}^{{\tilde{s}}}{n}_{\upsilon }\left({\mathfrak{L}}_{\upsilon 1}\right)=\left(\begin{array}{c}\left[1-\prod_{\upsilon =1}^{{\tilde{r}}}{\left(1-{\rm N}_{{\mathfrak{L}}_{\upsilon \nu }}^{L+}\right)}^{{n}_{\upsilon }}, 1-\prod_{\upsilon =1}^{{\tilde{r}}}{\left(1-{\rm O}_{{\mathfrak{L}}_{\upsilon \nu }}^{U+}\right)}^{{n}_{\upsilon }}\right]\\ +\iota \left[1-\prod_{\upsilon =1}^{{\tilde{r}}}{\left(1-{\rm N}_{{\mathfrak{L}}_{\upsilon \nu }}^{L+}\right)}^{{n}_{\upsilon }}, 1-\prod_{\upsilon =1}^{{\tilde{r}}}{\left(1-{\rm O}_{{\mathfrak{L}}_{\upsilon \nu }}^{U+}\right)}^{{n}_{\upsilon }}\right],\\ \begin{array}{c}\left[-\prod_{\upsilon =1}^{{\tilde{r}}}{\left|{\rm N}_{{\mathfrak{L}}_{\upsilon \nu }}^{L-}\right|}^{{n}_{\upsilon }}, -\prod_{\upsilon =1}^{{\tilde{r}}}{\left|{\rm O}_{{\mathfrak{L}}_{\upsilon \nu }}^{U-}\right|}^{{n}_{\upsilon }}\right]\\ +\iota \left[-\prod_{\upsilon =1}^{{\tilde{r}}}{\left|{\rm N}_{{\mathfrak{L}}_{\upsilon \nu }}^{L-}\right|}^{{n}_{\upsilon }}, -\prod_{\upsilon =1}^{{\tilde{r}}}{\left|{\rm O}_{{\mathfrak{L}}_{\upsilon \nu }}^{U-}\right|}^{{n}_{\upsilon }}\right] \end{array}\end{array}\right)$$

$$=\left(\begin{array}{c}\left[1-\prod_{\nu =1}^{1}{\left(\prod_{\upsilon =1}^{{\tilde{r}}}{\left(1-{\rm N}_{{\mathfrak{L}}_{\upsilon \nu }}^{L+}\right)}^{{n}_{\upsilon }}\right)}^{{t}_{\nu }}, 1-\prod_{\nu =1}^{1}{\left(\prod_{\upsilon =1}^{{\tilde{r}}}{\left(1-{\rm O}_{{\mathfrak{L}}_{\upsilon \nu }}^{U+}\right)}^{{n}_{\upsilon }}\right)}^{{t}_{\nu }}\right]\\ +\iota \left[1-\prod_{\nu =1}^{1}{\left(\prod_{\upsilon =1}^{{\tilde{r}}}{\left(1-{\rm N}_{{\mathfrak{L}}_{\upsilon \nu }}^{L+}\right)}^{{n}_{\upsilon }}\right)}^{{t}_{\nu }}, 1-\prod_{\nu =1}^{1}{\left(\prod_{\upsilon =1}^{{\tilde{r}}}{\left(1-{\rm O}_{{\mathfrak{L}}_{\upsilon \nu }}^{U+}\right)}^{{n}_{\upsilon }}\right)}^{{t}_{\nu }}\right],\\ \begin{array}{c}\left[-\prod_{\nu =1}^{1}{\left(\prod_{\upsilon =1}^{{\tilde{r}}}{\left|{\rm N}_{{\mathfrak{L}}_{\upsilon \nu }}^{L-}\right|}^{{n}_{\upsilon }}\right)}^{{t}_{\nu }},-\prod_{\nu =1}^{1}{\left(\prod_{\upsilon =1}^{{\tilde{r}}}{\left|{\rm O}_{{\mathfrak{L}}_{\upsilon \nu }}^{U-}\right|}^{{n}_{\upsilon }}\right)}^{{t}_{\nu }}\right]\\ +\iota \left[-\prod_{\nu =1}^{1}{\left(\prod_{\upsilon =1}^{{\tilde{r}}}{\left|{\rm N}_{{\mathfrak{L}}_{\upsilon \nu }}^{L-}\right|}^{{n}_{\upsilon }}\right)}^{{t}_{\nu }},-\prod_{\nu =1}^{1}{\left(\prod_{\upsilon =1}^{{\tilde{r}}}{\left|{\rm O}_{{\mathfrak{L}}_{\upsilon \nu }}^{U-}\right|}^{{n}_{\upsilon }}\right)}^{{t}_{\nu }}\right] \end{array}\end{array}\right)$$

Thus, (8) is true for ${\tilde{r}} = 1$ and ${\tilde{s}} = 1$.

Assume that (8) is true for ${\tilde{s}} = {\bar{a}}_{1} + 1$, ${\tilde{r}} = {\tilde{b}}_{1}$ and ${\tilde{s}} = {\bar{a}}_{1}$, ${\tilde{r}} = {\tilde{b}}_{1} + 1$, then it is follows that

$${  \oplus  }_{\nu =1}^{{{\bar{a}}}_{1}+1}{t}_{\nu }\left({  \oplus  }_{\upsilon =1}^{{{\tilde{b}}}_{1}}{n}_{\upsilon }{\mathfrak{L}}_{\upsilon \nu }\right)=\left(\begin{array}{c}\left[1-\prod_{\nu =1}^{{{\bar{a}}}_{1}+1}{\left(\prod_{\upsilon =1}^{{{\tilde{b}}}_{1}}{\left(1-{\rm N}_{{\mathfrak{L}}_{\upsilon \nu }}^{L+}\right)}^{{n}_{\upsilon }}\right)}^{{t}_{\nu }}, 1-\prod_{\nu =1}^{{{\bar{a}}}_{1}+1}{\left(\prod_{\upsilon =1}^{{{\tilde{b}}}_{1}}{\left(1-{\rm O}_{{\mathfrak{L}}_{\upsilon \nu }}^{U+}\right)}^{{n}_{\upsilon }}\right)}^{{t}_{\nu }}\right]\\ +\iota \left[1-\prod_{\nu =1}^{{{\bar{a}}}_{1}+1}{\left(\prod_{\upsilon =1}^{{{\tilde{b}}}_{1}}{\left(1-{\rm N}_{{\mathfrak{L}}_{\upsilon \nu }}^{L+}\right)}^{{n}_{\upsilon }}\right)}^{{t}_{\nu }}, 1-\prod_{\nu =1}^{{{\bar{a}}}_{1}+1}{\left(\prod_{\upsilon =1}^{{{\tilde{b}}}_{1}}{\left(1-{\rm O}_{{\mathfrak{L}}_{\upsilon \nu }}^{U+}\right)}^{{n}_{\upsilon }}\right)}^{{t}_{\nu }}\right],\\ \begin{array}{c}\left[-\prod_{\nu =1}^{{{\bar{a}}}_{1}+1}{\left(\prod_{\upsilon =1}^{{{\tilde{b}}}_{1}}{\left|{\rm N}_{{\mathfrak{L}}_{\upsilon \nu }}^{L-}\right|}^{{n}_{\upsilon }}\right)}^{{t}_{\nu }},-\prod_{\nu =1}^{{{\bar{a}}}_{1}+1}{\left(\prod_{\upsilon =1}^{{{\tilde{b}}}_{1}}{\left|{\rm O}_{{\mathfrak{L}}_{\upsilon \nu }}^{U-}\right|}^{{n}_{\upsilon }}\right)}^{{t}_{\nu }}\right]\\ +\iota \left[-\prod_{\nu =1}^{{{\bar{a}}}_{1}+1}{\left(\prod_{\upsilon =1}^{{{\tilde{b}}}_{1}}{\left|{\rm N}_{{\mathfrak{L}}_{\upsilon \nu }}^{L-}\right|}^{{n}_{\upsilon }}\right)}^{{t}_{\nu }},-\prod_{\nu =1}^{{{\bar{a}}}_{1}+1}{\left(\prod_{\upsilon =1}^{{{\tilde{b}}}_{1}}{\left|{\rm O}_{{\mathfrak{L}}_{\upsilon \nu }}^{U-}\right|}^{{n}_{\upsilon }}\right)}^{{t}_{\nu }}\right] \end{array}\end{array}\right)$$

Also,

$${  \oplus  }_{\nu =1}^{{{\bar{a}}}_{1}}{t}_{\nu }\left({  \oplus  }_{\upsilon =1}^{{{\tilde{b}}}_{1}+1}{n}_{\upsilon }{\mathfrak{L}}_{\upsilon \nu }\right)=\left(\begin{array}{c}\left[1-\prod_{\nu =1}^{{{\bar{a}}}_{1}}{\left(\prod_{\upsilon =1}^{{{\tilde{b}}}_{1}+1}{\left(1-{\rm N}_{{\mathfrak{L}}_{\upsilon \nu }}^{L+}\right)}^{{n}_{\upsilon }}\right)}^{{t}_{\nu }}, 1-\prod_{\nu =1}^{{{\bar{a}}}_{1}}{\left(\prod_{\upsilon =1}^{{{\tilde{b}}}_{1}+1}{\left(1-{\rm O}_{{\mathfrak{L}}_{\upsilon \nu }}^{U+}\right)}^{{n}_{\upsilon }}\right)}^{{t}_{\nu }}\right]\\ +\iota \left[1-\prod_{\nu =1}^{{{\bar{a}}}_{1}}{\left(\prod_{\upsilon =1}^{{{\tilde{b}}}_{1}+1}{\left(1-{\rm N}_{{\mathfrak{L}}_{\upsilon \nu }}^{L+}\right)}^{{n}_{\upsilon }}\right)}^{{t}_{\nu }}, 1-\prod_{\nu =1}^{{{\bar{a}}}_{1}}{\left(\prod_{\upsilon =1}^{{{\tilde{b}}}_{1}+1}{\left(1-{\rm O}_{{\mathfrak{L}}_{\upsilon \nu }}^{U+}\right)}^{{n}_{\upsilon }}\right)}^{{t}_{\nu }}\right],\\ \begin{array}{c}\left[-\prod_{\nu =1}^{{{\bar{a}}}_{1}}{\left(\prod_{\upsilon =1}^{{{\tilde{b}}}_{1}+1}{\left|{\rm N}_{{\mathfrak{L}}_{\upsilon \nu }}^{L-}\right|}^{{n}_{\upsilon }}\right)}^{{t}_{\nu }},-\prod_{\nu =1}^{{{\bar{a}}}_{1}}{\left(\prod_{\upsilon =1}^{{{\tilde{b}}}_{1}+1}{\left|{\rm O}_{{\mathfrak{L}}_{\upsilon \nu }}^{U-}\right|}^{{n}_{\upsilon }}\right)}^{{t}_{\nu }}\right]\\ +\iota \left[-\prod_{\nu =1}^{{{\bar{a}}}_{1}}{\left(\prod_{\upsilon =1}^{{{\tilde{b}}}_{1}+1}{\left|{\rm N}_{{\mathfrak{L}}_{\upsilon \nu }}^{L-}\right|}^{{n}_{\upsilon }}\right)}^{{t}_{\nu }},-\prod_{\nu =1}^{{{\bar{a}}}_{1}}{\left(\prod_{\upsilon =1}^{{{\tilde{b}}}_{1}+1}{\left|{\rm O}_{{\mathfrak{L}}_{\upsilon \nu }}^{U-}\right|}^{{n}_{\upsilon }}\right)}^{{t}_{\nu }}\right] \end{array}\end{array}\right)$$

Now for ${\tilde{s}} = {\bar{a}}_{1} + 1$, ${\tilde{r}} = {\tilde{b}}_{1} + 1$, we got

$${  \oplus  }_{\nu =1}^{{{\bar{a}}}_{1}+1}{t}_{\nu }\left({  \oplus  }_{\upsilon =1}^{{{\tilde{b}}}_{1}+1}{n}_{\upsilon }{\mathfrak{L}}_{\upsilon \nu }\right)$$

$$={  \oplus  }_{\nu =1}^{{{\bar{a}}}_{1}+1}{t}_{\nu }\left({  \oplus  }_{\upsilon =1}^{{{\tilde{b}}}_{1}}{n}_{\upsilon }{\mathfrak{L}}_{\upsilon \nu }  \oplus  {n}_{{{\tilde{b}}}_{1}+1}{\mathfrak{L}}_{\left({{\tilde{b}}}_{1}+1\right)\nu }\right)={  \oplus  }_{\nu =1}^{{{\bar{a}}}_{1}+1}{t}_{\nu }{  \oplus  }_{\upsilon =1}^{{{\tilde{b}}}_{1}}{n}_{\upsilon }{\mathfrak{L}}_{\upsilon \nu }{  \oplus  }_{\nu =1}^{{{\bar{a}}}_{1}+1}{t}_{\nu }{n}_{{{\tilde{b}}}_{1}+1}{\mathfrak{L}}_{\left({{\tilde{b}}}_{1}+1\right)\nu }$$

$$=\left(\begin{array}{c}\left[\begin{array}{c}1-\prod_{\nu =1}^{{{\bar{a}}}_{1}+1}{\left(\prod_{\upsilon =1}^{{{\tilde{b}}}_{1}}{\left(1-{\rm N}_{{\mathfrak{L}}_{\upsilon \nu }}^{L+}\right)}^{{n}_{\upsilon }}\right)}^{{t}_{\nu }}  \oplus  1-\prod_{\nu =1}^{{{\bar{a}}}_{1}+1}{\left({\left(1-{\rm N}_{{\mathfrak{L}}_{\left({{\tilde{b}}}_{1}+1\right)\nu }}^{L+}\right)}^{{n}_{\left({{\tilde{b}}}_{1}+1\right)}}\right)}^{{t}_{\nu }},\\ 1-\prod_{\nu =1}^{{{\bar{a}}}_{1}+1}{\left(\prod_{\upsilon =1}^{{{\tilde{b}}}_{1}}{\left(1-{\rm O}_{{\mathfrak{L}}_{\upsilon \nu }}^{U+}\right)}^{{n}_{\upsilon }}\right)}^{{t}_{\nu }}  \oplus  1-\prod_{\nu =1}^{{{\bar{a}}}_{1}+1}{\left({\left(1-{\rm O}_{{\mathfrak{L}}_{\left({{\tilde{b}}}_{1}+1\right)\nu }}^{U+}\right)}^{{n}_{\left({{\tilde{b}}}_{1}+1\right)}}\right)}^{{t}_{\nu }}\end{array}\right]\\ +\iota \left[\begin{array}{c}1-\prod_{\nu =1}^{{{\bar{a}}}_{1}+1}{\left(\prod_{\upsilon =1}^{{{\tilde{b}}}_{1}}{\left(1-{\rm N}_{{\mathfrak{L}}_{\upsilon \nu }}^{L+}\right)}^{{n}_{\upsilon }}\right)}^{{t}_{\nu }}  \oplus  1-\prod_{\nu =1}^{{{\bar{a}}}_{1}+1}{\left({\left(1-{\rm N}_{{\mathfrak{L}}_{\left({{\tilde{b}}}_{1}+1\right)\nu }}^{L+}\right)}^{{n}_{\left({{\tilde{b}}}_{1}+1\right)}}\right)}^{{t}_{\nu }},\\ 1-\prod_{\nu =1}^{{{\bar{a}}}_{1}+1}{\left(\prod_{\upsilon =1}^{{{\tilde{b}}}_{1}}{\left(1-{\rm O}_{{\mathfrak{L}}_{\upsilon \nu }}^{U+}\right)}^{{n}_{\upsilon }}\right)}^{{t}_{\nu }}  \oplus  1-\prod_{\nu =1}^{{{\bar{a}}}_{1}+1}{\left({\left(1-{\rm O}_{{\mathfrak{L}}_{\left({{\tilde{b}}}_{1}+1\right)\nu }}^{U+}\right)}^{{n}_{\left({{\tilde{b}}}_{1}+1\right)}}\right)}^{{t}_{\nu }}\end{array}\right],\\ \begin{array}{c}\left[\begin{array}{c}-\prod_{\nu =1}^{{{\bar{a}}}_{1}+1}{\left(\prod_{\upsilon =1}^{{{\tilde{b}}}_{1}}{\left|{\rm N}_{{\mathfrak{L}}_{\upsilon \nu }}^{L-}\right|}^{{n}_{\upsilon }}\right)}^{{t}_{\nu }}  \oplus  -\prod_{\nu =1}^{{{\bar{a}}}_{1}+1}{\left({\left|{\rm N}_{{\mathfrak{L}}_{\left({{\tilde{b}}}_{1}+1\right)\nu }}^{L-}\right|}^{{n}_{\left({{\tilde{b}}}_{1}+1\right)}}\right)}^{{t}_{\nu }},\\ -\prod_{\nu =1}^{{{\bar{a}}}_{1}+1}{\left(\prod_{\upsilon =1}^{{{\tilde{b}}}_{1}}{\left|{\rm O}_{{\mathfrak{L}}_{\upsilon \nu }}^{U-}\right|}^{{n}_{\upsilon }}\right)}^{{t}_{\nu }}  \oplus  -\prod_{\nu =1}^{{{\bar{a}}}_{1}+1}{\left({\left|{\rm O}_{{\mathfrak{L}}_{\left({{\tilde{b}}}_{1}+1\right)\nu }}^{U-}\right|}^{{n}_{\left({{\tilde{b}}}_{1}+1\right)}}\right)}^{{t}_{\nu }}\end{array}\right]\\ +\iota \left[\begin{array}{c}-\prod_{\nu =1}^{{{\bar{a}}}_{1}+1}{\left(\prod_{\upsilon =1}^{{{\tilde{b}}}_{1}}{\left|{\rm N}_{{\mathfrak{L}}_{\upsilon \nu }}^{L-}\right|}^{{n}_{\upsilon }}\right)}^{{t}_{\nu }}  \oplus  -\prod_{\nu =1}^{{{\bar{a}}}_{1}+1}{\left({\left|{\rm N}_{{\mathfrak{L}}_{\left({{\tilde{b}}}_{1}+1\right)\nu }}^{L-}\right|}^{{n}_{\left({{\tilde{b}}}_{1}+1\right)}}\right)}^{{t}_{\nu }},\\ -\prod_{\nu =1}^{{{\bar{a}}}_{1}+1}{\left(\prod_{\upsilon =1}^{{{\tilde{b}}}_{1}}{\left|{\rm O}_{{\mathfrak{L}}_{\upsilon \nu }}^{U-}\right|}^{{n}_{\upsilon }}\right)}^{{t}_{\nu }}  \oplus  -\prod_{\nu =1}^{{{\bar{a}}}_{1}+1}{\left({\left|{\rm O}_{{\mathfrak{L}}_{\left({{\tilde{b}}}_{1}+1\right)\nu }}^{U-}\right|}^{{n}_{\left({{\tilde{b}}}_{1}+1\right)}}\right)}^{{t}_{\nu }}\end{array}\right] \end{array}\end{array}\right)$$

Hence, (8) is true $${\tilde{s}} = {\bar{a}}_{1} + 1$$, $${\tilde{r}} = {\tilde{b}}_{1} + 1$$, consequently for induction the results are satisfied $$\forall {\tilde{r}} ,{\tilde{s}} \ge$$$$1$$.

Now we desire to explain the following properties by the usage the operator IVBCFSWAA.

#### Theorem 2

(Idempotency): Suppose

$${\mathfrak{L}}_{\upsilon \nu } = \left( {{\rm E}_{{{\mathfrak{L}}_{\upsilon \nu } }}^{ + } , {\rm E}_{{{\mathfrak{L}}_{\upsilon \nu } }}^{ - } } \right) = \left( {\begin{array}{*{20}c} {\left[ {{\rm N}_{{{\mathfrak{L}}_{\upsilon \nu } }}^{L + } , {\rm O}_{{{\mathfrak{L}}_{\upsilon \nu } }}^{U + } } \right] + \iota \left[ {{\rm N}_{{{\mathfrak{L}}_{\upsilon \nu } }}^{L + } , {\rm O}_{{{\mathfrak{L}}_{\upsilon \nu } }}^{U + } } \right], } \\ {\left[ {{\rm N}_{{{\mathfrak{L}}_{\upsilon \nu } }}^{L - } , {\rm O}_{{{\mathfrak{L}}_{\upsilon \nu } }}^{U - } } \right] + \iota \left[ {{\rm N}_{{{\mathfrak{L}}_{\upsilon \nu } }}^{L - } , {\rm O}_{{{\mathfrak{L}}_{\upsilon \nu } }}^{U - } } \right]} \\ \end{array} } \right)\left( {\upsilon = 1, 2, \ldots , {\tilde{r}} ; \nu = 1, 2, \ldots , {\tilde{s}} } \right)$$

be the assortment of IVBCFSNs, then using the IVBCFSWAA operator is also a IVBCFSNs that are all equal, i.e., ${\mathfrak{L}}_{\upsilon \nu }=\mathfrak{L} \forall \upsilon , \nu$ then

9 $$ IVBCFSWAA\left( {{\mathfrak{L}}_{11} , {\mathfrak{L}}_{12} , \ldots , {\mathfrak{L}}_{{{\tilde{r}} {\tilde{s}} }} } \right) = {\mathfrak{L}} $$

#### Proof

Since $${\mathfrak{L}}_{\upsilon \nu }=\mathfrak{L}=\left({\rm E}_{\mathfrak{L}}^{+}, {\rm E}_{\mathfrak{L}}^{-}\right)=\left(\begin{array}{c}\left[{\rm N}_{\mathfrak{L}}^{L+}, {\rm O}_{\mathfrak{L}}^{U+}\right]+\iota \left[{\rm N}_{\mathfrak{L}}^{L+}, {\rm O}_{\mathfrak{L}}^{U+}\right], \\ \left[{\rm N}_{\mathfrak{L}}^{L-}, {\rm O}_{\mathfrak{L}}^{U-}\right]+\iota \left[{\rm N}_{\mathfrak{L}}^{L-}, {\rm O}_{\mathfrak{L}}^{U-}\right]\end{array}\right) \forall \upsilon , \nu $$ then,

$$  \begin{gathered}   IVBCFSWAA\left( { \mathfrak{L} _{{11}} ,~ \mathfrak{L} _{{12}} ,~ \ldots ,~ \mathfrak{L} _{{\tilde{r}{\tilde{s}} }} } \right) \hfill \\    = \left( \begin{gathered}   \left[ {1 - \mathop \prod \limits_{{\nu  = 1}}^{{{\tilde{s}} }} \left( {\mathop \prod \limits_{{\upsilon  = 1}}^{{{\tilde{r}} }} \left( {1 - {\rm N}_{ \mathfrak{L} }^{{L + }} } \right)^{{{n} _{\upsilon } }} } \right)^{{{t} _{\nu } }} ,~1 - \mathop \prod \limits_{{\nu  = 1}}^{{{\tilde{s}} }} \left( {\mathop \prod \limits_{{\upsilon  = 1}}^{{{\tilde{r}} }} \left( {1 - {\rm O}_{ \mathfrak{L} }^{{U + }} } \right)^{{{n} _{\upsilon } }} } \right)^{{{t} _{\nu } }} } \right] \hfill \\    + \iota \left[ {1 - \mathop \prod \limits_{{\nu  = 1}}^{{{\tilde{s}} }} \left( {\mathop \prod \limits_{{\upsilon  = 1}}^{{{\tilde{r}} }} \left( {1 - {\rm N}_{ \mathfrak{L} }^{{L + }} } \right)^{{{n} _{\upsilon } }} } \right)^{{{t} _{\nu } }} ,~1 - \mathop \prod \limits_{{\nu  = 1}}^{{{\tilde{s}} }} \left( {\mathop \prod \limits_{{\upsilon  = 1}}^{{{\tilde{r}} }} \left( {1 - {\rm O}_{ \mathfrak{L} }^{{U + }} } \right)^{{{n} _{\upsilon } }} } \right)^{{{t} _{\nu } }} } \right], \hfill \\   \left[ { - \mathop \prod \limits_{{\nu  = 1}}^{{{\tilde{s}} }} \left( {\mathop \prod \limits_{\upsilon }^{{{\tilde{r}} }} \left| {{\rm N}_{ \mathfrak{L} }^{{L - }} } \right|^{{{n} _{\upsilon } }} } \right)^{{{t} _{\nu } }} , - \mathop \prod \limits_{{\nu  = 1}}^{{{\tilde{s}} }} \left( {\mathop \prod \limits_{\upsilon }^{{{\tilde{r}} }} \left| {{\rm O}_{ \mathfrak{L} }^{{U - }} } \right|^{{{n} _{\upsilon } }} } \right)^{{{t} _{\nu } }} } \right] \hfill \\    + \iota \left[ { - \mathop \prod \limits_{{\nu  = 1}}^{{{\tilde{s}} }} \left( {\mathop \prod \limits_{\upsilon }^{{{\tilde{r}} }} \left| {{\rm N}_{ \mathfrak{L} }^{{L - }} } \right|^{{{n} _{\upsilon } }} } \right)^{{{t} _{\nu } }} , - \mathop \prod \limits_{{\nu  = 1}}^{{{\tilde{s}} }} \left( {\mathop \prod \limits_{\upsilon }^{{{\tilde{r}} }} \left| {{\rm O}_{ \mathfrak{L} }^{{U - }} } \right|^{{{n} _{\upsilon } }} } \right)^{{{t} _{\nu } }} } \right] \hfill \\  \end{gathered}  \right) \hfill \\    = \left( \begin{gathered}   \left[ {1 - \left( {\left( {1 - {\rm N}_{ \mathfrak{L} }^{{L + }} } \right)^{{\mathop \sum \nolimits_{{\upsilon  = 1}}^{{{\tilde{r}} }} {n} _{\upsilon } }} } \right)^{{\mathop \sum \nolimits_{{\nu  = 1}}^{{{\tilde{s}} }} {t} _{\nu } }} ,~1 - \left( {\left( {1 - {\rm O}_{ \mathfrak{L} }^{{U + }} } \right)^{{\mathop \sum \nolimits_{{\upsilon  = 1}}^{{{\tilde{r}} }} {n} _{\upsilon } }} } \right)^{{\mathop \sum \nolimits_{{\nu  = 1}}^{{{\tilde{s}} }} {t} _{\nu } }} } \right] +  \hfill \\   \iota \left[ {1 - \left( {\left( {1 - {\rm N}_{ \mathfrak{L} }^{{L + }} } \right)^{{\mathop \sum \nolimits_{{\upsilon  = 1}}^{{{\tilde{r}} }} {n} _{\upsilon } }} } \right)^{{\mathop \sum \nolimits_{{\nu  = 1}}^{{{\tilde{s}} }} {t} _{\nu } }} ,~1 - \left( {\left( {1 - {\rm O}_{ \mathfrak{L} }^{{U + }} } \right)^{{\mathop \sum \nolimits_{{\upsilon  = 1}}^{{{\tilde{r}} }} {n} _{\upsilon } }} } \right)^{{\mathop \sum \nolimits_{{\nu  = 1}}^{{{\tilde{s}} }} {t} _{\nu } }} } \right], \hfill \\   \left[ {\left( {\left| {{\rm N}_{ \mathfrak{L} }^{{L - }} } \right|^{{\mathop \sum \nolimits_{{\upsilon  = 1}}^{{{\tilde{r}} }} {n} _{\upsilon } }} } \right)^{{\mathop \sum \nolimits_{{\nu  = 1}}^{{{\tilde{s}} }} T_{\nu } }} ,~\left( {\left| {{\rm O}_{ \mathfrak{L} }^{{U - }} } \right|^{{\mathop \sum \nolimits_{{\upsilon  = 1}}^{{{\tilde{r}} }} {n} _{\upsilon } }} } \right)^{{\mathop \sum \nolimits_{{\nu  = 1}}^{{{\tilde{s}} }} {t} _{\nu } }} } \right] +  \hfill \\   \iota \left[ {\left( {\left| {{\rm N}_{ \mathfrak{L} }^{{L - }} } \right|^{{\mathop \sum \nolimits_{{\upsilon  = 1}}^{{{\tilde{r}} }} {n} _{\upsilon } }} } \right)^{{\mathop \sum \nolimits_{{\nu  = 1}}^{{{\tilde{s}} }} {t} _{\nu } }} ,~\left( {\left| {{\rm O}_{ \mathfrak{L} }^{{U - }} } \right|^{{\mathop \sum \nolimits_{{\upsilon  = 1}}^{{{\tilde{r}} }} {n} _{\upsilon } }} } \right)^{{\mathop \sum \nolimits_{{\nu  = 1}}^{{{\tilde{s}} }} {t} _{\nu } }} } \right] \hfill \\  \end{gathered}  \right) \hfill \\    = \left( {\begin{array}{*{20}c}    {\left[ {{\rm N}_{ \mathfrak{L} }^{{L + }} ,~{\rm O}_{ \mathfrak{L} }^{{U + }} } \right] + }  \\    {\iota \left[ {{\rm N}_{ \mathfrak{L} }^{{L + }} ,~{\rm O}_{ \mathfrak{L} }^{{U + }} } \right],}  \\    {\begin{array}{*{20}c}    {\left[ {{\rm N}_{ \mathfrak{L} }^{{L - }} ,~{\rm O}_{ \mathfrak{L} }^{{U - }} } \right] + }  \\    {\iota \left[ {{\rm N}_{ \mathfrak{L} }^{{L - }} ,~{\rm O}_{ \mathfrak{L} }^{{U - }} } \right]}  \\   \end{array} }  \\   \end{array} } \right) = \left( {{\rm E}_{ \mathfrak{L} }^{ + } ,~{\rm E}_{ \mathfrak{L} }^{ - } } \right) \hfill \\  \end{gathered}   $$

#### Theorem 3

(Boundedness): Suppose

$${\mathfrak{L}}_{\upsilon \nu } = \left( {{\rm E}_{{{\mathfrak{L}}_{\upsilon \nu } }}^{ + } , {\rm E}_{{{\mathfrak{L}}_{\upsilon \nu } }}^{ - } } \right) = \left( {\begin{array}{*{20}c} {\left[ {{\rm N}_{{{\mathfrak{L}}_{\upsilon \nu } }}^{L + } , {\rm O}_{{{\mathfrak{L}}_{\upsilon \nu } }}^{U + } } \right] + \iota \left[ {{\rm N}_{{{\mathfrak{L}}_{\upsilon \nu } }}^{L + } , {\rm O}_{{{\mathfrak{L}}_{\upsilon \nu } }}^{U + } } \right], } \\ {\left[ {{\rm N}_{{{\mathfrak{L}}_{\upsilon \nu } }}^{L - } , {\rm O}_{{{\mathfrak{L}}_{\upsilon \nu } }}^{U - } } \right] + \iota \left[ {{\rm N}_{{{\mathfrak{L}}_{\upsilon \nu } }}^{L - } , {\rm O}_{{{\mathfrak{L}}_{\upsilon \nu } }}^{U - } } \right]} \\ \end{array} } \right)\left( {\upsilon = 1, 2, \ldots , {\tilde{r}} ; \nu = 1, 2, \ldots , {\tilde{s}} } \right)$$

be the assortment of IVBCFSNs.

$${\mathfrak{L}}_{\upsilon \nu }^{-}=\left(\underset{\nu }{{\text{min}}}\,\underset{\upsilon }{{\text{min}}}\,\left\{{\rm E}_{{\mathfrak{L}}_{\upsilon \nu }}^{+}\right\}, \underset{\nu }{{\text{max}}}\,\underset{\upsilon }{{\text{max}}}\left\{{\rm E}_{{\mathfrak{L}}_{\upsilon \nu }}^{-}\right\}\right)=\left(\begin{array}{c}\left[\underset{\nu }{{\text{min}}}\,\underset{\upsilon }{{\text{min}}}\,\left\{{\rm N}_{{\mathfrak{L}}_{\upsilon \nu }}^{L+}\right\}, \underset{\nu }{{\text{min}}}\,\underset{\upsilon }{{\text{min}}}\,\left\{{\rm O}_{{\mathfrak{L}}_{\upsilon \nu }}^{U+}\right\}\right]\\ +\iota \left[\underset{\nu }{{\text{min}}}\,\underset{\upsilon }{{\text{min}}}\,\left\{{\rm N}_{{\mathfrak{L}}_{\upsilon \nu }}^{L+}\right\}, \underset{\nu }{{\text{min}}}\,\underset{\upsilon }{{\text{min}}}\,\left\{{\rm O}_{{\mathfrak{L}}_{\upsilon \nu }}^{U+}\right\}\right], \\ \left[\underset{\nu }{{\text{max}}}\,\underset{\upsilon }{{\text{max}}}\left\{{\rm N}_{{\mathfrak{L}}_{\upsilon \nu }}^{L-}\right\}, \underset{\nu }{{\text{max}}}\,\underset{\upsilon }{{\text{max}}}\left\{{\rm O}_{{\mathfrak{L}}_{\upsilon \nu }}^{U-}\right\}\right]+\\ \iota \left[\underset{\nu }{{\text{max}}}\,\underset{\upsilon }{{\text{max}}}\left\{{\rm N}_{{\mathfrak{L}}_{\upsilon \nu }}^{L-}\right\}, \underset{\nu }{{\text{max}}}\,\underset{\upsilon }{{\text{max}}}\left\{{\rm O}_{{\mathfrak{L}}_{\upsilon \nu }}^{U-}\right\}\right]\end{array}\right)$$

And $${\mathfrak{L}}_{\upsilon \nu }^{+}=\left(\underset{\nu }{{\text{max}}}\,\underset{\upsilon }{{\text{max}}}\left\{{\rm E}_{{\mathfrak{L}}_{\upsilon \nu }}^{+}\right\}, \underset{\nu }{{\text{min}}}\,\underset{\upsilon }{{\text{min}}}\,\left\{{\rm E}_{{\mathfrak{L}}_{\upsilon \nu }}^{-}\right\}\right)=\left(\begin{array}{c}\left[\underset{\nu }{{\text{max}}}\,\underset{\upsilon }{{\text{max}}}\left\{{\rm N}_{{\mathfrak{L}}_{\upsilon \nu }}^{L+}\right\}, \underset{\mathit{\nu }}{{\text{max}}}\underset{\upsilon }{{\text{max}}}\left\{{\rm O}_{{\mathfrak{L}}_{\upsilon \nu }}^{U+}\right\}\right]+\\ \iota \left[\underset{\nu }{{\text{max}}}\underset{\upsilon }{{\text{max}}}\left\{{\rm N}_{{\mathfrak{L}}_{\upsilon \nu }}^{L+}\right\}, \underset{\mathit{\nu }}{{\text{max}}}\underset{\upsilon }{{\text{max}}}\left\{{\rm O}_{{\mathfrak{L}}_{\upsilon \nu }}^{U+}\right\}\right], \\ \left[\underset{\nu }{{\text{min}}}\,\underset{\upsilon }{{\text{min}}}\,\left\{{\rm N}_{{\mathfrak{L}}_{\upsilon \nu }}^{L-}\right\}, \underset{\nu }{{\text{min}}}\,\underset{\upsilon }{{\text{min}}}\,\left\{{\rm O}_{{\mathfrak{L}}_{\upsilon \nu }}^{U-}\right\}\right]+\\ \iota \left[\underset{\nu }{{\text{min}}}\,\underset{\upsilon }{{\text{min}}}\,\left\{{\rm N}_{{\mathfrak{L}}_{\upsilon \nu }}^{L-}\right\}, \underset{\nu }{{\text{min}}}\,\underset{\upsilon }{{\text{min}}}\,\left\{{\rm O}_{{\mathfrak{L}}_{\upsilon \nu }}^{U-}\right\}\right]\end{array}\right)$$

Then,

10 $$ {\mathfrak{L}}_{\upsilon \nu }^{ - } \le IVBCFSWAA\left( {{\mathfrak{L}}_{11} , {\mathfrak{L}}_{12} , \ldots , {\mathfrak{L}}_{{{\tilde{r}} {\tilde{s}} }} } \right) \le {\mathfrak{L}}_{\upsilon \nu }^{ + } $$

#### Proof

Since, ${\mathfrak{L}}_{\upsilon \nu }=\left({\rm E}_{{\mathfrak{L}}_{\upsilon \nu }}^{+}, {\rm E}_{{\mathfrak{L}}_{\upsilon \nu }}^{-}\right)$ is a IVBCFSN then

11 $$ \begin{aligned} & \left[ {\mathop {\min }\limits_{\nu } \mathop {\min }\limits_{\upsilon } \left\{ {{\rm N}_{{{\mathfrak{L}}_{\upsilon \nu } }}^{L + } } \right\}, \mathop {\min }\limits_{\nu } \mathop {\min }\limits_{\upsilon } \left\{ {{\rm O}_{{{\mathfrak{L}}_{\upsilon \nu } }}^{U + } } \right\}} \right] + \iota \left[ {\mathop {\min }\limits_{\nu } \mathop {\min }\limits_{\upsilon } \left\{ {{\rm N}_{{{\mathfrak{L}}_{\upsilon \nu } }}^{L + } } \right\}, \mathop {\min }\limits_{\nu } \mathop {\min }\limits_{\upsilon } \left\{ {{\rm O}_{{{\mathfrak{L}}_{\upsilon \nu } }}^{U + } } \right\}} \right] \hfill \\ & \quad  \le \left[ {{\rm N}_{{{\mathfrak{L}}_{\upsilon \nu } }}^{L + } , {\rm O}_{{{\mathfrak{L}}_{\upsilon \nu } }}^{U + } } \right] + \iota \left[ {{\rm N}_{{{\mathfrak{L}}_{\upsilon \nu } }}^{L + } , {\rm O}_{{{\mathfrak{L}}_{\upsilon \nu } }}^{U + } } \right] \\ & \quad  \le  \left[ {\mathop {\max }\limits_{\nu } \mathop {\max }\limits_{\upsilon } \left\{ {{\rm N}_{{{\mathfrak{L}}_{\upsilon \nu } }}^{L + } } \right\}, \mathop {\max }\limits_{\nu } \mathop {\max }\limits_{\upsilon } \left\{ {{\rm O}_{{{\mathfrak{L}}_{\upsilon \nu } }}^{U + } } \right\}} \right] + \iota \left[ {\mathop {\max }\limits_{\nu } \mathop {\max }\limits_{\upsilon } \left\{ {{\rm N}_{{{\mathfrak{L}}_{\upsilon \nu } }}^{L + } } \right\}, \mathop {\max }\limits_{\nu } \mathop {\max }\limits_{\upsilon } \left\{ {{\rm O}_{{{\mathfrak{L}}_{\upsilon \nu } }}^{U + } } \right\}} \right] \hfill \\ \end{aligned} $$

Which implies that

$$  \begin{aligned}   & \left[ {1 - \mathop {\min }\limits_{\nu } \mathop {\min }\limits_{\upsilon } \left\{ {{\rm N}_{{{\mathfrak{L}}_{{\upsilon \nu }} }}^{{L + }} } \right\},~1 - \mathop {\min }\limits_{\nu } \mathop {\min }\limits_{\upsilon } \left\{ {{\rm O}_{{{\mathfrak{L}}_{{\upsilon \nu }} }}^{{U + }} } \right\}} \right] + \iota \left[ {1 - \mathop {\min }\limits_{\nu } \mathop {\min }\limits_{\upsilon } \left\{ {{\rm N}_{{{\mathfrak{L}}_{{\upsilon \nu }} }}^{{L + }} } \right\},~1 - \mathop {\min }\limits_{\nu } \mathop {\min }\limits_{\upsilon } \left\{ {{\rm O}_{{{\mathfrak{L}}_{{\upsilon \nu }} }}^{{U + }} } \right\}} \right] \hfill \\  &  \quad \le \left[ {1 - {\rm N}_{{{\mathfrak{L}}_{{\upsilon \nu }} }}^{{L + }} ,~1 - {\rm O}_{{{\mathfrak{L}}_{{\upsilon \nu }} }}^{{U + }} } \right] + \iota \left[ {1 - {\rm N}_{{{\mathfrak{L}}_{{\upsilon \nu }} }}^{{L + }} ,~1 - {\rm O}_{{{\mathfrak{L}}_{{\upsilon \nu }} }}^{{U + }} } \right] \\ & \quad \le   \left[ {1 - \mathop {\max }\limits_{\nu } \mathop {\max }\limits_{\upsilon } \left\{ {{\rm N}_{{{\mathfrak{L}}_{{\upsilon \nu }} }}^{{L + }} } \right\},~1 - \mathop {\max }\limits_{{\nu ~}} \mathop {\max }\limits_{\upsilon } \left\{ {{\rm O}_{{{\mathfrak{L}}_{{\upsilon \nu }} }}^{{U + }} } \right\}} \right] + \iota \left[ {1 - \mathop {\max }\limits_{\nu } \mathop {\max }\limits_{\upsilon } \left\{ {{\rm N}_{{{\mathfrak{L}}_{{\upsilon \nu }} }}^{{L + }} } \right\},~\mathop {1 - \max }\limits_{{\nu ~}} \mathop {\max }\limits_{\upsilon } \left\{ {{\rm O}_{{{\mathfrak{L}}_{{\upsilon \nu }} }}^{{U + }} } \right\}} \right] \\ & \quad    \Leftrightarrow \left[ {\left( {1 - \mathop {\min }\limits_{\nu } \mathop {\min }\limits_{\upsilon } \left\{ {{\rm N}_{{{\mathfrak{L}}_{{\upsilon \nu }} }}^{{L + }} } \right\}} \right)^{{{n} _{\upsilon } }} ,~\left( {1 - \mathop {\min }\limits_{\nu } \mathop {\min }\limits_{\upsilon } \left\{ {{\rm O}_{{{\mathfrak{L}}_{{\upsilon \nu }} }}^{{U + }} } \right\}} \right)^{{{n} _{\upsilon } }} } \right] + \iota \left[ {\left( {1 - \mathop {\min }\limits_{\nu } \mathop {\min }\limits_{\upsilon } \left\{ {{\rm N}_{{{\mathfrak{L}}_{{\upsilon \nu }} }}^{{L + }} } \right\}} \right)^{{{n} _{\upsilon } }} ,~\left( {1 - \mathop {\min }\limits_{\nu } \mathop {\min }\limits_{\upsilon } \left\{ {{\rm O}_{{{\mathfrak{L}}_{{\upsilon \nu }} }}^{{U + }} } \right\}} \right)^{{{n} _{\upsilon } }} } \right] \\ & \quad    \le \left[ {1 - {\rm N}_{{{\mathfrak{L}}_{{\upsilon \nu }} }}^{{L + }} ,~1 - {\rm O}_{{{\mathfrak{L}}_{{\upsilon \nu }} }}^{{U + }} } \right] + \iota \left[ {1 - {\rm N}_{{{\mathfrak{L}}_{{\upsilon \nu }} }}^{{L + }} ,~1 - {\rm O}_{{{\mathfrak{L}}_{{\upsilon \nu }} }}^{{U + }} } \right] \\ & \quad \le     \left[ {\left( {1 - \mathop {\max }\limits_{\nu } \mathop {\max }\limits_{\upsilon } \left\{ {{\rm N}_{{{\mathfrak{L}}_{{\upsilon \nu }} }}^{{L + }} } \right\}} \right)^{{{n} _{\upsilon } }} ,~\left( {1 - \mathop {\max }\limits_{{\nu ~}} \mathop {\max }\limits_{\upsilon } \left\{ {{\rm O}_{{{\mathfrak{L}}_{{\upsilon \nu }} }}^{{U + }} } \right\}} \right)^{{{n} _{\upsilon } }} } \right] + \iota \left[ {\left( {1 - \mathop {\max }\limits_{\nu } \mathop {\max }\limits_{\upsilon } \left\{ {{\rm N}_{{{\mathfrak{L}}_{{\upsilon \nu }} }}^{{L + }} } \right\}} \right)^{{{n} _{\upsilon } }} ,~\left( {1 - \mathop {\max }\limits_{{\nu ~}} \mathop {\max }\limits_{\upsilon } \left\{ {{\rm O}_{{{\mathfrak{L}}_{{\upsilon \nu }} }}^{{U + }} } \right\}} \right)^{{{n} _{\upsilon } }} } \right] \hfill \\  & \quad   \Leftrightarrow \left[ {1 - \mathop {\min }\limits_{\nu } \mathop {\min }\limits_{\upsilon } \left\{ {{\rm N}_{{{\mathfrak{L}}_{{\upsilon \nu }} }}^{{L + }} } \right\},~1 - \mathop {\min }\limits_{\nu } \mathop {\min }\limits_{\upsilon } \left\{ {{\rm O}_{{{\mathfrak{L}}_{{\upsilon \nu }} }}^{{U + }} } \right\}} \right] + \iota \left[ {1 - \mathop {\min }\limits_{\nu } \mathop {\min }\limits_{\upsilon } \left\{ {{\rm N}_{{{\mathfrak{L}}_{{\upsilon \nu }} }}^{{L + }} } \right\},~1 - \mathop {\min }\limits_{\nu } \mathop {\min }\limits_{\upsilon } \left\{ {{\rm O}_{{{\mathfrak{L}}_{{\upsilon \nu }} }}^{{U + }} } \right\}} \right] \\ & \quad   \le \left[ {\mathop \prod \limits_{{\upsilon  = 1}}^{{{\tilde{r}} }} \left( {1 - {\rm N}_{{{\mathfrak{L}}_{{\upsilon \nu }} }}^{{L + }} } \right)^{{{n} _{\upsilon } }} ,~\mathop \prod \limits_{{\upsilon  = 1}}^{{{\tilde{r}} }} \left( {1 - {\rm O}_{{{\mathfrak{L}}_{{\upsilon \nu }} }}^{{U + }} } \right)^{{{n} _{\upsilon } }} } \right] + \iota \left[ {\mathop \prod \limits_{{\upsilon  = 1}}^{{{\tilde{r}} }} \left( {1 - {\rm N}_{{{\mathfrak{L}}_{{\upsilon \nu }} }}^{{L + }} } \right)^{{{n} _{\upsilon } }} ,~\mathop \prod \limits_{{\upsilon  = 1}}^{{{\tilde{r}} }} \left( {1 - {\rm O}_{{{\mathfrak{L}}_{{\upsilon \nu }} }}^{{U + }} } \right)^{{{n} _{\upsilon } }} } \right] \\ & \quad \le  \left[ {1 - \mathop {\max }\limits_{\nu } \mathop {\max }\limits_{\upsilon } \left\{ {{\rm N}_{{{\mathfrak{L}}_{{\upsilon \nu }} }}^{{L + }} } \right\},~1 - \mathop {\max }\limits_{{\nu ~}} \mathop {\max }\limits_{\upsilon } \left\{ {{\rm O}_{{{\mathfrak{L}}_{{\upsilon \nu }} }}^{{U + }} } \right\}} \right] + \iota \left[ {1 - \mathop {\max }\limits_{\nu } \mathop {\max }\limits_{\upsilon } \left\{ {{\rm N}_{{{\mathfrak{L}}_{{\upsilon \nu }} }}^{{L + }} } \right\},~\mathop {1 - \max }\limits_{{\nu ~}} \mathop {\max }\limits_{\upsilon } \left\{ {{\rm O}_{{{\mathfrak{L}}_{{\upsilon \nu }} }}^{{U + }} } \right\}} \right] \\ & \quad    \Leftrightarrow \left[ {\left( {1 - \mathop {\min }\limits_{\nu } \mathop {\min }\limits_{\upsilon } \left\{ {{\rm N}_{{{\mathfrak{L}}_{{\upsilon \nu }} }}^{{L + }} } \right\}} \right),~\left( {1 - \mathop {\min }\limits_{\nu } \mathop {\min }\limits_{\upsilon } \left\{ {{\rm O}_{{{\mathfrak{L}}_{{\upsilon \nu }} }}^{{U + }} } \right\}} \right)} \right]  \\ & \quad +    \iota \left[ {\left( {1 - \mathop {\max }\limits_{\nu } \mathop {\max }\limits_{\upsilon } \left\{ {{\rm N}_{{{\mathfrak{L}}_{{\upsilon \nu }} }}^{{L + }} } \right\}} \right),~\left( {1 - \mathop {\max }\limits_{{\nu ~}} \mathop {\max }\limits_{\upsilon } \left\{ {{\rm O}_{{{\mathfrak{L}}_{{\upsilon \nu }} }}^{{U + }} } \right\}} \right)} \right] \\ & \quad   \le \left[ {\mathop \prod \limits_{{\upsilon  = 1}}^{{{\tilde{r}} }} \left( {1 - {\rm N}_{{{\mathfrak{L}}_{{\upsilon \nu }} }}^{{L + }} } \right)^{{{n} _{\upsilon } }} ,~\mathop \prod \limits_{{\upsilon  = 1}}^{{{\tilde{r}} }} \left( {1 - {\rm O}_{{{\mathfrak{L}}_{{\upsilon \nu }} }}^{{U + }} } \right)^{{{n} _{\upsilon } }} } \right] + \iota \left[ {\mathop \prod \limits_{{\upsilon  = 1}}^{{{\tilde{r}} }} \left( {1 - {\rm N}_{{{\mathfrak{L}}_{{\upsilon \nu }} }}^{{L + }} } \right)^{{{n} _{\upsilon } }} ,~\mathop \prod \limits_{{\upsilon  = 1}}^{{{\tilde{r}} }} \left( {1 - {\rm O}_{{{\mathfrak{L}}_{{\upsilon \nu }} }}^{{U + }} } \right)^{{{n} _{\upsilon } }} } \right] \\ & \quad \le    \left[ {\left( {1 - \mathop {\max }\limits_{\nu } \mathop {\max }\limits_{\upsilon } \left\{ {{\rm N}_{{{\mathfrak{L}}_{{\upsilon \nu }} }}^{{L + }} } \right\}} \right),~\left( {1 - \mathop {\max }\limits_{{\nu ~}} \mathop {\max }\limits_{\upsilon } \left\{ {{\rm O}_{{{\mathfrak{L}}_{{\upsilon \nu }} }}^{{U + }} } \right\}} \right)} \right] \\ & \quad +     \iota \left[ {\left( {1 - \mathop {\max }\limits_{\nu } \mathop {\max }\limits_{\upsilon } \left\{ {{\rm N}_{{{\mathfrak{L}}_{{\upsilon \nu }} }}^{{L + }} } \right\}} \right),~\left( {1 - \mathop {\max }\limits_{{\nu ~}} \mathop {\max }\limits_{\upsilon } \left\{ {{\rm O}_{{{\mathfrak{L}}_{{\upsilon \nu }} }}^{{U + }} } \right\}} \right)} \right] \\ & \quad    \Leftrightarrow \left[ {\left( {1 - \mathop {\min }\limits_{\nu } \mathop {\min }\limits_{\upsilon } \left\{ {{\rm N}_{{{\mathfrak{L}}_{{\upsilon \nu }} }}^{{L + }} } \right\}} \right)^{{\mathop \sum \nolimits_{{\nu  = 1}}^{{{\tilde{s}} }} {t} _{\nu } }} ,~\left( {1 - \mathop {\min }\limits_{\nu } \mathop {\min }\limits_{\upsilon } \left\{ {{\rm O}_{{{\mathfrak{L}}_{{\upsilon \nu }} }}^{{U + }} } \right\}} \right)^{{\mathop \sum \nolimits_{{\nu  = 1}}^{{{\tilde{s}} }} {t} _{\nu } }} } \right] \\ & \quad   +   \iota \left[ {\left( {1 - \mathop {\min }\limits_{\nu } \mathop {\min }\limits_{\upsilon } \left\{ {{\rm N}_{{{\mathfrak{L}}_{{\upsilon \nu }} }}^{{L + }} } \right\}} \right)^{{\mathop \sum \nolimits_{{\nu  = 1}}^{{{\tilde{s}} }} {t} _{\nu } }} ,\left( {1 - \mathop {\min }\limits_{\nu } \mathop {\min }\limits_{\upsilon } \left\{ {{\rm O}_{{{\mathfrak{L}}_{{\upsilon \nu }} }}^{{U + }} } \right\}} \right)^{{\mathop \sum \nolimits_{{\nu  = 1}}^{{{\tilde{s}} }} {t} _{\nu } }} } \right] \hfill \\  \end{aligned}   $$

$$  \begin{aligned}    &  \le \left[ {\mathop \prod \limits_{{\nu  = 1}}^{{{\tilde{s}} }} \left( {\mathop \prod \limits_{{\upsilon  = 1}}^{{{\tilde{r}} }} \left( {1 - {\rm N}_{{{\mathfrak{L}}_{{\upsilon \nu }} }}^{{L + }} } \right)^{{{n} _{\upsilon } }} } \right)^{{\mathop \sum \nolimits_{{\nu  = 1}}^{{{\tilde{s}} }} {t} _{\nu } }} ,~\mathop \prod \limits_{{\nu  = 1}}^{{{\tilde{s}} }} \left( {\mathop \prod \limits_{{\upsilon  = 1}}^{{{\tilde{r}} }} \left( {1 - {\rm O}_{{{\mathfrak{L}}_{{\upsilon \nu }} }}^{{U + }} } \right)^{{{n} _{\upsilon } }} } \right)^{{\mathop \sum \nolimits_{{\nu  = 1}}^{{{\tilde{s}} }} {t} _{\nu } }} } \right] \\     & \quad  + \iota \left[ {\mathop \prod \limits_{{\nu  = 1}}^{{{\tilde{s}} }} \left( {\mathop \prod \limits_{{\upsilon= 1}}^{{{\tilde{r}} }} \left( {1 - {\rm N}_{{{\mathfrak{L}}_{{\upsilon \nu }} }}^{{L + }} } \right)^{{{n} _{\upsilon } }} } \right)^{{\mathop \sum \nolimits_{{\nu  = 1}}^{{{\tilde{s}} }} {t} _{\nu } }} ,~\mathop \prod \limits_{{\nu  = 1}}^{{{\tilde{s}} }} \left( {\mathop \prod \limits_{{\upsilon  = 1}}^{{{\tilde{r}} }} \left( {1 - {\rm O}_{{{\mathfrak{L}}_{{\upsilon \nu }} }}^{{U + }} } \right)^{{{n} _{\upsilon } }} } \right)^{{\mathop \sum \nolimits_{{\nu  = 1}}^{{{\tilde{s}} }} {t} _{\nu } }} } \right] \\     &  \le \left[ {\left( {1 - \mathop {\max }\limits_{\nu } \mathop {\max 
}\limits_{\upsilon } \left\{ {{\rm N}_{{{\mathfrak{L}}_{{\upsilon \nu }} }}^{{L + }} } \right\}} \right)^{{\mathop \sum \nolimits_{{\nu  = 1}}^{{{\tilde{s}} }} {t} _{\nu } }} ,~\left( {1 - \mathop {\max }\limits_{{\nu ~}} \mathop {\max }\limits_{\upsilon } \left\{ {{\rm O}_{{{\mathfrak{L}}_{{\upsilon \nu }} }}^{{U + }} } \right\}} \right)^{{\mathop \sum \nolimits_{{\nu  = 1}}^{{{\tilde{s}} }} {t} _{\nu } }} } \right] \\     & \quad  + \iota \left[ {\left( {1 - \mathop {\max }\limits_{\nu } \mathop {\max }\limits_{\upsilon } \left\{ {{\rm N}_{{{\mathfrak{L}}_{{\upsilon \nu }} }}^{{L + }} } \right\}} \right)^{{\mathop \sum \nolimits_{{\nu  = 1}}^{{{\tilde{s}} }} {t} _{\nu } }} ,~\left( {1 - \mathop {\max }\limits_{{\nu ~}} \mathop {\max }\limits_{\upsilon } \left\{ {{\rm O}_{{{\mathfrak{L}}_{{\upsilon \nu }} }}^{{U + }} } \right\}} \right)^{{\mathop \sum \nolimits_{{\nu  = 1}}^{{{\tilde{s}} }} {t} _{\nu } }} } \right] \\     &  \Leftrightarrow \left[ {\left( {1 - \mathop {\min }\limits_{\nu } \mathop {\min }\limits_{\upsilon } \left\{ {{\rm N}_{{{\mathfrak{L}}_{{\upsilon \nu }} }}^{{L + }} } \right\}} \right),~\left( {1 - \mathop {\min }\limits_{\nu } \mathop {\min }\limits_{\upsilon } \left\{ {{\rm O}_{{{\mathfrak{L}}_{{\upsilon \nu }} }}^{{U + }} } \right\}} \right)} \right] \\     & \quad  + \iota \left[ {\left( {1 - \mathop {\min }\limits_{\nu } \mathop {\min }\limits_{\upsilon } \left\{ {{\rm N}_{{{\mathfrak{L}}_{{\upsilon \nu }} }}^{{L + }} } \right\}} \right),\left( {1 - \mathop {\min }\limits_{\nu } \mathop {\min }\limits_{\upsilon } \left\{ {{\rm O}_{{{\mathfrak{L}}_{{\upsilon \nu }} }}^{{U + }} } \right\}} \right)} \right] \\     &    \le \left[ {\mathop \prod \limits_{{\nu  = 1}}^{{{\tilde{s}} }} \left( {\mathop \prod \limits_{{\upsilon  = 1}}^{{{\tilde{r}} }} \left( {1 - {\rm N}_{{{\mathfrak{L}}_{{\upsilon \nu }} }}^{{L + }} } \right)^{{{n} _{\upsilon } }} } \right)^{{\mathop \sum \nolimits_{{\nu  = 1}}^{{{\tilde{s}} }} {t} _{\nu } }} ,~\mathop \prod \limits_{{\nu  = 1}}^{{{\tilde{s}} }} \left( {\mathop \prod \limits_{{\upsilon  = 1}}^{{{\tilde{r}} }} \left( {1 - {\rm O}_{{{\mathfrak{L}}_{{\upsilon \nu }} }}^{{U + }} } \right)^{{{n} _{\upsilon } }} } \right)^{{\mathop \sum \nolimits_{{\nu  = 1}}^{{{\tilde{s}} }} {t} _{\nu } }} } \right]  \\     & \quad  +     \iota \left[ {\mathop \prod \limits_{{\nu  = 1}}^{{{\tilde{s}} }} \left( {\mathop \prod \limits_{{\upsilon  = 1}}^{{{\tilde{r}} }} \left( {1 - {\rm N}_{{{\mathfrak{L}}_{{\upsilon \nu }} }}^{{L + }} } \right)^{{{n} _{\upsilon } }} } \right)^{{\mathop \sum \nolimits_{{\nu  = 1}}^{{{\tilde{s}} }} {t} _{\nu } }} ,~\mathop \prod \limits_{{\nu  = 1}}^{{{\tilde{s}} }} \left( {\mathop \prod \limits_{{\upsilon  = 1}}^{{{\tilde{r}} }} \left( {1 - {\rm O}_{{{\mathfrak{L}}_{{\upsilon \nu }} }}^{{U + }} } \right)^{{{n} _{\upsilon } }} } \right)^{{\mathop \sum \nolimits_{{\nu  = 1}}^{{{\tilde{s}} }} {t} _{\nu } }} } \right] \\     & \le     \left[ {\left( {1 - \mathop {\max }\limits_{\nu } \mathop {\max }\limits_{\upsilon } \left\{ {{\rm N}_{{{\mathfrak{L}}_{{\upsilon \nu }} }}^{{L + }} } \right\}} \right),~\left( {1 - \mathop {\max }\limits_{{\nu ~}} \mathop {\max }\limits_{\upsilon } \left\{ {{\rm O}_{{{\mathfrak{L}}_{{\upsilon \nu }} }}^{{U + }} } \right\}} \right)} \right] \\     & \quad +      \iota \left[ {\left( {1 - \mathop {\max }\limits_{\nu } \mathop {\max }\limits_{\upsilon } \left\{ {{\rm N}_{{{\mathfrak{L}}_{{\upsilon \nu }} }}^{{L + }} } \right\}} \right),~\left( {1 - \mathop {\max }\limits_{{\nu ~}} \mathop {\max }\limits_{\upsilon } \left\{ {{\rm O}_{{{\mathfrak{L}}_{{\upsilon \nu }} }}^{{U + }} } \right\}} \right)} \right] \\  \end{aligned}   $$

Therefore,

12 $$ \begin{aligned} & \left[ {\left( {\mathop {\min }\limits_{\nu } \mathop {\min }\limits_{\upsilon } \left\{ {{\rm N}_{{{\mathfrak{L}}_{\upsilon \nu } }}^{L + } } \right\}} \right), \left( {\mathop {\min }\limits_{\nu } \mathop {\min }\limits_{\upsilon } \left\{ {{\rm O}_{{{\mathfrak{L}}_{\upsilon \nu } }}^{U + } } \right\}} \right)} \right] + \iota \left[ {\left( {\mathop {\min }\limits_{\nu } \mathop {\min }\limits_{\upsilon } \left\{ {{\rm N}_{{{\mathfrak{L}}_{\upsilon \nu } }}^{L + } } \right\}} \right),\left( {\mathop {\min }\limits_{\nu } \mathop {\min }\limits_{\upsilon } \left\{ {{\rm O}_{{{\mathfrak{L}}_{\upsilon \nu } }}^{U + } } \right\}} \right)} \right] \hfill \\ & \quad  \le \left[ {1 - \mathop \prod \limits_{\nu = 1}^{S} \left( {\mathop \prod \limits_{\upsilon = 1}^{{{\tilde{r}} }} \left( {1 - {\rm N}_{{{\mathfrak{L}}_{\upsilon \nu } }}^{L + } } \right)^{{{n}_{\upsilon } }} } \right)^{{{\mathop \sum \limits_{\nu = 1}{{\tilde{s}} }} {t}_{\nu } }} , 1 - \mathop \prod \limits_{\nu = 1}^{{{\tilde{s}} }} \left( {\mathop \prod \limits_{\upsilon = 1}^{{{\tilde{r}} }} \left( {1 - {\rm O}_{{{\mathfrak{L}}_{\upsilon \nu } }}^{U + } } \right)^{{{n}_{\upsilon } }} } \right)^{{{\mathop \sum \limits_{\nu = 1}{{\tilde{s}} }} {t}_{\nu } }} } \right] \\ & \qquad  +  \iota \left[ {1 - \mathop \prod \limits_{\nu = 1}^{{{\tilde{s}} }} \left( {\mathop \prod \limits_{\upsilon = 1}^{{{\tilde{r}} }} \left( {1 - {\rm N}_{{{\mathfrak{L}}_{\upsilon \nu } }}^{L + } } \right)^{{{n}_{\upsilon } }} } \right)^{{{\mathop \sum \limits_{\nu = 1}{{\tilde{s}} }} {t}_{\nu } }} , 1 - \mathop \prod \limits_{\nu = 1}^{{{\tilde{s}} }} \left( {\mathop \prod \limits_{\upsilon = 1}^{{{\tilde{r}} }} \left( {1 - {\rm O}_{{{\mathfrak{L}}_{\upsilon \nu } }}^{U + } } \right)^{{{n}_{\upsilon } }} } \right)^{{{\mathop \sum \limits_{\nu = 1}{{\tilde{s}} }} {t}_{\nu } }} } \right] \\ & \quad \le  \left[ {\left( {\mathop {\max }\limits_{\nu } \mathop {\max }\limits_{\upsilon } \left\{ {{\rm N}_{{{\mathfrak{L}}_{\upsilon \nu } }}^{L + } } \right\}} \right), \left( {\mathop {\max }\limits_{\nu } \mathop {\max }\limits_{\upsilon } \left\{ {{\rm O}_{{{\mathfrak{L}}_{\upsilon \nu } }}^{U + } } \right\}} \right)} \right] + \iota \left[ {\left( {\mathop {\max }\limits_{\nu } \mathop {\max }\limits_{\upsilon } \left\{ {{\rm N}_{{{\mathfrak{L}}_{\upsilon \nu } }}^{L + } } \right\}} \right), \left( {\mathop {\max }\limits_{\nu } \mathop {\max }\limits_{\upsilon } \left\{ {{\rm O}_{{{\mathfrak{L}}_{\upsilon \nu } }}^{U + } } \right\}} \right)} \right] \hfill \\ \end{aligned} $$

Again,

13 $$ \begin{gathered} \left[ {\mathop {\max }\limits_{\nu } \mathop {\max }\limits_{\upsilon } \left\{ {{\rm N}_{{{\mathfrak{L}}_{\upsilon \nu } }}^{L - } } \right\}, \mathop {\max }\limits_{\nu } \mathop {\max }\limits_{\upsilon } \left\{ {{\rm O}_{{{\mathfrak{L}}_{\upsilon \nu } }}^{U - } } \right\}} \right] + \iota \left[ {\mathop {\max }\limits_{\nu } \mathop {\max }\limits_{\upsilon } \left\{ {{\rm N}_{{{\mathfrak{L}}_{\upsilon \nu } }}^{L - } } \right\}, \mathop {\max }\limits_{\nu } \mathop {\max }\limits_{\upsilon } \left\{ {{\rm O}_{{{\mathfrak{L}}_{\upsilon \nu } }}^{U - } } \right\}} \right] \le \left[ {{\rm N}_{{{\mathfrak{L}}_{\upsilon \nu } }}^{L - } , {\rm O}_{{{\mathfrak{L}}_{\upsilon \nu } }}^{U - } } \right] + \iota \left[ {{\rm N}_{{{\mathfrak{L}}_{\upsilon \nu } }}^{L - } , {\rm O}_{{{\mathfrak{L}}_{\upsilon \nu } }}^{U - } } \right] \le \hfill \\ \left[ {\mathop {\min }\limits_{\nu } \mathop {\min }\limits_{\upsilon } \left\{ {{\rm N}_{{{\mathfrak{L}}_{\upsilon \nu } }}^{L - } } \right\}, \mathop {\min }\limits_{\nu } \mathop {\min }\limits_{\upsilon } \left\{ {{\rm O}_{{{\mathfrak{L}}_{\upsilon \nu } }}^{U - } } \right\}} \right] + \iota \left[ {\mathop {\min }\limits_{\nu } \mathop {\min }\limits_{\upsilon } \left\{ {{\rm N}_{{{\mathfrak{L}}_{\upsilon \nu } }}^{L - } } \right\}, \mathop {\min }\limits_{\nu } \mathop {\min }\limits_{\upsilon } \left\{ {{\rm O}_{{{\mathfrak{L}}_{\upsilon \nu } }}^{U - } } \right\}} \right] \hfill \\ \end{gathered} $$

$$  \begin{aligned}    &  \Leftrightarrow \left[ {\left( {\mathop {\max }\limits_{\nu } \mathop {\max }\limits_{\upsilon } \left\{ {{\rm N}_{{{\mathfrak{L}}_{{\upsilon \nu }} }}^{{L - }} } \right\}} \right)^{{\mathop \sum \nolimits_{{\upsilon  = 1}}^{{{\tilde{r}} }} {n} _{\upsilon } }} ,~\left( {\mathop {\max }\limits_{\nu } \mathop {\max }\limits_{\upsilon } \left\{ {{\rm O}_{{{\mathfrak{L}}_{{\upsilon \nu }} }}^{{U - }} } \right\}} \right)^{{\mathop \sum \nolimits_{{\upsilon  = 1}}^{{{\tilde{r}} }} {n} _{\upsilon } }} } \right] + \iota \left[ {\left( {\mathop {\max }\limits_{\nu } \mathop {\max }\limits_{\upsilon } \left\{ {{\rm N}_{{{\mathfrak{L}}_{{\upsilon \nu }} }}^{{L - }} } \right\}} \right)^{{\mathop \sum \nolimits_{{\upsilon  = 1}}^{{{\tilde{r}} }} {n} _{\upsilon } }} ,~\left( {\mathop {\max }\limits_{\nu } \mathop {\max }\limits_{\upsilon } \left\{ {{\rm O}_{{{\mathfrak{L}}_{{\upsilon \nu }} }}^{{U - }} } \right\}} \right)^{{\mathop \sum \nolimits_{{\upsilon  = 1}}^{{{\tilde{r}} }} {mn} _{\upsilon } }} } \right] \\     &  \le \left[ {\mathop \prod \limits_{{\upsilon  = 1}}^{{{\tilde{r}} }} \left( {{\rm N}_{{{\mathfrak{L}}_{{\upsilon \nu }} }}^{{L - }} } \right)^{{{n} _{\upsilon } }} ,~\mathop \prod \limits_{{\upsilon  = 1}}^{{{\tilde{r}} }} \left( {{\rm O}_{{{\mathfrak{L}}_{{\upsilon \nu }} }}^{{U - }} } \right)^{{{n} _{\upsilon } }} } \right] + \iota \left[ {\mathop \prod \limits_{{\upsilon  = 1}}^{{{\tilde{r}} }} \left( {{\rm N}_{{{\mathfrak{L}}_{{\upsilon \nu }} }}^{{L - }} } \right)^{{{n} _{\upsilon } }} ,~\mathop \prod \limits_{{\upsilon  = 1}}^{{{\tilde{r}} }} \left( {{\rm O}_{{{\mathfrak{L}}_{{\upsilon \nu }} }}^{{U - }} } \right)^{{{n} _{\upsilon } }} } \right] \\     &  \le \left[ {\left( {\mathop {\min }\limits_{\nu } \mathop {\min }\limits_{\upsilon } \left\{ {{\rm N}_{{{\mathfrak{L}}_{{\upsilon \nu }} }}^{{L - }} } \right\}} \right)^{{\mathop \sum \nolimits_{{\upsilon  = 1}}^{{{\tilde{r}} }} {n} _{\upsilon } }} ,~\left( {\mathop {\min }\limits_{\nu } \mathop {\min }\limits_{\upsilon } \left\{ {{\rm O}_{{{\mathfrak{L}}_{{\upsilon \nu }} }}^{{U - }} } \right\}} \right)^{{\mathop \sum \nolimits_{{\upsilon  = 1}}^{{{\tilde{r}} }} {n} _{\upsilon } }} } \right] + \iota \left[ {\left( {\mathop {\min }\limits_{\nu } \mathop {\min }\limits_{\upsilon } \left\{ {{\rm N}_{{{\mathfrak{L}}_{{\upsilon \nu }} }}^{{L - }} } \right\}} \right)^{{\mathop \sum \nolimits_{{\upsilon  = 1}}^{{{\tilde{r}} }} {n} _{\upsilon } }} ,~\left( {\mathop {\min }\limits_{\nu } \mathop {\min }\limits_{\upsilon } \left\{ {{\rm O}_{{{\mathfrak{L}}_{{\upsilon \nu }} }}^{{U - }} } \right\}} \right)^{{\mathop \sum \nolimits_{{\upsilon  = 1}}^{{{\tilde{r}} }} {n} _{\upsilon } }} } \right] \\     &  \Leftrightarrow \left[ {\left( {\mathop {\max }\limits_{\nu } \mathop {\max }\limits_{\upsilon } \left\{ {{\rm N}_{{{\mathfrak{L}}_{{\upsilon \nu }} }}^{{L - }} } \right\}} \right),~\left( {\mathop {\max }\limits_{\nu } \mathop {\max }\limits_{\upsilon } \left\{ {{\rm O}_{{{\mathfrak{L}}_{{\upsilon \nu }} }}^{{U - }} } \right\}} \right)} \right] + \iota \left[ {\left( {\mathop {\max }\limits_{\nu } \mathop {\max }\limits_{\upsilon } \left\{ {{\rm N}_{{{\mathfrak{L}}_{{\upsilon \nu }} }}^{{L - }} } \right\}} \right),~\left( {\mathop {\max }\limits_{\nu } \mathop {\max }\limits_{\upsilon } \left\{ {{\rm O}_{{{\mathfrak{L}}_{{\upsilon \nu }} }}^{{U - }} } \right\}} \right)} \right] \\     &  \le \left[ {\mathop \prod \limits_{{\upsilon  = 1}}^{{{\tilde{r}} }} \left( {{\rm N}_{{{\mathfrak{L}}_{{\upsilon \nu }} }}^{{L - }} } \right)^{{{n} _{\upsilon } }} ,~\mathop \prod \limits_{{\upsilon  = 1}}^{{{\tilde{r}} }} \left( {{\rm O}_{{{\mathfrak{L}}_{{\upsilon \nu }} }}^{{U - }} } \right)^{{{n} _{\upsilon } }} } \right] + \iota \left[ {\mathop \prod \limits_{{\upsilon  = 1}}^{{{\tilde{r}} }} \left( {{\rm N}_{{{\mathfrak{L}}_{{\upsilon \nu }} }}^{{L - }} } \right)^{{{n} _{\upsilon } }} ,~\mathop \prod \limits_{{\upsilon  = 1}}^{{{\tilde{r}} }} \left( {{\rm O}_{{{\mathfrak{L}}_{{\upsilon \nu }} }}^{{U - }} } \right)^{{{n} _{\upsilon } }} } \right] \\     &  \le \left[ {\left( {\mathop {\min }\limits_{\nu } \mathop {\min }\limits_{\upsilon } \left\{ {{\rm N}_{{{\mathfrak{L}}_{{\upsilon \nu }} }}^{{L - }} } \right\}} \right),~\left( {\mathop {\min }\limits_{\nu } \mathop {\min }\limits_{\upsilon } \left\{ {{\rm O}_{{{\mathfrak{L}}_{{\upsilon \nu }} }}^{{U - }} } \right\}} \right)} \right] + \iota \left[ {\left( {\mathop {\min }\limits_{\nu } \mathop {\min }\limits_{\upsilon } \left\{ {{\rm N}_{{{\mathfrak{L}}_{{\upsilon \nu }} }}^{{L - }} } \right\}} \right),~\left( {\mathop {\min }\limits_{\nu } \mathop {\min }\limits_{\upsilon } \left\{ {{\rm O}_{{{\mathfrak{L}}_{{\upsilon \nu }} }}^{{U - }} } \right\}} \right)} \right] \\     &  \Leftrightarrow \left[ {\left( {\mathop {\max }\limits_{\nu } \mathop {\max }\limits_{\upsilon } \left\{ {{\rm N}_{{{\mathfrak{L}}_{{\upsilon \nu }} }}^{{L - }} } \right\}} \right)^{{{t} _{\nu } }} ,~\left( {\mathop {\max }\limits_{\nu } \mathop {\max }\limits_{\upsilon } \left\{ {{\rm O}_{{{\mathfrak{L}}_{{\upsilon \nu }} }}^{{U - }} } \right\}} \right)^{{{t}_{\nu } }} } \right] + \iota \left[ {\left( {\mathop {\max }\limits_{\nu } \mathop {\max }\limits_{\upsilon } \left\{ {{\rm N}_{{{\mathfrak{L}}_{{\upsilon \nu }} }}^{{L - }} } \right\}} \right)^{{{t} _{\nu } }} ,~\left( {\mathop {\max }\limits_{\nu } \mathop {\max }\limits_{\upsilon } \left\{ {{\rm O}_{{{\mathfrak{L}}_{{\upsilon \nu }} }}^{{U - }} } \right\}} \right)^{{{t} _{\nu } }} } \right] \\     &  \le \left[ {\left( {\mathop \prod \limits_{{\upsilon  = 1}}^{{{\tilde{r}} }} \left( {{\rm N}_{{{\mathfrak{L}}_{{\upsilon \nu }} }}^{{L - }} } \right)^{{{n} _{\upsilon } }} } \right)^{{{t} _{\nu } }} ,~\left( {\mathop \prod \limits_{{\upsilon  = 1}}^{{{\tilde{r}} }} \left( {{\rm O}_{{{\mathfrak{L}}_{{\upsilon \nu }} }}^{{U - }} } \right)^{{{n} _{\upsilon } }} } \right)^{{{t} _{\nu } }} } \right] + \iota \left[ {\left( {\mathop \prod \limits_{{\upsilon  = 1}}^{{{\tilde{r}} }} \left( {{\rm N}_{{{\mathfrak{L}}_{{\upsilon \nu }} }}^{{L - }} } \right)^{{{n} _{\upsilon } }} } \right)^{{{t} _{\nu } }} ,~\left( {\mathop \prod \limits_{{\upsilon  = 1}}^{{{\tilde{r}} }} \left( {{\rm O}_{{{\mathfrak{L}}_{{\upsilon \nu }} }}^{{U - }} } \right)^{{{n} _{\upsilon } }} } \right)^{{{t} _{\nu } }} } \right] \\     & \quad \left[ {\left( {\mathop {\min }\limits_{\nu } \mathop {\min }\limits_{\upsilon } \left\{ {{\rm N}_{{{\mathfrak{L}}_{{\upsilon \nu }} }}^{{L - }} } \right\}} \right)^{{{t} _{\nu } }} ,~\left( {\mathop {\min }\limits_{\nu } \mathop {\min }\limits_{\upsilon } \left\{ {{\rm O}_{{{\mathfrak{L}}_{{\upsilon \nu }} }}^{{U - }} } \right\}} \right)^{{{t} _{\nu } }} } \right] + \iota \left[ {\left( {\mathop {\min }\limits_{\nu } \mathop {\min }\limits_{\upsilon } \left\{ {{\rm N}_{{{\mathfrak{L}}_{{\upsilon \nu }} }}^{{L - }} } \right\}} \right)^{{{t} _{\nu } }} ,~\left( {\mathop {\min }\limits_{\nu } \mathop {\min }\limits_{\upsilon } \left\{ {{\rm O}_{{{\mathfrak{L}}_{{\upsilon \nu }} }}^{{U - }} } \right\}} \right)^{{{t} _{\nu } }} } \right] \\     &  \Leftrightarrow \left[ {\left( {\mathop {\max }\limits_{\nu } \mathop {\max }\limits_{\upsilon } \left\{ {{\rm N}_{{{\mathfrak{L}}_{{\upsilon \nu }} }}^{{L - }} } \right\}} \right)^{{\mathop \sum \nolimits_{{\nu  = 1}}^{{{\tilde{s}} }} {t} _{\nu } }} ,~\left( {\mathop {\max }\limits_{\nu } \mathop {\max }\limits_{\upsilon } \left\{ {{\rm O}_{{{\mathfrak{L}}_{{\upsilon \nu }} }}^{{U - }} } \right\}} \right)^{{\mathop \sum \nolimits_{{\nu  = 1}}^{{{\tilde{s}} }} {t} _{\nu } }} } \right] + \iota \left[ {\left( {\mathop {\max }\limits_{\nu } \mathop {\max }\limits_{\upsilon } \left\{ {{\rm N}_{{{\mathfrak{L}}_{{\upsilon \nu }} }}^{{L - }} } \right\}} \right)^{{\mathop \sum \nolimits_{{\nu  = 
1}}^{{{\tilde{s}} }} {t} _{\nu } }} ,~\left( {\mathop {\max }\limits_{\nu } \mathop {\max }\limits_{\upsilon } \left\{ {{\rm O}_{{{\mathfrak{L}}_{{\upsilon \nu }} }}^{{U - }} } \right\}} \right)^{{\mathop \sum \nolimits_{{\nu  = 1}}^{{{\tilde{s}} }} {t} _{\nu } }} } \right] \\     &  \le \left[ {\mathop \prod \limits_{{\nu  = 1}}^{{{\tilde{s}} }} \left( {\mathop \prod \limits_{{\upsilon  = 1}}^{{{\tilde{r}} }} \left( {{\rm N}_{{{\mathfrak{L}}_{{\upsilon \nu }} }}^{{L - }} } \right)^{{{n} _{\upsilon } }} } \right)^{{{t} _{\nu } }} ,~\mathop \prod \limits_{{\nu  = 1}}^{{{\tilde{s}} }} \left( {\mathop \prod \limits_{{\upsilon  = 1}}^{{{\tilde{r}} }} \left( {{\rm O}_{{{\mathfrak{L}}_{{\upsilon \nu }} }}^{{U - }} } \right)^{{{n} _{\upsilon } }} } \right)^{{{t} _{\nu } }} } \right] \\     & \quad  + \iota \left[ {\mathop \prod \limits_{{\nu  = 1}}^{{{\tilde{s}} }} \left( {\mathop \prod \limits_{{\upsilon  = 1}}^{{{\tilde{r}} }} \left( {{\rm N}_{{{\mathfrak{L}}_{{\upsilon \nu }} }}^{{L - }} } \right)^{{{n} _{\upsilon } }} } \right)^{{{t} _{\nu } }} ,~\mathop \prod \limits_{{\nu  = 1}}^{{{\tilde{s}} }} \left( {\mathop \prod \limits_{{\upsilon  = 1}}^{{{\tilde{r}} }} \left( {{\rm O}_{{{\mathfrak{L}}_{{\upsilon \nu }} }}^{{U - }} } \right)^{{{n} _{\upsilon } }} } \right)^{{{t} _{\nu } }} } \right] \\     &  \le \left[ {\left( {\mathop {\min }\limits_{\nu } \mathop {\min }\limits_{\upsilon } \left\{ {{\rm N}_{{{\mathfrak{L}}_{{\upsilon \nu }} }}^{{L - }} } \right\}} \right)^{{\mathop \sum \nolimits_{{\nu  = 1}}^{{{\tilde{s}} }} {t} _{\nu } }} ,~\left( {\mathop {\min }\limits_{\nu } \mathop {\min }\limits_{\upsilon } \left\{ {{\rm O}_{{{\mathfrak{L}}_{{\upsilon \nu }} }}^{{U - }} } \right\}} \right)^{{\mathop \sum \nolimits_{{\nu  = 1}}^{{{\tilde{s}} }} {t} _{\nu } }} } \right] + \iota \left[ {\left( {\mathop {\min }\limits_{\nu } \mathop {\min }\limits_{\upsilon } \left\{ {{\rm N}_{{{\mathfrak{L}}_{{\upsilon \nu }} }}^{{L - }} } \right\}} \right)^{{\mathop \sum \nolimits_{{\nu  = 1}}^{{{\tilde{s}} }} {t} _{\nu } }} ,~\left( {\mathop {\min }\limits_{\nu } \mathop {\min }\limits_{\upsilon } \left\{ {{\rm O}_{{{\mathfrak{L}}_{{\upsilon \nu }} }}^{{U - }} } \right\}} \right)^{{\mathop \sum \nolimits_{{\nu  = 1}}^{{{\tilde{s}} }} {t} _{\nu } }} } \right] \\  \end{aligned}   $$

Hence we obtain,

14 $$ \begin{aligned} &  \left[ {\left( {\mathop {\max }\limits_{\nu } \mathop {\max }\limits_{\upsilon } \left\{ {{\rm N}_{{{\mathfrak{L}}_{\upsilon \nu } }}^{L - } } \right\}} \right), \left( {\mathop {\max }\limits_{\nu } \mathop {\max }\limits_{\upsilon } \left\{ {{\rm O}_{{{\mathfrak{L}}_{\upsilon \nu } }}^{U - } } \right\}} \right)} \right] + \iota \left[ {\left( {\mathop {\max }\limits_{\nu } \mathop {\max }\limits_{\upsilon } \left\{ {{\rm N}_{{{\mathfrak{L}}_{\upsilon \nu } }}^{L - } } \right\}} \right), \left( {\mathop {\max }\limits_{\nu } \mathop {\max }\limits_{\upsilon } \left\{ {{\rm O}_{{{\mathfrak{L}}_{\upsilon \nu } }}^{U - } } \right\}} \right)} \right] \hfill \\ & \quad  \le \left[ {\mathop \prod \limits_{\nu = 1}^{{{\tilde{s}} }} \left( {\mathop \prod \limits_{\upsilon = 1}^{{{\tilde{r}} }} \left( {{\rm N}_{{{\mathfrak{L}}_{\upsilon \nu } }}^{L - } } \right)^{{n_{\upsilon } }} } \right)^{{t_{\nu } }} , \mathop \prod \limits_{\nu = 1}^{{{\tilde{s}} }} \left( {\mathop \prod \limits_{\upsilon = 1}^{{{\tilde{r}} }} \left( {{\rm O}_{{{\mathfrak{L}}_{\upsilon \nu } }}^{U - } } \right)^{{n_{\upsilon } }} } \right)^{{t_{\nu } }} } \right] \\ & \qquad +  \iota \left[ {\mathop \prod \limits_{\nu = 1}^{{{\tilde{s}} }} \left( {\mathop \prod \limits_{\upsilon = 1}^{{{\tilde{r}} }} \left( {{\rm N}_{{{\mathfrak{L}}_{\upsilon \nu } }}^{L - } } \right)^{{n_{\upsilon } }} } \right)^{{t_{\nu } }} , \mathop \prod \limits_{\nu = 1}^{{{\tilde{s}} }} \left( {\mathop \prod \limits_{\upsilon = 1}^{{{\tilde{r}} }} \left( {{\rm O}_{{{\mathfrak{L}}_{\upsilon \nu } }}^{U - } } \right)^{{n_{\upsilon } }} } \right)^{{t_{\nu } }} } \right]\\ & \quad \le \left[ {\left( {\mathop {\min }\limits_{\nu } \mathop {\min }\limits_{\upsilon } \left\{ {{\rm N}_{{{\mathfrak{L}}_{\upsilon \nu } }}^{L - } } \right\}} \right), \left( {\mathop {\min }\limits_{\nu } \mathop {\min }\limits_{\upsilon } \left\{ {{\rm O}_{{{\mathfrak{L}}_{\upsilon \nu } }}^{U - } } \right\}} \right)} \right] + \iota \left[ {\left( {\mathop {\min }\limits_{\nu } \mathop {\min }\limits_{\upsilon } \left\{ {{\rm N}_{{{\mathfrak{L}}_{\upsilon \nu } }}^{L - } } \right\}} \right), \left( {\mathop {\min }\limits_{\nu } \mathop {\min }\limits_{\upsilon } \left\{ {{\rm O}_{{{\mathfrak{L}}_{\upsilon \nu } }}^{U - } } \right\}} \right)} \right] \hfill \\ \end{aligned} $$

Suppose

$$\pounds = IVBCFSWAA\left( {{\mathfrak{L}}_{11} , {\mathfrak{L}}_{12} , \ldots , {\mathfrak{L}}_{{{\tilde{r}} {\tilde{s}} }} } \right) = \left( {{\rm E}_{{{\mathfrak{L}}_{\upsilon \nu } }}^{ + } , {\rm E}_{{{\mathfrak{L}}_{\upsilon \nu } }}^{ - } } \right) = \left( {\begin{array}{*{20}c} {\left[ {{\rm N}_{{{\mathfrak{L}}_{\upsilon \nu } }}^{L + } , {\rm O}_{{{\mathfrak{L}}_{\upsilon \nu } }}^{U + } } \right] + \iota \left[ {{\rm N}_{{{\mathfrak{L}}_{\upsilon \nu } }}^{L + } , {\rm O}_{{{\mathfrak{L}}_{\upsilon \nu } }}^{U + } } \right], } \\ {\left[ {{\rm N}_{{{\mathfrak{L}}_{\upsilon \nu } }}^{L - } , {\rm O}_{{{\mathfrak{L}}_{\upsilon \nu } }}^{U - } } \right] + \iota \left[ {{\rm N}_{{{\mathfrak{L}}_{\upsilon \nu } }}^{L - } , {\rm O}_{{{\mathfrak{L}}_{\upsilon \nu } }}^{U - } } \right]} \\ \end{array} } \right),$$

then from Eqs. (11) and (13),

$$ \begin{aligned} & \left[ {\mathop {\min }\limits_{\nu } \mathop {\min }\limits_{\upsilon } \left\{ {{\rm N}_{{{\mathfrak{L}}_{\upsilon \nu } }}^{L + } } \right\}, \mathop {\min }\limits_{\nu } \mathop {\min }\limits_{\upsilon } \left\{ {{\rm O}_{{{\mathfrak{L}}_{\upsilon \nu } }}^{U + } } \right\}} \right] + \iota \left[ {\mathop {\min }\limits_{\nu } \mathop {\min }\limits_{\upsilon } \left\{ {{\rm N}_{{{\mathfrak{L}}_{\upsilon \nu } }}^{L + } } \right\}, \mathop {\min }\limits_{\nu } \mathop {\min }\limits_{\upsilon } \left\{ {{\rm O}_{{{\mathfrak{L}}_{\upsilon \nu } }}^{U + } } \right\}} \right] \hfill \\& \quad \le \left[ {{\rm N}_{{{\mathfrak{L}}_{\upsilon \nu } }}^{L + } , {\rm O}_{{{\mathfrak{L}}_{\upsilon \nu } }}^{U + } } \right] + \iota \left[ {{\rm N}_{{{\mathfrak{L}}_{\upsilon \nu } }}^{L + } , {\rm O}_{{{\mathfrak{L}}_{\upsilon \nu } }}^{U + } } \right] \\& \quad \le  \left[ {\mathop {\max }\limits_{\nu } \mathop {\max }\limits_{\upsilon } \left\{ {{\rm N}_{{{\mathfrak{L}}_{\upsilon \nu } }}^{L + } } \right\}, \mathop {\max }\limits_{\nu } \mathop {\max }\limits_{\upsilon } \left\{ {{\rm O}_{{{\mathfrak{L}}_{\upsilon \nu } }}^{U + } } \right\}} \right] + \iota \left[ {\mathop {\max }\limits_{\nu } \mathop {\max }\limits_{\upsilon } \left\{ {{\rm N}_{{{\mathfrak{L}}_{\upsilon \nu } }}^{L + } } \right\}, \mathop {\max }\limits_{\nu } \mathop {\max }\limits_{\upsilon } \left\{ {{\rm O}_{{{\mathfrak{L}}_{\upsilon \nu } }}^{U + } } \right\}} \right] \hfill \\ \end{aligned} $$

And

$$ \begin{gathered} \left[ {\mathop {\max }\limits_{\nu } \mathop {\max }\limits_{\upsilon } \left\{ {{\rm N}_{{{\mathfrak{L}}_{\upsilon \nu } }}^{L - } } \right\}, \mathop {\max }\limits_{\nu } \mathop {\max }\limits_{\upsilon } \left\{ {{\rm O}_{{{\mathfrak{L}}_{\upsilon \nu } }}^{U - } } \right\}} \right] + \iota \left[ {\mathop {\max }\limits_{\nu } \mathop {\max }\limits_{\upsilon } \left\{ {{\rm N}_{{{\mathfrak{L}}_{\upsilon \nu } }}^{L - } } \right\}, \mathop {\max }\limits_{\nu } \mathop {\max }\limits_{\upsilon } \left\{ {{\rm O}_{{{\mathfrak{L}}_{\upsilon \nu } }}^{U - } } \right\}} \right] \hfill \\ \le \left[ {{\rm N}_{{{\mathfrak{L}}_{\upsilon \nu } }}^{L - } , {\rm O}_{{{\mathfrak{L}}_{\upsilon \nu } }}^{U - } } \right] + \iota \left[ {{\rm N}_{{{\mathfrak{L}}_{\upsilon \nu } }}^{L - } , {\rm O}_{{{\mathfrak{L}}_{\upsilon \nu } }}^{U - } } \right] \le \hfill \\ \left[ {\mathop {\min }\limits_{\nu } \mathop {\min }\limits_{\upsilon } \left\{ {{\rm N}_{{{\mathfrak{L}}_{\upsilon \nu } }}^{L - } } \right\}, \mathop {\min }\limits_{\nu } \mathop {\min }\limits_{\upsilon } \left\{ {{\rm O}_{{{\mathfrak{L}}_{\upsilon \nu } }}^{U - } } \right\}} \right] + \iota \left[ {\mathop {\min }\limits_{\nu } \mathop {\min }\limits_{\upsilon } \left\{ {{\rm N}_{{{\mathfrak{L}}_{\upsilon \nu } }}^{L - } } \right\}, \mathop {\min }\limits_{\nu } \mathop {\min }\limits_{\upsilon } \left\{ {{\rm O}_{{{\mathfrak{L}}_{\upsilon \nu } }}^{U - } } \right\}} \right] \hfill \\ \end{gathered} $$

Then by definition score function

$$ \begin{gathered} {\text{\yen}} \left( \pounds \right) = {\rm E}_{\pounds}^{ + } + {\rm E}_{\pounds}^{ - } \le \mathop {\max }\limits_{\nu } \mathop {\max }\limits_{\upsilon } \left\{ {{\rm E}_{{{\mathfrak{L}}_{\upsilon \nu } }}^{ + } } \right\} + \mathop {\min }\limits_{\nu } \mathop {\min }\limits_{\upsilon } \left\{ {{\rm E}_{{{\mathfrak{L}}_{\upsilon \nu } }}^{ - } } \right\} = {\text{\yen}} \left( {{\mathfrak{L}}_{\upsilon \nu } } \right) \hfill \\ {\text{\yen}} \left( \pounds \right) = {\rm E}_{\pounds}^{ + } + {\rm E}_{\pounds}^{ - } \ge \mathop {\min }\limits_{\nu } \mathop {\min }\limits_{\upsilon } \left\{ {{\rm E}_{{{\mathfrak{L}}_{\upsilon \nu } }}^{ + } } \right\} + \mathop {\max }\limits_{\nu } \mathop {\max }\limits_{\upsilon } \left\{ {{\rm E}_{{{\mathfrak{L}}_{\upsilon \nu } }}^{ - } } \right\} = {\text{\yen}} \left( {{\mathfrak{L}}_{\upsilon \nu } } \right) \hfill \\ \end{gathered} $$

Here, we have three cases be demonstrated:

*Case* 1 If ${\text{\yen}} \left( {{\mathfrak{L}}_{\upsilon \nu } } \right) < {\text{\yen}} \left( {{\mathfrak{L}}_{\upsilon \nu }^{ + } } \right)$ and ${\text{\yen}} \left( {{\mathfrak{L}}_{\upsilon \nu } } \right) > {\text{\yen}} \left( {{\mathfrak{L}}_{\upsilon \nu }^{ - } } \right)$, by comparison of two IVBCFSNs, we have

$$ {\text{\yen}} \left( {{\mathfrak{L}}_{\upsilon \nu }^{ - } } \right) \le IVBCFSWAA\left( {{\mathfrak{L}}_{11} , {\mathfrak{L}}_{12} , \ldots , {\mathfrak{L}}_{{{\tilde{r}} {\tilde{s}} }} } \right) \le {\text{\yen}} \left( {{\mathfrak{L}}_{\upsilon \nu }^{ + } } \right) $$

*Case* 2 If ${\text{\yen}} \left( {{\mathfrak{L}}_{\upsilon \nu } } \right) = {\text{\yen}} \left( {{\mathfrak{L}}_{\upsilon \nu }^{ + } } \right)$, i.e., ${\rm E}_{\pounds}^{ + } + {\rm E}_{\pounds}^{ - } = \mathop {\max }\limits_{\nu } \mathop {\max }\limits_{\upsilon } \left\{ {{\rm E}_{{{\mathfrak{L}}_{\upsilon \nu } }}^{ + } } \right\} + \mathop {\min }\limits_{\nu } \mathop {\min }\limits_{\upsilon } \left\{ {{\rm E}_{{{\mathfrak{L}}_{\upsilon \nu } }}^{ - } } \right\}$ then by above inequalities

$$ {\rm E}_{\pounds}^{ + } = \mathop {\max }\limits_{\nu } \mathop {\max }\limits_{\upsilon } \left\{ {{\rm E}_{{{\mathfrak{L}}_{\upsilon \nu } }}^{ + } } \right\}\;{\text{and}}\;{\rm E}_{\pounds}^{ - } = \mathop {\min }\limits_{\nu } \mathop {\min }\limits_{\upsilon } \left\{ {{\rm E}_{{{\mathfrak{L}}_{\upsilon \nu } }}^{ - } } \right\} $$

Thus,

$$\theta = {\rm E}_{{\text{\pounds}}}^{ + } - {\rm E}_{{\text{\pounds}}}^{ - } = \mathop {\max }\limits_{\nu } \mathop {\max }\limits_{\upsilon } \left\{ {{\rm E}_{{{\mathfrak{L}}_{\upsilon \nu } }}^{ + } } \right\} - \mathop {\min }\limits_{\nu } \mathop {\min }\limits_{\upsilon } \left\{ {{\rm E}_{{{\mathfrak{L}}_{\upsilon \nu } }}^{ - } } \right\} = \theta \left( {{\mathfrak{L}}_{\upsilon \nu }^{ + } } \right)$$,

Then by comparison of two IVBCFSNs, we have

$$IVBCFSWAA\left( {{\mathfrak{L}}_{{11}} ,~{\mathfrak{L}}_{{12}} ,~ \ldots ,~{\mathfrak{L}}_{{{\tilde{r}} {\tilde{s}} }} } \right) = {\mathfrak{L}}_{{\upsilon \nu }}^{ + }$$

*Case* 3 If ${\text{\yen}} \left( { \mathfrak{L}_{\upsilon \nu } } \right) = {\text{\yen}} \left( { \mathfrak{L}_{\upsilon \nu }^{ - } } \right)$, i.e., ${\rm E}_{\pounds}^{ + } + {\rm E}_{\pounds}^{ - } = \mathop {\min }\limits_{\nu } \mathop {\min }\limits_{\upsilon } \left\{ {{\rm E}_{{ \mathfrak{L}_{\upsilon \nu } }}^{ + } } \right\} + \mathop {\max }\limits_{\nu } \mathop {\max }\limits_{\upsilon } \left\{ {{\rm E}_{{ \mathfrak{L}_{\upsilon \nu } }}^{ - } } \right\}$, then by above inequalities.

$$ {\rm E}_{\pounds}^{ + } = \mathop {\min }\limits_{\nu } \mathop {\min }\limits_{\upsilon } \left\{ {{\rm E}_{{ \mathfrak{L}_{\upsilon \nu } }}^{ + } } \right\}\;{\text{and}}\;{\rm E}_{\pounds}^{ - } = \mathop {\max }\limits_{\nu } \mathop {\max }\limits_{\upsilon } \left\{ {{\rm E}_{{ \mathfrak{L}_{\upsilon \nu } }}^{ - } } \right\} $$

Thus,

$$ \theta = {\rm E}_{{\text{\pounds}}}^{ + } - {\rm E}_{{\text{\pounds}}}^{ - } = \mathop {\min }\limits_{\nu } \mathop {\min }\limits_{\upsilon } \left\{ {{\rm E}_{{ \mathfrak{L}_{\upsilon \nu } }}^{ + } } \right\} - \mathop {\max }\limits_{\nu } \mathop {\max }\limits_{\upsilon } \left\{ {{\rm E}_{{ \mathfrak{L}_{\upsilon \nu } }}^{ - } } \right\} = \theta \left( { \mathfrak{L}_{\upsilon \nu }^{ - } } \right), $$

Then by comparison of two IVBCFSNs, we have

$$ IVBCFSWAA\left( { \mathfrak{L}_{11} , \mathfrak{L}_{12} , \ldots , \mathfrak{L}_{{{\tilde{r}} {\tilde{s}} }} } \right) = \mathfrak{L}_{\upsilon \nu }^{ - } $$

#### Theorem 4

(Shift-invariance property): If

$$\mathfrak{L}=\left({\rm E}_{\mathfrak{L}}^{+}, {\rm E}_{\mathfrak{L}}^{-}\right)=\left(\begin{array}{c}\left[{\rm N}_{\mathfrak{L}}^{L+}, {\rm O}_{\mathfrak{L}}^{U+}\right]+\iota \left[{\rm N}_{\mathfrak{L}}^{L+}, {\rm O}_{\mathfrak{L}}^{U+}\right], \\ \left[{\rm N}_{\mathfrak{L}}^{L-}, {\rm O}_{\mathfrak{L}}^{U-}\right]+\iota \left[{\rm N}_{\mathfrak{L}}^{L-}, {\rm O}_{\mathfrak{L}}^{U-}\right]\end{array}\right)\left(\upsilon =1, 2, \dots , {\tilde{r}}; \nu =1, 2, \dots , {\tilde{s}}\right)$$

be another IVBCFSN, then

$$IVBCFSWAA\left({\mathfrak{L}}_{11}  \oplus  \mathfrak{L}, {\mathfrak{L}}_{12}  \oplus  \mathfrak{L}, \dots , {\mathfrak{L}}_{{\tilde{r}}{\tilde{s}}}  \oplus  \mathfrak{L}\right)=IVBCFSWAA\left({\mathfrak{L}}_{11}, {\mathfrak{L}}_{12}, \dots , {\mathfrak{L}}_{{\tilde{r}}{\tilde{s}}}\right)  \oplus  \mathfrak{L}$$

#### Proof

Since ${\mathfrak{L}}$ and ${\mathfrak{L}}_{\upsilon \nu }$ are IVBCFSNs. Then, we get

$$ {\mathfrak{L}} \oplus {\mathfrak{L}}_{\upsilon \nu } = \left( {\begin{array}{*{20}c} {\left[ {1 - \left( {1 - {\rm N}_{{\mathfrak{L}}}^{L + } } \right)\left( {1 - {\rm N}_{{{\mathfrak{L}}_{\upsilon \nu } }}^{L + } } \right), 1 - \left( {1 - {\rm O}_{{\mathfrak{L}}}^{U + } } \right)\left( {1 - {\rm O}_{{{\mathfrak{L}}_{\upsilon \nu } }}^{U + } } \right)} \right] + } \\ {\iota \left[ {1 - \left( {1 - {\rm N}_{{\mathfrak{L}}}^{L + } } \right)\left( {1 - {\rm N}_{{{\mathfrak{L}}_{\upsilon \nu } }}^{L + } } \right), 1 - \left( {1 - {\rm O}_{{\mathfrak{L}}}^{U + } } \right)\left( {1 - {\rm O}_{{{\mathfrak{L}}_{\upsilon \nu } }}^{U + } } \right)} \right], } \\ {\left[ { - \left| {{\rm N}_{{\mathfrak{L}}}^{L - } } \right|\left| {{\rm N}_{{{\mathfrak{L}}_{\upsilon \nu } }}^{L - } } \right|, - \left| {{\rm O}_{{\mathfrak{L}}}^{U - } } \right|\left| {{\rm O}_{{{\mathfrak{L}}_{\upsilon \nu } }}^{U - } } \right|} \right] + \iota \left[ { - \left| {{\rm N}_{{\mathfrak{L}}}^{L - } } \right|\left| {{\rm N}_{{{\mathfrak{L}}_{\upsilon \nu } }}^{L - } } \right|, - \left| {{\rm O}_{{\mathfrak{L}}}^{U - } } \right|\left| {{\rm O}_{{{\mathfrak{L}}_{\upsilon \nu } }}^{U - } } \right|} \right]} \\ \end{array} } \right) $$

Hence,

$$ \begin{aligned} &  IVBCFSWAA\left( {{\mathfrak{L}}_{{11}}  \oplus {\mathfrak{L}},{\text{~}}{\mathfrak{L}}_{{12}}  \oplus {\mathfrak{L}},{\text{~}} \ldots ,{\text{~}}{\mathfrak{L}}_{{{\tilde{r}} {\tilde{s}} }}  \oplus {\mathfrak{L}}} \right) = \oplus _{{\nu  = 1}}^{{{\tilde{s}} }} {t} _{\nu } \left( { \oplus _{{\upsilon  = 1}}^{{{\tilde{s}} }} {n} _{\upsilon } \left( {{\mathfrak{L}} \oplus {\mathfrak{L}}_{{\upsilon \nu }} } \right)} \right) \\ &    =   \left( \begin{gathered}   \left[ {1 - \mathop \prod \limits_{{\nu  = 1}}^{{{\tilde{s}} }} \left( {\mathop \prod \limits_{{\upsilon  = 1}}^{{{\tilde{r}} }} \left( {1 - {\rm N}_{{\mathfrak{L}}}^{{L + }} } \right)^{{{n} _{\upsilon } }} \left( {1 - {\rm N}_{{{\mathfrak{L}}_{{\upsilon \nu }} }}^{{L + }} } \right)^{{{n} _{\upsilon } }} } \right)^{{{t} _{\nu } }} ,~1 - \mathop \prod \limits_{{\nu  = 1}}^{{{\tilde{s}} }} \left( {\mathop \prod \limits_{{\upsilon  = 1}}^{{{\tilde{r}} }} \left( {1 - {\rm O}_{{\mathfrak{L}}}^{{U + }} } \right)^{{{n} _{\upsilon } }} \left( {1 - {\rm O}_{{{\mathfrak{L}}_{{\upsilon \nu }} }}^{{U + }} } \right)^{{{n} _{\upsilon } }} } \right)^{{{t} _{\nu } }} } \right] +  \hfill \\   \iota \left[ {1 - \mathop \prod \limits_{{\nu  = 1}}^{{{\tilde{s}} }} \left( {\mathop \prod \limits_{{\upsilon  = 1}}^{{{\tilde{r}} }} \left( {1 - {\rm N}_{{\mathfrak{L}}}^{{L + }} } \right)^{{{n} _{\upsilon } }} \left( {1 - {\rm N}_{{{\mathfrak{L}}_{{\upsilon \nu }} }}^{{L + }} } \right)^{{{n} _{\upsilon } }} } \right)^{{{t} _{\nu } }} ,~1 - \mathop \prod \limits_{{\nu  = 1}}^{{{\tilde{s}} }} \left( {\mathop \prod \limits_{{\upsilon  = 1}}^{{{\tilde{r}} }} \left( {1 - {\rm O}_{{\mathfrak{L}}}^{{U + }} } \right)^{{{n} _{\upsilon } }} \left( {1 - {\rm O}_{{{\mathfrak{L}}_{{\upsilon \nu }} }}^{{U + }} } \right)^{{{n} _{\upsilon } }} } \right)^{{{t} _{\nu } }} } \right], \hfill \\   \begin{array}{*{20}c}    {\left[ { - \mathop \prod \limits_{{\nu  = 1}}^{{{\tilde{s}} }} \left( {\mathop \prod \limits_{{\upsilon  = 1}}^{{{\tilde{r}} }} \left| {{\rm N}_{{\mathfrak{L}}}^{{L - }} } \right|^{{{n} _{\upsilon } }} \left| {{\rm N}_{{{\mathfrak{L}}_{{\upsilon \nu }} }}^{{L - }} } \right|^{{{n} _{\upsilon } }} } \right)^{{{t} _{\nu } }} ,~ - \mathop \prod \limits_{{\nu  = 1}}^{{{\tilde{s}} }} \left( {\mathop \prod \limits_{{\upsilon  = 1}}^{{{\tilde{r}} }} \left| {{\rm O}_{{\mathfrak{L}}}^{{U - }} } \right|^{{{n} _{\upsilon } }} \left| {{\rm O}_{{{\mathfrak{L}}_{{\upsilon \nu }} }}^{{U - }} } \right|^{{{n} _{\upsilon } }} } \right)^{{{t} _{\nu } }} } \right] + }  \\    {\iota \left[ { - \mathop \prod \limits_{{\nu  = 1}}^{{{\tilde{s}} }} \left( {\mathop \prod \limits_{{\upsilon  = 1}}^{{{\tilde{r}} }} \left| {{\rm N}_{{\mathfrak{L}}}^{{L - }} } \right|^{{{n} _{\upsilon } }} \left| {{\rm N}_{{{\mathfrak{L}}_{{\upsilon \nu }} }}^{{L - }} } \right|^{{{n} _{\upsilon } }} } \right)^{{{t} _{\nu } }} ,~ - \mathop \prod \limits_{{\nu  = 1}}^{{{\tilde{s}} }} \left( {\mathop \prod \limits_{{\upsilon  = 1}}^{{{\tilde{r}} }} \left| {{\rm O}_{{\mathfrak{L}}}^{{U - }} } \right|^{{{n} _{\upsilon } }} \left| {{\rm O}_{{{\mathfrak{L}}_{{\upsilon \nu }} }}^{{U - }} } \right|^{{{n} _{\upsilon } }} } \right)^{{{t} _{\nu } }} } \right]}  \\   \end{array}  \hfill \\  \end{gathered}  \right) \\ &    =   \left( \begin{gathered}   \left[ {1 - \left( {1 - {\rm N}_{{\mathfrak{L}}}^{{L + }} } \right)\mathop \prod \limits_{{\nu  = 1}}^{{{\tilde{s}} }} \left( {\mathop \prod \limits_{{\upsilon  = 1}}^{{{\tilde{r}} }} \left( {1 - {\rm N}_{{{\mathfrak{L}}_{{\upsilon \nu }} }}^{{L + }} } \right)^{{{n} _{\upsilon } }} } \right)^{{{t} _{\nu } }} ,~1 - \left( {1 - {\rm O}_{{\mathfrak{L}}}^{{U + }} } \right)\mathop \prod \limits_{{\nu  = 1}}^{{{\tilde{s}} }} \left( {\mathop \prod \limits_{{\upsilon  = 1}}^{{{\tilde{r}} }} \left( {1 - {\rm O}_{{{\mathfrak{L}}_{{\upsilon \nu }} }}^{{U + }} } \right)^{{{n} _{\upsilon } }} } \right)^{{{t} _{\nu } }} } \right] +  \hfill \\   \iota \left[ {1 - \left( {1 - {\rm N}_{{\mathfrak{L}}}^{{L + }} } \right)\mathop \prod \limits_{{\nu  = 1}}^{{{\tilde{s}} }} \left( {\mathop \prod \limits_{{\upsilon  = 1}}^{{{\tilde{r}} }} \left( {1 - {\rm N}_{{{\mathfrak{L}}_{{\upsilon \nu }} }}^{{L + }} } \right)^{{{n} _{\upsilon } }} } \right)^{{{t} _{\nu } }} ,~1 - \left( {1 - {\rm O}_{{\mathfrak{L}}}^{{U + }} } \right)\mathop \prod \limits_{{\nu  = 1}}^{{{\tilde{s}} }} \left( {\mathop \prod \limits_{{\upsilon  = 1}}^{{{\tilde{r}} }} \left( {1 - {\rm O}_{{{\mathfrak{L}}_{{\upsilon \nu }} }}^{{U + }} } \right)^{{{n} _{\upsilon } }} } \right)^{{{t} _{\nu } }} } \right], \hfill \\   \begin{array}{*{20}c}    {\left[ { - {\rm N}_{{\mathfrak{L}}}^{{L - }} \mathop \prod \limits_{{\nu  = 1}}^{{{\tilde{s}} }} \left( {\mathop \prod \limits_{{\upsilon  = 1}}^{{{\tilde{r}} }} \left| {{\rm N}_{{{\mathfrak{L}}_{{\upsilon \nu }} }}^{{L - }} } \right|^{{{n} _{\upsilon } }} } \right)^{{{t} _{\nu } }} ,~ - {\rm O}_{{\mathfrak{L}}}^{{U - }} \mathop \prod \limits_{{\nu  = 1}}^{{{\tilde{s}} }} \left( {\mathop \prod \limits_{{\upsilon  = 1}}^{{{\tilde{r}} }} \left| {{\rm O}_{{{\mathfrak{L}}_{{\upsilon \nu }} }}^{{U - }} } \right|^{{{n} _{\upsilon } }} } \right)^{{{t} _{\nu } }} } \right] + }  \\    {\iota \left[ { - {\rm N}_{{\mathfrak{L}}}^{{L - }} \mathop \prod \limits_{{\nu  = 1}}^{{{\tilde{s}} }} \left( {\mathop \prod \limits_{{\upsilon  = 1}}^{{{\tilde{r}} }} \left| {{\rm N}_{{{\mathfrak{L}}_{{\upsilon \nu }} }}^{{L - }} } \right|^{{{n} _{\upsilon } }} } \right)^{{{t} _{\nu } }} ,~ - {\rm O}_{{\mathfrak{L}}}^{{U - }} \mathop \prod \limits_{{\nu  = 1}}^{{{\tilde{s}} }} \left( {\mathop \prod \limits_{{\upsilon  = 1}}^{{{\tilde{r}} }} \left| {{\rm O}_{{{\mathfrak{L}}_{{\upsilon \nu }} }}^{{U - }} } \right|^{{{n} _{\upsilon } }} } \right)^{{{t} _{\nu } }} } \right]}  \\   \end{array}  \hfill \\  \end{gathered}  \right) \\  &   =   \left( \begin{gathered}   \left[ {1 - \mathop \prod \limits_{{\nu  = 1}}^{{{\tilde{s}} }} \left( {\mathop \prod \limits_{{\upsilon  = 1}}^{{{\tilde{r}} }} \left( {1 - {\rm N}_{{{\mathfrak{L}}_{{\upsilon \nu }} }}^{{L + }} } \right)^{{{n} _{\upsilon } }} } \right)^{{{t} _{\nu } }} ,~1 - \mathop \prod \limits_{{\nu  = 1}}^{{{\tilde{s}} }} \left( {\mathop \prod \limits_{{\upsilon  = 1}}^{{{\tilde{r}} }} \left( {1 - {\rm O}_{{{\mathfrak{L}}_{{\upsilon \nu }} }}^{{U + }} } \right)^{{{n} _{\upsilon } }} } \right)^{{{t} _{\nu } }} } \right] +  \hfill \\   \iota \left[ {1 - \mathop \prod \limits_{{\nu  = 1}}^{{{\tilde{s}} }} \left( {\mathop \prod \limits_{{\upsilon  = 1}}^{{{\tilde{r}} }} \left( {1 - {\rm N}_{{{\mathfrak{L}}_{{\upsilon \nu }} }}^{{L + }} } \right)^{{{n} _{\upsilon } }} } \right)^{{{t} _{\nu } }} ,~1 - \mathop \prod \limits_{{\nu  = 1}}^{{{\tilde{s}} }} \left( {\mathop \prod \limits_{{\upsilon  = 1}}^{{{\tilde{r}} }} \left( {1 - {\rm O}_{{{\mathfrak{L}}_{{\upsilon \nu }} }}^{{U + }} } \right)^{{{n} _{\upsilon } }} } \right)^{{{t} _{\nu } }} } \right], \hfill \\   \begin{array}{*{20}c}    {\left[ { - \mathop \prod \limits_{{\nu  = 1}}^{{{\tilde{s}} }} \left( {\mathop \prod \limits_{{\upsilon  = 1}}^{{{\tilde{r}} }} \left| {{\rm N}_{{{\mathfrak{L}}_{{\upsilon \nu }} }}^{{L - }} } \right|^{{{n} _{\upsilon } }} } \right)^{{{t} _{\nu } }} ,~ - \mathop \prod \limits_{{\nu  = 1}}^{{{\tilde{s}} }} \left( {\mathop \prod \limits_{{\upsilon  = 1}}^{{{\tilde{r}} }} \left| {{\rm O}_{{{\mathfrak{L}}_{{\upsilon \nu }} }}^{{U - }} } \right|^{{{n} _{\upsilon } }} } \right)^{{{t} _{\nu } }} } \right] + }  \\    {\iota \left[ { - \mathop \prod \limits_{{\nu  = 1}}^{{{\tilde{s}} }} \left( {\mathop \prod \limits_{{\upsilon  = 1}}^{{{\tilde{r}} }} \left| {{\rm N}_{{{\mathfrak{L}}_{{\upsilon \nu }} }}^{{L - }} } \right|^{{{n} _{\upsilon } }} } \right)^{{{t} _{\nu } }} ,~ - \mathop \prod \limits_{{\nu  = 1}}^{{{\tilde{s}} }} \left( {\mathop \prod \limits_{{\upsilon  = 1}}^{{{\tilde{r}} }} \left| {{\rm O}_{{{\mathfrak{L}}_{{\upsilon \nu }} }}^{{U - }} } \right|^{{{n} _{\upsilon } }} } \right)^{{{t} _{\nu } }} } \right]}  \\   \end{array}  \hfill \\  \end{gathered}  \right) \oplus \left( {\begin{array}{*{20}c}    {\left[ {{\rm N}_{{\mathfrak{L}}}^{{L + }} ,~{\rm O}_{{\mathfrak{L}}}^{{U + }} } \right] + \iota \left[ {{\rm N}_{{\mathfrak{L}}}^{{L + }} ,~{\rm O}_{{\mathfrak{L}}}^{{U + }} } \right],~}  \\    {\left[ {{\rm N}_{{\mathfrak{L}}}^{{L - }} ,~{\rm O}_{{\mathfrak{L}}}^{{U - }} } \right] + \iota \left[ {{\rm N}_{{\mathfrak{L}}}^{{L - }} ,~{\rm 
O}_{{\mathfrak{L}}}^{{U - }} } \right]}  \\   \end{array} } \right) \\ &    =   IVBCFSWAA\left( {{\mathfrak{L}}_{{11}} ,~{\mathfrak{L}}_{{12}} ,~ \ldots ,~{\mathfrak{L}}_{{{\tilde{r}} {\tilde{s}} }} } \right) \oplus {\mathfrak{L}} \\  \end{aligned}   $$

#### Theorem 5

**(Homogeneity property):** For any real number $\chi > 0$, we have

$$ IVBCFSWAA\left( {\chi {\mathfrak{L}}_{11} , \chi {\mathfrak{L}}_{12} , \ldots , \chi {\mathfrak{L}}_{{{\tilde{r}} {\tilde{s}} }} } \right) = \chi IVBCFSWAA\left( {{\mathfrak{L}}_{11} , {\mathfrak{L}}_{12} , \ldots , {\mathfrak{L}}_{{{\tilde{r}} {\tilde{s}} }} } \right) $$

#### Proof

Suppose

$${\mathfrak{L}}_{\upsilon \nu } = \left( {{\rm E}_{{{\mathfrak{L}}_{\upsilon \nu } }}^{ + } , {\rm E}_{{{\mathfrak{L}}_{\upsilon \nu } }}^{ - } } \right) = \left( {\begin{array}{*{20}c} {\left[ {{\rm N}_{{{\mathfrak{L}}_{\upsilon \nu } }}^{L + } , {\rm O}_{{{\mathfrak{L}}_{\upsilon \nu } }}^{U + } } \right] + \iota \left[ {{\rm N}_{{{\mathfrak{L}}_{\upsilon \nu } }}^{L + } , {\rm O}_{{{\mathfrak{L}}_{\upsilon \nu } }}^{U + } } \right], } \\ {\left[ {{\rm N}_{{{\mathfrak{L}}_{\upsilon \nu } }}^{L - } , {\rm O}_{{{\mathfrak{L}}_{\upsilon \nu } }}^{U - } } \right] + \iota \left[ {{\rm N}_{{{\mathfrak{L}}_{\upsilon \nu } }}^{L - } , {\rm O}_{{{\mathfrak{L}}_{\upsilon \nu } }}^{U - } } \right]} \\ \end{array} } \right)\left( {\upsilon = 1, 2, \ldots , {\tilde{r}} ; \nu = 1, 2, \ldots , {\tilde{s}} } \right)$$

be the collection of IVBCFSNs and $\chi > 0$ for any real value. Then,

$$ \chi {\mathfrak{L}} = \left( {\begin{array}{*{20}c} {\left[ {1 - \left( {1 - {\rm N}_{{{\mathfrak{L}}_{\upsilon \nu } }}^{L + } } \right)^{\chi } , 1 - \left( {1 - {\rm O}_{{{\mathfrak{L}}_{\upsilon \nu } }}^{U + } } \right)^{\chi } } \right] + } \\ {\iota \left[ {1 - \left( {1 - {\rm N}_{{{\mathfrak{L}}_{\upsilon \nu } }}^{L + } } \right)^{\chi } , 1 - \left( {1 - {\rm O}_{{{\mathfrak{L}}_{\upsilon \nu } }}^{U + } } \right)^{\chi } } \right], } \\ {\left[ { - \left| {{\rm N}_{{{\mathfrak{L}}_{\upsilon \nu } }}^{L - } } \right|^{\chi } , - \left| {{\rm O}_{{{\mathfrak{L}}_{\upsilon \nu } }}^{U - } } \right|^{\chi } } \right] + \iota \left[ { - \left| {{\rm N}_{{{\mathfrak{L}}_{\upsilon \nu } }}^{L - } } \right|^{\chi } , - \left| {{\rm O}_{{{\mathfrak{L}}_{\upsilon \nu } }}^{U - } } \right|^{\chi } } \right]} \\ \end{array} } \right) $$

Thus,

$$  \begin{aligned}  &  IVBCFSWAA\left( {\chi {\mathfrak{L}}_{{11}} ,~\chi {\mathfrak{L}}_{{12}} ,~ \ldots ,~\chi {\mathfrak{L}}_{{{\tilde{r}}{\tilde{s}} }} } \right) \\ & =  \left( \begin{gathered}   \iota \left[ {1 - \mathop \prod \limits_{{\nu  = 1}}^{{{\tilde{s}} }} \left( {\mathop \prod \limits_{{\upsilon  = 1}}^{{{\tilde{r}}}} \left( {1 - {\rm N}_{{{\mathfrak{L}}_{{\upsilon \nu }} }}^{{L + }} } \right)^{{\chi {n} _{\upsilon } }} } \right)^{{{t} _{\nu } }} ,~1 - \mathop \prod \limits_{{\nu  = 1}}^{{{\tilde{s}} }} \left( {\mathop \prod \limits_{{\upsilon  = 1}}^{{{\tilde{r}}}} \left( {1 - {\rm O}_{{{\mathfrak{L}}_{{\upsilon \nu }} }}^{{U + }} } \right)^{{\chi {n} _{\upsilon } }} } \right)^{{{t} _{\nu } }} } \right], \hfill \\   \left[ {1 - \mathop \prod \limits_{{\nu  = 1}}^{{{\tilde{s}} }} \left( {\mathop \prod \limits_{{\upsilon  = 1}}^{{{\tilde{r}}}} \left( {1 - {\rm N}_{{{\mathfrak{L}}_{{\upsilon \nu }} }}^{{L + }} } \right)^{{\chi {n} _{\upsilon } }} } \right)^{{{t} _{\nu } }} ,~1 - \mathop \prod \limits_{{\nu  = 1}}^{{{\tilde{s}} }} \left( {\mathop \prod \limits_{{\upsilon  = 1}}^{{{\tilde{r}}}} \left( {1 - {\rm O}_{{{\mathfrak{L}}_{{\upsilon \nu }} }}^{{U + }} } \right)^{{\chi {n} _{\upsilon } }} } \right)^{{{t} _{\nu } }} } \right] +  \hfill \\   \left[ { - \mathop \prod \limits_{{\nu  = 1}}^{{{\tilde{s}} }} \left( {\mathop \prod \limits_{{\upsilon  = 1}}^{{{\tilde{r}}}} \left| {{\rm N}_{{{\mathfrak{L}}_{{\upsilon \nu }} }}^{{L - }} } \right|^{{\chi {n} _{\upsilon } }} } \right)^{{{t} _{\nu } }} ,~ - \mathop \prod \limits_{{\nu  = 1}}^{{{\tilde{s}} }} \left( {\mathop \prod \limits_{{\upsilon  = 1}}^{{{\tilde{r}}}} \left| {{\rm O}_{{{\mathfrak{L}}_{{\upsilon \nu }} }}^{{U - }} } \right|^{{\chi {n} _{\upsilon } }} } \right)^{{{t} _{\nu } }} } \right] +  \hfill \\   \iota \left[ { - \mathop \prod \limits_{{\nu  = 1}}^{{{\tilde{s}} }} \left( {\mathop \prod \limits_{{\upsilon  = 1}}^{{{\tilde{r}}}} \left| {{\rm N}_{{{\mathfrak{L}}_{{\upsilon \nu }} }}^{{L - }} } \right|^{{\chi {n} _{\upsilon } }} } \right)^{{{t} _{\nu } }} ,~ - \mathop \prod \limits_{{\nu  = 1}}^{{{\tilde{s}} }} \left( {\mathop \prod \limits_{{\upsilon  = 1}}^{{{\tilde{r}}}} \left| {{\rm O}_{{{\mathfrak{L}}_{{\upsilon \nu }} }}^{{U - }} } \right|^{{\chi {n} _{\upsilon } }} } \right)^{{{t} _{\nu } }} } \right] \hfill \\  \end{gathered}  \right) \\  &    =  \left( \begin{gathered}   \left[ {1 - \left( {\mathop \prod \limits_{{\nu  = 1}}^{{{\tilde{s}} }} \left( {\mathop \prod \limits_{{\upsilon  = 1}}^{{{\tilde{r}}}} \left( {1 - {\rm N}_{{{\mathfrak{L}}_{{\upsilon \nu }} }}^{{L + }} } \right)^{{{n} _{\upsilon } }} } \right)^{{{t} _{\nu } }} } \right)^{\chi } ,~1 - \left( {\mathop \prod \limits_{{\nu  = 1}}^{{{\tilde{s}} }} \left( {\mathop \prod \limits_{{\upsilon  = 1}}^{{{\tilde{r}}}} \left( {1 - {\rm O}_{{{\mathfrak{L}}_{{\upsilon \nu }} }}^{{U + }} } \right)^{{{n} _{\upsilon } }} } \right)^{{{t} _{\nu } }} } \right)^{\chi } } \right] +  \hfill \\   \iota \left[ {1 - \left( {\mathop \prod \limits_{{\nu  = 1}}^{{{\tilde{s}} }} \left( {\mathop \prod \limits_{{\upsilon  = 1}}^{{{\tilde{r}}}} \left( {1 - {\rm N}_{{{\mathfrak{L}}_{{\upsilon \nu }} }}^{{L + }} } \right)^{{{n} _{\upsilon } }} } \right)^{{{t} _{\nu } }} } \right)^{\chi } ,~1 - \left( {\mathop \prod \limits_{{\nu  = 1}}^{{{\tilde{s}} }} \left( {\mathop \prod \limits_{{\upsilon  = 1}}^{{{\tilde{r}}}} \left( {1 - {\rm O}_{{{\mathfrak{L}}_{{\upsilon \nu }} }}^{{U + }} } \right)^{{{n} _{\upsilon } }} } \right)^{{{t} _{\nu } }} } \right)^{\chi } } \right], \hfill \\   \left[ { - \left( {\mathop \prod \limits_{{\nu  = 1}}^{{{\tilde{s}} }} \left( {\mathop \prod \limits_{{\upsilon  = 1}}^{{{\tilde{r}}}} \left| {{\rm N}_{{{\mathfrak{L}}_{{\upsilon \nu }} }}^{{L - }} } \right|^{{{n} _{\upsilon } }} } \right)^{{{t} _{\nu } }} } \right)^{\chi } ,~ - \left( {\mathop \prod \limits_{{\nu  = 1}}^{{{\tilde{s}} }} \left( {\mathop \prod \limits_{{\upsilon  = 1}}^{{{\tilde{r}}}} \left| {{\rm O}_{{{\mathfrak{L}}_{{\upsilon \nu }} }}^{{U - }} } \right|^{{{n} _{\upsilon } }} } \right)^{{{t} _{\nu } }} } \right)^{\chi } } \right] +  \hfill \\   \iota \left[ { - \left( {\mathop \prod \limits_{{\nu  = 1}}^{{{\tilde{s}} }} \left( {\mathop \prod \limits_{{\upsilon  = 1}}^{{{\tilde{r}}}} \left| {{\rm N}_{{{\mathfrak{L}}_{{\upsilon \nu }} }}^{{L - }} } \right|^{{{n} _{\upsilon } }} } \right)^{{{t} _{\nu } }} } \right)^{\chi } ,~ - \left( {\mathop \prod \limits_{{\nu  = 1}}^{{{\tilde{s}} }} \left( {\mathop \prod \limits_{{\upsilon  = 1}}^{{{\tilde{r}}}} \left| {{\rm O}_{{{\mathfrak{L}}_{{\upsilon \nu }} }}^{{U - }} } \right|^{{{n} _{\upsilon } }} } \right)^{{{t} _{\nu } }} } \right)^{\chi } } \right] \hfill \\  \end{gathered}  \right) \\ &      =  \chi IVBCFSWAA\left( {{\mathfrak{L}}_{{11}} ,~{\mathfrak{L}}_{{12}} ,~ \ldots ,~{\mathfrak{L}}_{{{\tilde{r}}{\tilde{s}} }} } \right) \\  \end{aligned}   $$

### Interval-valued bipolar complex fuzzy soft geometric aggregation operator

#### Definition 25

Suppose

$${\mathfrak{L}}_{\upsilon \nu } = \left( {{\rm E}_{{{\mathfrak{L}}_{\upsilon \nu } }}^{ + } , {\rm E}_{{{\mathfrak{L}}_{\upsilon \nu } }}^{ - } } \right) = \left( {\imath_{\upsilon } , \begin{array}{*{20}c} {\left[ {{\rm N}_{{{\mathfrak{L}}_{\upsilon \nu } }}^{L + } , {\rm O}_{{{\mathfrak{L}}_{\upsilon \nu } }}^{U + } } \right] + \iota \left[ {{\rm N}_{{{\mathfrak{L}}_{\upsilon \nu } }}^{L + } , {\rm O}_{{{\mathfrak{L}}_{\upsilon \nu } }}^{U + } } \right], } \\ {\left[ {{\rm N}_{{{\mathfrak{L}}_{\upsilon \nu } }}^{L - } , {\rm O}_{{{\mathfrak{L}}_{\upsilon \nu } }}^{U - } } \right] + \iota \left[ {{\rm N}_{{{\mathfrak{L}}_{\upsilon \nu } }}^{L - } , {\rm O}_{{{\mathfrak{L}}_{\upsilon \nu } }}^{U - } } \right]} \\ \end{array} } \right)\left( {\upsilon = 1, 2, \ldots , {\tilde{r}} ; \nu = 1, 2, \ldots , {\tilde{s}} } \right)$$

be the collection of IVBCFSNs and let ${n}_{\upsilon , }{t}_{\nu }$ are the weight vectors (WVs) for experts ${\iota }_{\upsilon }^{\prime}s$ and ${j}_{\nu }^{\prime}s$ respectively, holding $n_{\upsilon } \ge 0, t_{\nu } \ge 0$ such that $\mathop \sum \nolimits_{\upsilon = 1}^{{{\tilde{r}} }} n_{\upsilon } = 1$ and $\mathop \sum \nolimits_{\nu = 1}^{{{\tilde{s}} }} t_{\nu } = 1$. The IVBCFS weighted geometric aggregation (IVBCFSWGA) operator is the function $IVBCFSWGA:{\mathfrak{L}}^{n}\to \mathfrak{L}$ such that

15 $$ IVBCFSWGA\left( {{\mathfrak{L}}_{11} , {\mathfrak{L}}_{12} , \ldots , {\mathfrak{L}}_{{{\tilde{r}} {\tilde{s}} }} } \right) = \oplus_{\nu = 1}^{{{\tilde{s}} }} t_{\nu } \left( { \oplus_{\upsilon = 1}^{{{\tilde{r}} }} {\mathfrak{L}}_{\upsilon \nu }^{{n_{\upsilon } }} } \right) $$

#### Theorem 6

Suppose

$${\mathfrak{L}}_{\upsilon \nu } = \left( {{\rm E}_{{{\mathfrak{L}}_{\upsilon \nu } }}^{ + } , {\rm E}_{{{\mathfrak{L}}_{\upsilon \nu } }}^{ - } } \right) = \left( {\imath_{\upsilon } , \begin{array}{*{20}c} {\left[ {{\rm N}_{{{\mathfrak{L}}_{\upsilon \nu } }}^{L + } , {\rm O}_{{{\mathfrak{L}}_{\upsilon \nu } }}^{U + } } \right] + \iota \left[ {{\rm N}_{{{\mathfrak{L}}_{\upsilon \nu } }}^{L + } , {\rm O}_{{{\mathfrak{L}}_{\upsilon \nu } }}^{U + } } \right], } \\ {\left[ {{\rm N}_{{{\mathfrak{L}}_{\upsilon \nu } }}^{L - } , {\rm O}_{{{\mathfrak{L}}_{\upsilon \nu } }}^{U - } } \right] + \iota \left[ {{\rm N}_{{{\mathfrak{L}}_{\upsilon \nu } }}^{L - } , {\rm O}_{{{\mathfrak{L}}_{\upsilon \nu } }}^{U - } } \right]} \\ \end{array} } \right)\left( {\upsilon = 1, 2, \ldots , {\tilde{r}} ; \nu = 1, 2, \ldots , {\tilde{s}} } \right)$$

be the assortment of IVBCFSNs, then aggregated value of them usage the IVBCFSWAA operator is also a IVBCFSN, and

16 $$ IVBCFSWAA\left( {{\mathfrak{L}}_{11} , {\mathfrak{L}}_{12} , \ldots , {\mathfrak{L}}_{{{\tilde{r}} {\tilde{s}} }} } \right) = \left( \begin{gathered} \left[ {\mathop \prod \limits_{\nu = 1}^{{{\tilde{s}} }} \left( {\mathop \prod \limits_{\upsilon = 1}^{{{\tilde{r}} }} \left( {{\rm N}_{{{\mathfrak{L}}_{\upsilon \nu } }}^{L + } } \right)^{{n_{\upsilon } }} } \right)^{{t_{\nu } }} , \mathop \prod \limits_{\nu = 1}^{{{\tilde{s}} }} \left( {\mathop \prod \limits_{\upsilon = 1}^{{{\tilde{r}} }} \left( {{\rm O}_{{{\mathfrak{L}}_{\upsilon \nu } }}^{U + } } \right)^{{n_{\upsilon } }} } \right)^{{t_{\nu } }} } \right] \hfill \\ + \iota \left[ {\mathop \prod \limits_{\nu = 1}^{{{\tilde{s}} }} \left( {\mathop \prod \limits_{\upsilon = 1}^{{{\tilde{r}} }} \left( {{\rm N}_{{{\mathfrak{L}}_{\upsilon \nu } }}^{L + } } \right)^{{n_{\upsilon } }} } \right)^{{t_{\nu } }} , \mathop \prod \limits_{\nu = 1}^{{{\tilde{s}} }} \left( {\mathop \prod \limits_{\upsilon = 1}^{{{\tilde{r}} }} \left( {{\rm O}_{{{\mathfrak{L}}_{\upsilon \nu } }}^{U + } } \right)^{{n_{\upsilon } }} } \right)^{{t_{\nu } }} } \right], \hfill \\ \left[ { - 1 + \mathop \prod \limits_{\nu = 1}^{{{\tilde{s}} }} \left( {\mathop \prod \limits_{\upsilon = 1}^{{{\tilde{r}} }} \left| {{\rm N}_{{{\mathfrak{L}}_{\upsilon \nu } }}^{L - } } \right|^{{n_{\upsilon } }} } \right)^{{t_{\nu } }} , - 1 + \mathop \prod \limits_{\nu = 1}^{{{\tilde{s}} }} \left( {\mathop \prod \limits_{\upsilon = 1}^{{{\tilde{r}} }} \left| {{\rm O}_{{{\mathfrak{L}}_{\upsilon \nu } }}^{U - } } \right|^{{n_{\upsilon } }} } \right)^{{t_{\nu } }} } \right] \hfill \\ + \iota \left[ { - 1 + \mathop \prod \limits_{\nu = 1}^{{{\tilde{s}} }} \left( {\mathop \prod \limits_{\upsilon = 1}^{{{\tilde{r}} }} \left| {{\rm N}_{{{\mathfrak{L}}_{\upsilon \nu } }}^{L - } } \right|^{{n_{\upsilon } }} } \right)^{{t_{\nu } }} , - 1 + \mathop \prod \limits_{\nu = 1}^{{{\tilde{s}} }} \left( {\mathop \prod \limits_{\upsilon = 1}^{{{\tilde{r}} }} \left| {{\rm O}_{{{\mathfrak{L}}_{\upsilon \nu } }}^{U - } } \right|^{{n_{\upsilon } }} } \right)^{{t_{\nu } }} } \right] \hfill \\ \end{gathered} \right) $$

#### Proof

As like to Theorem 1.

Now we desire to explain the following properties by the usage the operator IVBCFSWGA.

#### Theorem 7

(Idempotency): Suppose

$${\mathfrak{L}}_{\upsilon \nu } = \left( {{\rm E}_{{{\mathfrak{L}}_{\upsilon \nu } }}^{ + } , {\rm E}_{{{\mathfrak{L}}_{\upsilon \nu } }}^{ - } } \right) = \left( {\begin{array}{*{20}c} {\left[ {{\rm N}_{{{\mathfrak{L}}_{\upsilon \nu } }}^{L + } , {\rm O}_{{{\mathfrak{L}}_{\upsilon \nu } }}^{U + } } \right] + \iota \left[ {{\rm N}_{{{\mathfrak{L}}_{\upsilon \nu } }}^{L + } , {\rm O}_{{{\mathfrak{L}}_{\upsilon \nu } }}^{U + } } \right], } \\ {\left[ {{\rm N}_{{{\mathfrak{L}}_{\upsilon \nu } }}^{L - } , {\rm O}_{{{\mathfrak{L}}_{\upsilon \nu } }}^{U - } } \right] + \iota \left[ {{\rm N}_{{{\mathfrak{L}}_{\upsilon \nu } }}^{L - } , {\rm O}_{{{\mathfrak{L}}_{\upsilon \nu } }}^{U - } } \right]} \\ \end{array} } \right)\left( {\upsilon = 1, 2, \ldots , {\tilde{r}} ; \nu = 1, 2, \ldots , {\tilde{s}} } \right)$$

be the collection of IVBCFSNs, then using the IVBCFSWGA operator is also a IVBCFSNs that are all equal, i.e., ${\mathfrak{L}}_{\upsilon \nu } = {\mathfrak{L}} \forall \upsilon , \nu$ then

17 $$ IVBCFSWGA\left( {{\mathfrak{L}}_{11} , {\mathfrak{L}}_{12} , \ldots , {\mathfrak{L}}_{{{\tilde{r}} {\tilde{s}} }} } \right) = {\mathfrak{L}} $$

#### Proof

As like to Theorem 2.

#### Theorem 8

(Boundedness): Suppose

$${\mathfrak{L}}_{\upsilon \nu } = \left( {{\rm E}_{{{\mathfrak{L}}_{\upsilon \nu } }}^{ + } , {\rm E}_{{{\mathfrak{L}}_{\upsilon \nu } }}^{ - } } \right) = \left( {\begin{array}{*{20}c} {\left[ {{\rm N}_{{{\mathfrak{L}}_{\upsilon \nu } }}^{L + } , {\rm O}_{{{\mathfrak{L}}_{\upsilon \nu } }}^{U + } } \right] + \iota \left[ {{\rm N}_{{{\mathfrak{L}}_{\upsilon \nu } }}^{L + } , {\rm O}_{{{\mathfrak{L}}_{\upsilon \nu } }}^{U + } } \right], } \\ {\left[ {{\rm N}_{{{\mathfrak{L}}_{\upsilon \nu } }}^{L - } , {\rm O}_{{{\mathfrak{L}}_{\upsilon \nu } }}^{U - } } \right] + \iota \left[ {{\rm N}_{{{\mathfrak{L}}_{\upsilon \nu } }}^{L - } , {\rm O}_{{{\mathfrak{L}}_{\upsilon \nu } }}^{U - } } \right]} \\ \end{array} } \right)\;\left( {\upsilon = 1, 2, \ldots , {\tilde{r}} ; \nu = 1, 2, \ldots , {\tilde{s}} } \right)$$

be the collection of IVBCFSNs.

$${\mathfrak{L}}_{\upsilon \nu }^{-}=\left(\underset{\nu }{{\text{min}}}\,\underset{\upsilon }{{\text{min}}}\,\left\{{\rm E}_{{\mathfrak{L}}_{\upsilon \nu }}^{+}\right\}, \underset{\nu }{{\text{max}}}\,\underset{\upsilon }{{\text{max}}}\left\{{\rm E}_{{\mathfrak{L}}_{\upsilon \nu }}^{-}\right\}\right)=\left(\begin{array}{c}\left[\underset{\nu }{{\text{min}}}\,\underset{\upsilon }{{\text{min}}}\,\left\{{\rm N}_{{\mathfrak{L}}_{\upsilon \nu }}^{L+}\right\}, \underset{\nu }{{\text{min}}}\,\underset{\upsilon }{{\text{min}}}\,\left\{{\rm O}_{{\mathfrak{L}}_{\upsilon \nu }}^{U+}\right\}\right]\\ +\iota \left[\underset{\nu }{{\text{min}}}\,\underset{\upsilon }{{\text{min}}}\,\left\{{\rm N}_{{\mathfrak{L}}_{\upsilon \nu }}^{L+}\right\}, \underset{\nu }{{\text{min}}}\,\underset{\upsilon }{{\text{min}}}\,\left\{{\rm O}_{{\mathfrak{L}}_{\upsilon \nu }}^{U+}\right\}\right], \\ \left[\underset{\nu }{{\text{max}}}\,\underset{\upsilon }{{\text{max}}}\left\{{\rm N}_{{\mathfrak{L}}_{\upsilon \nu }}^{L-}\right\}, \underset{\nu }{{\text{max}}}\,\underset{\upsilon }{{\text{max}}}\left\{{\rm O}_{{\mathfrak{L}}_{\upsilon \nu }}^{U-}\right\}\right]+\\ \iota \left[\underset{\nu }{{\text{max}}}\,\underset{\upsilon }{{\text{max}}}\left\{{\rm N}_{{\mathfrak{L}}_{\upsilon \nu }}^{L-}\right\}, \underset{\nu }{{\text{max}}}\,\underset{\upsilon }{{\text{max}}}\left\{{\rm O}_{{\mathfrak{L}}_{\upsilon \nu }}^{U-}\right\}\right]\end{array}\right)$$

And $${\mathfrak{L}}_{\upsilon \nu }^{+}=\left(\underset{\nu }{{\text{max}}}\,\underset{\upsilon }{{\text{max}}}\left\{{\rm E}_{{\mathfrak{L}}_{\upsilon \nu }}^{+}\right\}, \underset{\nu }{{\text{min}}}\,\underset{\upsilon }{{\text{min}}}\,\left\{{\rm E}_{{\mathfrak{L}}_{\upsilon \nu }}^{-}\right\}\right)=\left(\begin{array}{c}\left[\underset{\nu }{{\text{max}}}\,\underset{\upsilon }{{\text{max}}}\left\{{\rm N}_{{\mathfrak{L}}_{\upsilon \nu }}^{L+}\right\}, \underset{\mathit{\nu }}{{\text{max}}}\,\underset{\upsilon }{{\text{max}}}\left\{{\rm O}_{{\mathfrak{L}}_{\upsilon \nu }}^{U+}\right\}\right]+\\ \iota \left[\underset{\nu }{{\text{max}}}\,\underset{\upsilon }{{\text{max}}}\left\{{\rm N}_{{\mathfrak{L}}_{\upsilon \nu }}^{L+}\right\}, \underset{\mathit{\nu }}{{\text{max}}}\,\underset{\upsilon }{{\text{max}}}\left\{{\rm O}_{{\mathfrak{L}}_{\upsilon \nu }}^{U+}\right\}\right], \\ \left[\underset{\nu }{{\text{min}}}\,\underset{\upsilon }{{\text{min}}}\,\left\{{\rm N}_{{\mathfrak{L}}_{\upsilon \nu }}^{L-}\right\}, \underset{\nu }{{\text{min}}}\,\underset{\upsilon }{{\text{min}}}\,\left\{{\rm O}_{{\mathfrak{L}}_{\upsilon \nu }}^{U-}\right\}\right]+\\ \iota \left[\underset{\nu }{{\text{min}}}\,\underset{\upsilon }{{\text{min}}}\,\left\{{\rm N}_{{\mathfrak{L}}_{\upsilon \nu }}^{L-}\right\}, \underset{\nu }{{\text{min}}}\,\underset{\upsilon }{{\text{min}}}\,\left\{{\rm O}_{{\mathfrak{L}}_{\upsilon \nu }}^{U-}\right\}\right]\end{array}\right)$$

Then,

18 $$ {\mathfrak{L}}_{\upsilon \nu }^{ - } \le IVBCFSWGA\left( {{\mathfrak{L}}_{11} , {\mathfrak{L}}_{12} , \ldots , {\mathfrak{L}}_{{{\tilde{r}} {\tilde{s}} }} } \right) \le {\mathfrak{L}}_{\upsilon \nu }^{ + } $$

#### Proof

As like to Theorem 3.

#### Theorem 9

(Shift-invariance property): If

$${\mathfrak{L}} = \left( {{\rm E}_{{\mathfrak{L}}}^{ + } , {\rm E}_{{\mathfrak{L}}}^{ - } } \right) = \left( {\begin{array}{*{20}c} {\left[ {{\rm N}_{{\mathfrak{L}}}^{L + } , {\rm O}_{{\mathfrak{L}}}^{U + } } \right] + \iota \left[ {{\rm N}_{{\mathfrak{L}}}^{L + } , {\rm O}_{{\mathfrak{L}}}^{U + } } \right], } \\ {\left[ {{\rm N}_{{\mathfrak{L}}}^{L - } , {\rm O}_{{\mathfrak{L}}}^{U - } } \right] + \iota \left[ {{\rm N}_{{\mathfrak{L}}}^{L - } , {\rm O}_{{\mathfrak{L}}}^{U - } } \right]} \\ \end{array} } \right)\left( {\upsilon = 1, 2, \ldots ,{\tilde{r}} ; \nu = 1, 2, \ldots , {\tilde{s}} } \right)$$

be another IVBCFSN, then

$$ IVBCFSWGA\left( {{\mathfrak{L}}_{11} \oplus {\mathfrak{L}},{ }{\mathfrak{L}}_{12} \oplus {\mathfrak{L}},{ } \ldots ,{ }{\mathfrak{L}}_{{{\tilde{r}} {\tilde{s}} }} \oplus {\mathfrak{L}}} \right) = IVBCFSWAA\left( {{\mathfrak{L}}_{11} , {\mathfrak{L}}_{12} , \ldots , {\mathfrak{L}}_{{{\tilde{r}} {\tilde{s}} }} } \right) \oplus {\mathfrak{L}} $$

#### Proof

As like to Theorem 4.

#### Theorem 10

(Homogeneity property): For any real number $\chi >0$, we have

$$ IVBCFSWGA\left( {\chi {\mathfrak{L}}_{11} , \chi {\mathfrak{L}}_{12} , \ldots , \chi {\mathfrak{L}}_{{{\tilde{r}} {\tilde{s}} }} } \right) = \chi IVBCFSWGA\left( {{\mathfrak{L}}_{11} , {\mathfrak{L}}_{12} , \ldots , {\mathfrak{L}}_{{{\tilde{r}} {\tilde{s}} }} } \right) $$

#### Proof

As like to Theorem 5.

## Multi-attribute decision making technique

IVBCFSS has a wide range of uses to deal with the ambiguity and uncertainty that we express in our various real-world problems. In this section, we represent a DM method for elaborating on material in the context of IVBCFSSs and put it to use solving real-world DM issues.

### DM process

1. By using $\k{\rm U}$ is the set of universe and ${\mathring{\text{A}}} \subseteq \Upsilon$ be the set of attribute.
2. Take IVBCFSS in the presentation of tabular form
3. Make single table for every LPTG, UPTG, LNTG, and NTG
4. Analyze the comparison tables for LPTG, UPTG, LNTG, and UNTG
5. Explore the score of each LPTG, UPTG, LNTG, and UNTG table
6. Compute the ultimate score by subtracting the PTG score from NTG score
7. Select the greater score, if it appears in $\mathfrak{a}{\text{th}}$ row, then ${\iota }_{\mathfrak{a}}$ will be the best ideal.

### Demonstrated example (set-up 1)

Suppose a man want to buy a innovative mobile for his trade and consider four dissimilar mobiles i.e. $\k{\rm U} = \left\{ {\imath_{1} , \imath_{2} , \imath_{3} , \imath_{4} } \right\}$ further with the three dissimilar attributes i.e. ${\mathring{\text{A}}} =\left\{{j}_{1}=battery\; timing, {j}_{2}=operating \; system, {j}_{3}=storage\; space\right\}\subseteq \Upsilon$. He desires to purchase the best mobile device under these criteria. The information was presented in the context of the IVBCFSSs below

$$\left(\mathfrak{L},\Gamma \right)=\left\{\begin{array}{c}\mathfrak{L}\left({j}_{1}\right)=\left\{\begin{array}{c}\left({\iota }_{1}, \left[0.12, 0.25\right]+\iota \left[0.32, 0.43\right], \left[-0.23, -0.16\right]+\iota \left[-0.51, -0.24\right]\right),\\ \left({\iota }_{2}, \left[0.19, 0.51\right]+\iota \left[0.31, 0.42\right], \left[-0.62, -0.33\right]+\iota \left[-0.43, -0.31\right]\right) \\ \left({\iota }_{3}, \left[0.34, 0.45\right]+\iota \left[0.25, 0.36\right], \left[-0.56, -0.47\right]+\iota \left[-0.67, -0.57\right]\right), \\ \left({\iota }_{4}, \left[0.58, 0.65\right]+\iota \left[0.29, 0.31\right], \left[-0.81, -0.71\right]+\iota \left[-0.62, -0.23\right]\right), \end{array}\right\}\\ \mathfrak{L}\left({j}_{2}\right)=\left\{\begin{array}{c}\left({\iota }_{1}, \left[0.12, 0.23\right]+\iota \left[0.33, 0.43\right], \left[-0.64, -0.54\right]+\iota \left[-0.84, -0.75\right]\right),\\ \left({\iota }_{2}, \left[0.85, 0.95\right]+\iota \left[0.76, 0.86\right], \left[-0.66, -0.57\right]+\iota \left[-0.47, -0.37\right]\right), \\ \left({\iota }_{3}, \left[0.18, 0.28\right]+\iota \left[0.18, 0.29\right], \left[-0.49, -0.39\right]+\iota \left[-0.61, -0.51\right]\right), \\ \left({\iota }_{4}, \left[0.71, 0.81\right]+\iota \left[0.82, 0.92\right], \left[-0.82, -0.72\right]+\iota \left[-0.63, -0.53\right]\right)\end{array}\right\}\\ \mathfrak{L}\left({j}_{3}\right)=\left\{\begin{array}{c}\left({\iota }_{1}, \left[0.43, 0.53\right]+\iota \left[0.26, 0.36\right], \left[-0.24, -0.15\right]+\iota \left[-0.45, -0.35\right]\right), \\ \left({\iota }_{2}, \left[0.55, 0.67\right]+\iota \left[0.76, 0.86\right], \left[-0.97, -0.87\right]+\iota \left[-0.67, -0.57\right]\right), \\ \left({\iota }_{3}, \left[0.67, 0.77\right]+\iota \left[0.71, 0.81\right], \left[-0.28, -0.19\right]+\iota \left[-0.29, -0.19\right]\right), \\ \left({\iota }_{4}, \left[0.39, 0.41\right]+\iota \left[0.51, 0.61\right], \left[-0.82, -0.73\right]+\iota \left[-0.93, -0.87\right]\right)\end{array}\right\}\end{array}\right\}$$

The tabular representation of IVBCFSS interpreted in set-up 1 is depicted in Table 3.

**Table 3:** The tabular representation of IVBCFSS in set-up 1.

$\left(\mathfrak{L},\Gamma \right)$	${j}_{1}$	${j}_{2}$	${j}_{3}$
${\iota }_{1}$	$$\left(\begin{array}{c}\left[0.12, 0.25\right]+\iota \left[0.32, 0.43\right], \\ \left[-0.23, -0.16\right]+\iota \left[-0.51, -0.24\right]\end{array}\right)$$	$$\left(\begin{array}{c}\left[0.12, 0.23\right]+\iota \left[0.33, 0.43\right], \\ \left[-0.64, -0.54\right]+\iota \left[-0.84, -0.75\right]\end{array}\right)$$	$$\left(\begin{array}{c}\left[0.43, 0.53\right]+\iota \left[0.26, 0.36\right],\\ \left[-0.24, -0.15\right]+\iota \left[-0.45, -0.35\right]\end{array}\right)$$
${\iota }_{2}$	$$\left(\begin{array}{c}\left[0.19, 0.51\right]+\iota \left[0.31, 0.42\right],\\ \left[-0.62, -0.33\right]+\iota \left[-0.43, -0.31\right]\end{array}\right)$$	$$\left(\begin{array}{c}\left[0.85, 0.95\right]+\iota \left[0.76, 0.86\right], \\ \left[-0.66, -0.57\right]+\iota \left[-0.47, -0.37\right]\end{array}\right)$$	$$\left(\begin{array}{c}\left[0.55, 0.67\right]+\iota \left[0.76, 0.86\right], \\ \left[-0.97, -0.87\right]+\iota \left[-0.67, -0.57\right]\end{array}\right)$$
${\iota }_{3}$	$$\left(\begin{array}{c}\left[0.34, 0.45\right]+\iota \left[0.25, 0.36\right], \\ \left[-0.56, -0.47\right]+\iota \left[-0.67, -0.57\right]\end{array}\right)$$	$$\left(\begin{array}{c}\left[0.18, 0.28\right]+\iota \left[0.18, 0.29\right], \\ \left[-0.49, -0.39\right]+\iota \left[-0.61, -0.51\right]\end{array}\right)$$	$$\left(\begin{array}{c}\left[0.67, 0.77\right]+\iota \left[0.71, 0.81\right], \\ \left[-0.28, -0.19\right]+\iota \left[-0.29, -0.19\right]\end{array}\right)$$
${\iota }_{4}$	$$\left(\begin{array}{c}\left[0.58, 0.65\right]+\iota \left[0.29, 0.31\right], \\ \left[-0.81, -0.71\right]+\iota \left[-0.62, -0.23\right]\end{array}\right)$$	$$\left(\begin{array}{c}\left[0.71, 0.81\right]+\iota \left[0.82, 0.92\right], \\ \left[-0.82, -0.72\right]+\iota \left[-0.63, -0.53\right]\end{array}\right)$$	$$\left(\begin{array}{c}\left[0.39, 0.41\right]+\iota \left[0.51, 0.61\right], \\ \left[-0.82, -0.73\right]+\iota \left[-0.93, -0.87\right]\end{array}\right)$$

Here, our purpose to select best mobile. The tabular form for four LPTG, UPTG, LNTG, and UNTG are specified in Tables 4, 5, 6, and 7 respectively.

**Table 4:** The tabular representation LPTG set-up 1.

	${j}_{1}$	${j}_{2}$	${j}_{3}$
${\iota }_{1}$	$0.12+\iota 0.32$	$0.12+\iota 0.33$	$0.43+\iota 0.26$
${\iota }_{2}$	$0.19+\iota 0.31$	$0.85+\iota 0.76$	$0.55+\iota 0.76$
${\iota }_{3}$	$0.34+\iota 0.25$	$0.18+\iota 0.18$	$0.67+\iota 0.71$
${\iota }_{4}$	$0.58+\iota 0.29$	$0.71+\iota 0.82$	$0.39+\iota 0.51$

**Table 5:** The tabular representation UPTG set-up 1.

	${j}_{1}$	${j}_{2}$	${j}_{3}$
${\iota }_{1}$	$0.25+\iota 0.43$	$0.23+\iota 0.43$	$0.53+\iota 0.36$
${\iota }_{2}$	$0.51+\iota 0.42$	$0.85+\iota 0.86$	$0.67+\iota 0.86$
${\iota }_{3}$	$0.45+\iota 0.36$	$0.28+\iota 0.29$	$0.77+\iota 0.81$
${\iota }_{4}$	$0.65+\iota 0.31$	$0.81+\iota 0.92$	$0.41+\iota 0.61$

**Table 6:** The tabular representation LNTG set-up 1.

	${j}_{1}$	${j}_{2}$	${j}_{3}$
${\iota }_{1}$	$-0.23-\iota 0.51$	$-0.64-\iota 0.84$	$-0.24-\iota 0.45$
${\iota }_{2}$	$-0.62-\iota 0.43$	$-0.66-\iota 0.47$	$-0.97-\iota 0.67$
${\iota }_{3}$	$-0.56-\iota 0.67$	$-0.49-\iota 0.61$	$-0.28-\iota 0.29$
${\iota }_{4}$	$-0.81-\iota 0.62$	$-0.82-\iota 0.63$	$-0.82-\iota 0.93$

**Table 7:** The tabular representation UNTG set-up 1.

	${j}_{1}$	${j}_{2}$	${j}_{3}$
${\iota }_{1}$	$-0.16-\iota 0.24$	$-0.54-\iota 0.75$	$-0.15-\iota 0.35$
${\iota }_{2}$	$-0.33-\iota 0.31$	$-0.57-\iota 0.37$	$-0.87-\iota 0.57$
${\iota }_{3}$	$-0.47-\iota 0.57$	$-0.39-\iota 0.51$	$-0.19-\iota 0.19$
${\iota }_{4}$	$-0.71-\iota 0.23$	$-0.72-\iota 0.53$	$-0.73-\iota 0.87$

The comparison tables for four LPTG, UPTG, LNTG and UNTG are specified in Tables 8, 9, 10, and 11 respectively.

**Table 8:** The comparison table for LPTG set-up 1.

	${\iota }_{1}$	${\iota }_{2}$	${\iota }_{3}$	${\iota }_{4}$
${\iota }_{1}$	3	0	0	1
${\iota }_{2}$	3	3	1	2
${\iota }_{3}$	3	2	3	1
${\iota }_{4}$	2	1	2	3

**Table 9:** The comparison table for UPTG set-up 1.

	${\iota }_{1}$	${\iota }_{2}$	${\iota }_{3}$	${\iota }_{4}$
${\iota }_{1}$	3	0	0	1
${\iota }_{2}$	3	3	2	2
${\iota }_{3}$	3	1	3	1
${\iota }_{4}$	2	1	2	3

**Table 10:** The comparison table for LNTG set-up 1.

	${\iota }_{1}$	${\iota }_{2}$	${\iota }_{3}$	${\iota }_{4}$
${\iota }_{1}$	3	3	2	3
${\iota }_{2}$	0	3	0	2
${\iota }_{3}$	1	3	3	3
${\iota }_{4}$	0	1	0	3

**Table 11:** The comparison table for UNTG set-up 1.

	${\iota }_{1}$	${\iota }_{2}$	${\iota }_{3}$	${\iota }_{4}$
${\iota }_{1}$	3	3	2	3
${\iota }_{2}$	0	3	1	2
${\iota }_{3}$	1	2	3	3
${\iota }_{4}$	0	1	0	3

The scores of LPTG, UPTG, LNTG and UNTG are specified in Tables 12, 13, 14, and 15 respectively.

**Table 12:** The tabular representation LPTG set-up 1.

	$Row\;Sum$ RS	$Column\;Sum$ CS	$Lower\;positive\;score$ RS − CS
${\iota }_{1}$	$4$	$11$	$-7$
${\iota }_{2}$	$9$	$6$	$3$
${\iota }_{3}$	$9$	$6$	$3$
${\iota }_{4}$	$8$	$7$	$1$

**Table 13:** The tabular representation UPTG set-up 1.

	$Row\;Sum$ RS	$Column\;Sum$ CS	$Upper\;positive\;score$ RS − CS
${\iota }_{1}$	$4$	$11$	$-7$
${\iota }_{2}$	$10$	$5$	$5$
${\iota }_{3}$	$8$	$7$	$1$
${\iota }_{4}$	$8$	$7$	$1$

**Table 14:** The tabular representation LNTG set-up 1.

	$Row\;Sum$ RS	$Column\;Sum$ CS	$Lower\;negative\;score$ RS − CS
${\iota }_{1}$	$11$	$4$	$7$
${\iota }_{2}$	$5$	$10$	$-5$
${\iota }_{3}$	$10$	$6$	$4$
${\iota }_{4}$	$4$	$11$	$-7$

**Table 15:** The tabular representation UNTG set-up 1.

	$Row\;Sum$ RS	$Column\;Sum$ CS	$Upper\;negative\;score$ RS − CS
${\iota }_{1}$	$11$	$4$	$7$
${\iota }_{2}$	$6$	$9$	$-3$
${\iota }_{3}$	$9$	$6$	$3$
${\iota }_{4}$	$4$	$11$	$-7$

In Table 16, we demonstrate final score of Tables 12 and 13 respectively.

**Table 16:** Positive final score table set-up 1.

	$Lower\;positive\;score$	$Upper\;positive\;score$	$Final \;score$ UPs − LPs
${\iota }_{1}$	$-7$	$-7$	$0$
${\iota }_{2}$	$3$	$5$	$2$
${\iota }_{3}$	$3$	$1$	$-2$
${\iota }_{4}$	$1$	$1$	$0$

In Table 17, we demonstrate final score of Tables 14 and 15 respectively.

**Table 17:** Negative final score table set-up 1.

	$Lower\;negative\;score$	$Upper\;negative\;score$	$Final\;score$ UNs − LNs
${\iota }_{1}$	$7$	$7$	$0$
${\iota }_{2}$	$-5$	$-3$	$2$
${\iota }_{3}$	$4$	$3$	$-1$
${\iota }_{4}$	$-7$	$-7$	$0$

*Decision* the man will purchase the mobile ${\iota }_{2}$ is best option in Table 16. In the rest of some reasons if he doesn’t desire to buy the mobile ${\iota }_{2}$, he will buy ${\iota }_{1}$ or ${\iota }_{4}$ because ${\iota }_{1}$ and ${\iota }_{4}$ are the further best options in view of Table 16.

The man will purchase the mobile ${\iota }_{2}$ in Table 17. In the rest of some reasons if he doesn’t desire to buy the mobile ${\iota }_{2}$, he will buy ${\iota }_{1}$ or ${\iota }_{4}$ because buy ${\iota }_{1}$ and ${\iota }_{4}$ are the further best options in view of Table 17. By the inspection of above both tables should be decide the best option of ${\iota }_{2}$ and further 2^nd^ options are same in above both Tables 16 and 17.

### Demonstrated example (set-up 2)

Suppose a professor want to recruit a research assistant for helping in his research area and consider four dissimilar applicants i.e. $\k{\rm U} = \left\{ {\imath_{1} , \imath_{2} , \imath_{3} , \imath_{4} } \right\}$ further with the three dissimilar attributes i.e. ${\mathring{\text{A}}} =\left\{{j}_{1}=creative person, {j}_{2}=hardworking person, {j}_{3}=regular person\right\}\subseteq \Upsilon$. He desires to select the best applicant under these criteria. The information was presented in the context of the IVBCFSSs below

$$\left(\mathfrak{L},\Gamma \right)=\left\{\begin{array}{c}\mathfrak{L}\left({j}_{1}\right)=\left\{\begin{array}{c}\left({\iota }_{1}, \left[0.22, 0.35\right]+\iota \left[0.42, 0.53\right], \left[-0.33, -0.26\right]+\iota \left[-0.61, -0.34\right]\right),\\ \left({\iota }_{2}, \left[0.17, 0.41\right]+\iota \left[0.29, 0.39\right], \left[-0.59, -0.43\right]+\iota \left[-0.53, -0.21\right]\right) \\ \left({\iota }_{3}, \left[0.36, 0.47\right]+\iota \left[0.27, 0.38\right], \left[-0.58, -0.49\right]+\iota \left[-0.69, -0.59\right]\right), \\ \left({\iota }_{4}, \left[0.56, 0.63\right]+\iota \left[0.31, 0.41\right], \left[-0.91, -0.81\right]+\iota \left[-0.63, -0.33\right]\right), \end{array}\right\}\\ \mathfrak{L}\left({j}_{2}\right)=\left\{\begin{array}{c}\left({\iota }_{1}, \left[0.32, 0.43\right]+\iota \left[0.23, 0.53\right], \left[-0.66, -0.56\right]+\iota \left[-0.81, -0.85\right]\right),\\ \left({\iota }_{2}, \left[0.81, 0.97\right]+\iota \left[0.86, 0.96\right], \left[-0.76, -0.67\right]+\iota \left[-0.37, -0.27\right]\right), \\ \left({\iota }_{3}, \left[0.13, 0.38\right]+\iota \left[0.28, 0.39\right], \left[-0.59, -0.49\right]+\iota \left[-0.71, -0.61\right]\right), \\ \left({\iota }_{4}, \left[0.81, 0.91\right]+\iota \left[0.72, 0.82\right], \left[-0.62, -0.52\right]+\iota \left[-0.73, -0.63\right]\right)\end{array}\right\}\\ \mathfrak{L}\left({j}_{3}\right)=\left\{\begin{array}{c}\left({\iota }_{1}, \left[0.63, 0.73\right]+\iota \left[0.36, 0.46\right], \left[-0.34, -0.25\right]+\iota \left[-0.55, -0.45\right]\right), \\ \left({\iota }_{2}, \left[0.65, 0.77\right]+\iota \left[0.66, 0.76\right], \left[-0.87, -0.97\right]+\iota \left[-0.77, -0.67\right]\right), \\ \left({\iota }_{3}, \left[0.68, 0.79\right]+\iota \left[0.74, 0.85\right], \left[-0.26, -0.17\right]+\iota \left[-0.28, -0.19\right]\right), \\ \left({\iota }_{4}, \left[0.31, 0.42\right]+\iota \left[0.53, 0.64\right], \left[-0.85, -0.76\right]+\iota \left[-0.97, -0.88\right]\right)\end{array}\right\}\end{array}\right\}$$

The tabular representation of IVBCFSS interpreted in set-up 2 is depicted in Table 18.

**Table 18:** The tabular representation of IVBCFSS in set-up 2.

$\left(\mathfrak{L},\Gamma \right)$	${j}_{1}$	${j}_{2}$	${j}_{3}$
${\iota }_{1}$	$$\left(\begin{array}{c}\left[0.22, 0.35\right]+\iota \left[0.42, 0.53\right], \\ \left[-0.33, -0.26\right]+\iota \left[-0.61, -0.34\right]\end{array}\right)$$	$$\left(\begin{array}{c}\left[0.32, 0.43\right]+\iota \left[0.23, 0.53\right], \\ \left[-0.66, -0.56\right]+\iota \left[-0.81, -0.85\right]\end{array}\right)$$	$$\left(\begin{array}{c}\left[0.63, 0.73\right]+\iota \left[0.36, 0.46\right], \\ \left[-0.34, -0.25\right]+\iota \left[-0.55, -0.45\right]\end{array}\right)$$
${\iota }_{2}$	$$\left(\begin{array}{c}\left[0.17, 0.41\right]+\iota \left[0.29, 0.39\right],\\ \left[-0.59, -0.43\right]+\iota \left[-0.53, -0.21\right]\end{array}\right)$$	$$\left(\begin{array}{c}\left[0.81, 0.97\right]+\iota \left[0.86, 0.96\right], \\ \left[-0.76, -0.67\right]+\iota \left[-0.37, -0.27\right]\end{array}\right)$$	$$\left(\begin{array}{c}\left[0.65, 0.77\right]+\iota \left[0.66, 0.76\right],\\ \left[-0.87, -0.97\right]+\iota \left[-0.77, -0.67\right]\end{array}\right)$$
${\iota }_{3}$	$$\left(\begin{array}{c}\left[0.36, 0.47\right]+\iota \left[0.27, 0.38\right], \\ \left[-0.58, -0.49\right]+\iota \left[-0.69, -0.59\right]\end{array}\right)$$	$$\left(\begin{array}{c}\left[0.13, 0.38\right]+\iota \left[0.28, 0.39\right],\\ \left[-0.59, -0.49\right]+\iota \left[-0.71, -0.61\right]\end{array}\right)$$	$$\left(\begin{array}{c}\left[0.68, 0.79\right]+\iota \left[0.74, 0.85\right],\\ \left[-0.26, -0.17\right]+\iota \left[-0.28, -0.19\right]\end{array}\right)$$
${\iota }_{4}$	$$\left(\begin{array}{c}\left[0.56, 0.63\right]+\iota \left[0.31, 0.41\right], \\ \left[-0.91, -0.81\right]+\iota \left[-0.63, -0.33\right]\end{array}\right)$$	$$\left(\begin{array}{c}\left[0.81, 0.91\right]+\iota \left[0.72, 0.82\right], \\ \left[-0.62, -0.52\right]+\iota \left[-0.73, -0.63\right]\end{array}\right)$$	$$\left(\begin{array}{c}\left[0.31, 0.42\right]+\iota \left[0.53, 0.64\right],\\ \left[-0.85, -0.76\right]+\iota \left[-0.97, -0.88\right]\end{array}\right)$$

Here, our purpose to select best applicant. The tabular form for four LPTG, UPTG, LNTG and UNTG are specified in Tables 19, 20, 21, and 22 respectively.

**Table 19:** The tabular representation LPTG set-up 2.

	${j}_{1}$	${j}_{2}$	${j}_{3}$
${\iota }_{1}$	$0.22+\iota 0.42$	$0.32+\iota 0.23$	$0.63+\iota 0.36$
${\iota }_{2}$	$0.17+\iota 0.29$	$0.81+\iota 0.86$	$0.65+\iota 0.66$
${\iota }_{3}$	$0.36+\iota 0.27$	$0.13+\iota 0.28$	$0.68+\iota 0.74$
${\iota }_{4}$	$0.56+\iota 0.31$	$0.81+\iota 0.72$	$0.31+\iota 0.53$

**Table 20:** The tabular representation UPTG set-up 2.

	${j}_{1}$	${j}_{2}$	${j}_{3}$
${\iota }_{1}$	$0.35+\iota 0.53$	$0.43+\iota 0.53$	$0.73+\iota 0.46$
${\iota }_{2}$	$0.41+\iota 0.39$	$0.97+\iota 0.96$	$0.77+\iota 0.76$
${\iota }_{3}$	$0.47+\iota 0.27$	$0.38+\iota 0.39$	$0.79+\iota 0.85$
${\iota }_{4}$	$0.63+\iota 0.41$	$0.91+\iota 0.82$	$0.42+\iota 0.64$

**Table 21:** The tabular representation LNTG set-up 2.

	${j}_{1}$	${j}_{2}$	${j}_{3}$
${\iota }_{1}$	$-0.33-\iota 0.61$	$-0.66-\iota 0.81$	$-0.34-\iota 0.55$
${\iota }_{2}$	$-0.59-\iota 0.53$	$-0.76-\iota 0.37$	$-0.87-\iota 0.77$
${\iota }_{3}$	$-0.58-\iota 0.69$	$-0.59-\iota 0.71$	$-0.26-\iota 0.28$
${\iota }_{4}$	$-0.91-\iota 0.63$	$-0.62-\iota 0.73$	$-0.85-\iota 0.97$

**Table 22:** The tabular representation UNTG set-up 2.

	${j}_{1}$	${j}_{2}$	${j}_{3}$
${\iota }_{1}$	$-0.26-\iota 0.34$	$-0.56-\iota 0.85$	$-0.25-\iota 0.45$
${\iota }_{2}$	$-0.43-\iota 0.21$	$-0.67-\iota 0.27$	$-0.97-\iota 0.67$
${\iota }_{3}$	$-0.49-\iota 0.59$	$-0.49-\iota 0.61$	$-0.17-\iota 0.19$
${\iota }_{4}$	$-0.81-\iota 0.63$	$-0.52-\iota 0.63$	$-0.76-\iota 0.88$

The comparison tables for four LPTG, UPTG, LNTG and UNTG are specified in Tables 23, 24, 25, and 26 respectively.

**Table 23:** The comparison table for LPTG set-up 2.

	${\iota }_{1}$	${\iota }_{2}$	${\iota }_{3}$	${\iota }_{4}$
${\iota }_{1}$	3	1	1	1
${\iota }_{2}$	2	3	1	2
${\iota }_{3}$	2	2	3	1
${\iota }_{4}$	2	2	2	3

**Table 24:** The comparison table for UPTG set-up 2.

	${\iota }_{1}$	${\iota }_{2}$	${\iota }_{3}$	${\iota }_{4}$
${\iota }_{1}$	3	0	1	1
${\iota }_{2}$	3	3	1	2
${\iota }_{3}$	2	2	3	1
${\iota }_{4}$	2	1	2	3

**Table 25:** The comparison table for LNTG set-up 2.

	${\iota }_{1}$	${\iota }_{2}$	${\iota }_{3}$	${\iota }_{4}$
${\iota }_{1}$	3	3	1	2
${\iota }_{2}$	0	3	0	1
${\iota }_{3}$	2	2	3	3
${\iota }_{4}$	1	2	0	3

**Table 26:** The comparison table for UNTG set-up 2.

	${\iota }_{1}$	${\iota }_{2}$	${\iota }_{3}$	${\iota }_{4}$
${\iota }_{1}$	3	3	1	2
${\iota }_{2}$	0	3	1	1
${\iota }_{3}$	2	2	3	3
${\iota }_{4}$	1	2	0	3

The scores of LPTG, UPTG, LNTG and UNTG are specified in Tables 27, 28, 29, and 30 respectively.

**Table 27:** The tabular representation LPTG set-up 1.

	$Row sum$ RS	$Column sum$ CS	$Lower positive score$ RS − CS
${\iota }_{1}$	$6$	$9$	$- \,3$
${\iota }_{2}$	$8$	$8$	$0$
${\iota }_{3}$	$8$	$7$	$1$
${\iota }_{4}$	$9$	$7$	$2$

**Table 28:** The tabular representation UPTG set-up 1.

	$Row sum$ RS	$Column sum$ CS	$Upper positive score$ RS − CS
$\imath_{1}$	$5$	$10$	$- \,5$
$\imath_{2}$	$9$	$6$	$3$
$\imath_{3}$	$8$	$7$	$1$
$\imath_{4}$	$8$	$7$	$1$

**Table 29:** The tabular representation LNTG set-up 2.

	$Row\;sum$ RS	$Column\;sum$ CS	$Lower\;negative\;score$ RS − CS
$\imath_{1}$	9	$6$	$3$
$\imath_{2}$	$4$	10	$- 6$
$\imath_{3}$	$10$	4	$6$
$\imath_{4}$	$6$	$9$	$- 3$

**Table 30:** The tabular representation UNTG set-up 2.

	$$Row \;sum$$ RS	$$Column\;sum$$ CS$$4$$$$6$$$$Upper\;negative\;score$$	RS$$9$$ − CS
$\imath_{1}$	$9$	6	$3$
$\imath_{2}$	$5$	$10$	$- \,5$
$\imath_{3}$	$10$	5	$5$
$\imath_{4}$	$6$	$9$	$- \,3$

In Table 31, we demonstrate final score of Tables 27 and 28 respectively.

**Table 31:** Positive final score table set-up 2.

	$Lower\;positive\;score$	$Upper\;positive\;score$	$Final\;score$ UPs − LPs
$\imath_{1}$	$- \,3$	$- \,5$	$- \,2$
$\imath_{2}$	$0$	$3$	$3$
$\imath_{3}$	$1$	$1$	$0$
$\imath_{4}$	$2$	$1$	$- \,1$

In Table 32, we demonstrate final score of Tables 29 and 30 respectively.

**Table 32:** Negative final score table set-up 2.

	$Lower\;negative\;score$	$Upper\;negative\;score$	$Final\;score$ UNs − LNs
$\imath_{1}$	$3$	$3$	$0$
$\imath_{2}$	$- \,6$	$- \,5$	$1$
$\imath_{3}$	$6$	$5$	.$- \,1$
$\imath_{4}$	$- \,3$	$- \,3$	$0$

*Decision* the professor will select the applicant ${\iota }_{2}$ is the best option in Table 31. In the rest of some reasons if he doesn’t desire to select applicant ${\iota }_{2}$, he will select ${\iota }_{3}$ because ${\iota }_{3}$ is the further best applicant in view of Table 31.

The professor will applicant ${\iota }_{2}$ is the best option in Table 32. In the rest of some reasons if he doesn’t desire to select ${\iota }_{2}$, he will select ${\iota }_{1}$ or ${\iota }_{4}$ because ${\iota }_{1}$ and ${\iota }_{4}$ are the further best applicant’s options and are also applicable for selection in view of Table 32. By the inspection of above both tables should be decide the best applicant ${\iota }_{2}$, which is approved from above both result tables.

## Comparative analysis

Firstly, the expert arrange data or materials in the form of IVBCFSSs then there doesn’t satisfied the prevailing concepts which can hold this materials or data. The above demonstrated idea IVBCFSSs are exist the data and provided a lot of information for the decision maker. Decision maker can easily sort out all hurdles which are facing in prevailing ideas. In this idea, we signified the generalization of prevailing notions like SS^11^, FS^4^, FSS^12^, BFSS^16^ and BCFSSs^17^ as follows.

1. If we remove the upper and lower approximation then the demonstrated IVBCFSSs will degenerated to BCFSSs.
2. If we remove the upper, lower approximation and NTG then the demonstrated IVBCFSSs will degenerated to CFSSs.
3. If we remove the upper, lower approximation, parameter and NTG then the demonstrated IVBCFSSs will degenerated to CFSs.
4. If we remove the upper, lower approximation, parameter, amplitude term and NTG then the demonstrated IVBCFSSs will degenerated to FSs.
5. If we remove the upper, lower approximation, amplitude term, NTG and FS then the demonstrated IVBCFSSs will degenerated to SS.

Our suggested approach can satisfy this kind of data and be more beneficial in problem modelling if a decision maker gathers the data in the form of the aforementioned prevalent notions. The IVBCFSS is a crucial and effective tool for gaining satisfaction and efficiency in order to grasp difficult and ambiguous types of theory in everyday problems. As a result of the fusion of four concepts—parameterization, complex fuzziness, bipolarity, and interval aspects—the mathematical structure of the IVBCFSS is more altered and appealing than it would be if the concepts were used alone, as was the case with the prevailing concepts listed above.

## Conclusion

This article officially confirms two terms like IVBCFS and IVBCFSS. Those are the mixture of IVBFS, and CFSS. Here, we also match the established work with dominant notions, like FS, SS, CFS, FSS, BFS, BFSS, BCFS, and BCFSS. We invented IVBCFS and IVBCFSS by adding lower and upper approximations to BCFS and BCFSS. Moreover, BCFSS is required more extension in the novel concept. In the notion of IVBCFS, we demonstrated some operations like complement, union and intersection of IVBCFS with their examples. In the concept of IVBCFSS, we illustrated the significance of IVBCFSS and its applications. In addition, we included examples for the basic operations complement, extended union, extended intersection, restricted union, and restricted intersection. With worked examples, we also presented the OR and AND operations here. Moreover, we also demonstrated some fundamental aggregation operators (AOs) like IVBCFS average aggregation (IVBCFSAA), IVBCFS geometric (IVBCFSGA) operators and with their properties. Additionally, we interpreted the DM method and common instances (set-up 1 and set-up 2) of IVBCFSS to highlight the success and use of the exhibited IVBCFSS. Finally, this study also interprets the efficiency and potency of the established work by a relative investigation of established thought with prevalent ideas. In the IVBCFSSs, we can improve this by applying Dombi AOs, Hamacher AOs and similarity measures and so on.

## Data Availability

The data will be available on reasonable request to corresponding author.

## References

[1] Edwards W (1954). The theory of decision making. Psychol. Bull..

[2] Slovic P, Lichtenstein S, Fischhoff B (1988). Decision Making.

[3] Eisenhardt KM, Zbaracki MJ (1992). Strategic decision making. Strateg. Manag. J..

[4] March JG (1994). Primer on Decision Making: How Decisions Happen.

[5] Zadeh LA (1965). Fuzzy sets. Inf. Control.

[6] Ramot D, Milo R, Friedman M, Kandel A (2002). Complex fuzzy sets. IEEE Trans. Fuzzy Syst..

[7] Tamir DE, Rishe ND, Kandel A, Tamir DE, Rishe ND, Kandel A (2015). Complex fuzzy sets and complex fuzzy logic an overview of theory and applications. Fifty Years of Fuzzy Logic and Its Applications.

[8] Zhang, W. R. (Yin)(Yang) bipolar fuzzy sets. in *1998 IEEE International Conference on Fuzzy Systems Proceedings. IEEE World Congress on Computational Intelligence (Cat. No. 98CH36228) *Vol. 1, pp. 835–840 (IEEE, 1998).

[9] Mahmood T, Ur Rehman U (2022). A novel approach towards bipolar complex fuzzy sets and their applications in generalized similarity measures. Int. J. Intell. Syst..

[10] Mahmood T, Rehman UU, Ahmmad J, Santos-García G (2021). Bipolar complex fuzzy Hamacher aggregation operators and their applications in multi-attribute decision making. Mathematics.

[11] Mahmood T, Rehman UU (2022). A method to multi-attribute decision making technique based on Dombi aggregation operators under bipolar complex fuzzy information. Comput. Appl. Math..

[12] Molodtsov D (1999). Soft set theory—first results. Comput. Math. Appl..

[13] Cagman N, Enginoglu S, Citak F (2011). Fuzzy soft set theory and its applications. Iran. J. Fuzzy Syst..

[14] Çağman N, Karataş S (2013). Intuitionistic fuzzy soft set theory and its decision making. J. Intell. Fuzzy Syst..

[15] Dhumras H, Bajaj RK (2023). Modified EDAS method for MCDM in robotic agriculture farming with picture fuzzy soft Dombi aggregation operators. Soft Comput..

[16] Thirunavukarasu P, Suresh R, Ashokkumar V (2017). Theory of complex fuzzy soft set and its applications. Int. J. Innov. Res. Sci. Technol.

[17] Abdullah S, Aslam M, Ullah K (2014). Bipolar fuzzy soft sets and its applications in decision -making problem. J. Intell. Fuzzy Syst..

[18] Mahmood T, Rehman UU, Jaleel A, Ahmmad J, Chinram R (2022). Bipolar complex fuzzy soft sets and their applications in decision-making. Mathematics.

[19] Jaleel A (2022). WASPAS technique utilized for agricultural robotics system based on Dombi aggregation operators under bipolar complex fuzzy soft information. J. Innov. Res. Math. Comput. Sci..

[20] Zadeh LA (1975). The concept of a linguistic variable and its application to approximate reasoning—I. Inf. Sci..

[21] Nasir A, Jan N, Gumaei A, Khan SU, Albogamy FR (2021). Cyber security against the loop holes in industrial control systems using interval-valued complex intuitionistic fuzzy relations. Appl. Sci..

[22] Yang X, Lin TY, Yang J, Li Y, Yu D (2009). Combination of interval-valued fuzzy set and soft set. Comput. Math. Appl..

[23] Peng X, Dai J, Yuan H (2017). Interval-valued fuzzy soft decision making methods based on MABAC, similarity measure and EDAS. Fundam. Inform..

[24] Wei G, Wei C, Gao H (2018). Multiple attribute decision making with interval-valued bipolar fuzzy information and their application to emerging technology commercialization evaluation. IEEE Access.

[25] Feng F, Li Y, Leoreanu-Fotea V (2010). Application of level soft sets in decision making based on interval-valued fuzzy soft sets. Comput. Math. Appl..

[26] Hussain A, Bari M, Javed W (2022). Performance of the multi attributed decision-making process with interval-valued spherical fuzzy Dombi aggregation operators. J. Innov. Res. Math. Comput. Sci..

[27] Liaqat M, Yin S, Akram M, Ijaz S (2022). Aczel–Alsina aggregation operators based on interval-valued complex single-valued neutrosophic information and their application in decision-making problems. J. Innov. Res. Math. Comput. Sci..

